# Triple-helical aggregates of copper(i) cyclic trinuclear complexes for circularly polarized luminescence

**DOI:** 10.1039/d5sc04965b

**Published:** 2025-11-25

**Authors:** Guo-Quan Huang, Hu Yang, Ri-Qin Xia, Kun Wu, Yong-Liang Huang, De-Bo Hao, Shun-Bo Li, Weigang Lu, Ji Zheng, Xiao-Ping Zhou, Dan Li

**Affiliations:** a College of Chemistry and Materials Science, Guangdong Provincial Key Laboratory of Supramolecular Coordination Chemistry, Jinan University Guangzhou Guangdong 510632 P. R. China weiganglu@jnu.edu.cn jizheng@jnu.edu.cn zhouxp@jnu.edu.cn danli@jnu.edu.cn; b Department of Chemistry, Shantou University Medical College Shantou Guangdong 515041 P. R. China

## Abstract

Achieving chirality transfer and amplification through controlled supramolecular aggregation has long been a challenge in chemistry. This study presents the self-assembly of homochiral triple-helical aggregates (P- and M-type) with enantiomerically pure Cu(i) cyclic trinuclear complexes (CTCs) through metallophilic and hydrogen-bonding interactions. Both P- and M-type aggregates exhibit bright orange-red phosphorescence and circularly polarized luminescence (CPL) emission with an exceptional luminescence dissymmetry factor (*g*_lum_) of approximately ±1 × 10^−2^. These values are the highest among coinage metal-based complexes with emissions across the red and near-infrared. Experimental results and computational simulations reveal that the extensive overlap of chiral and luminescent centers is key to enabling CPL activity. Further installing naphthyl chromophore in the pyrazolate ligands results in isostructural triple-helical aggregates with rarely observed dual CPL emission behavior. Overall, this study showcases the successful construction of homochiral triple-helical aggregates by incorporating chiral centers and hydrogen-bonding sites into the peripheral pyrazolate ligands of Cu(i) CTCs, allowing for chirality transfer and amplification, evidenced by large *g*_lum_ values of CPL emission. These findings may facilitate the bottom-up design of homochiral supramolecular aggregates for CPL-related applications.

## Abstract 2

Self-assembly of Cu(i) complexes *via* enantiomeric helical stacking produces homochiral triple-helical aggregates, enabling a record high luminescence dissymmetry factor (|*g*_lum_| > 1 × 10^−2^) for coinage metal complexes in the red-NIR region.

## Graphical abstract

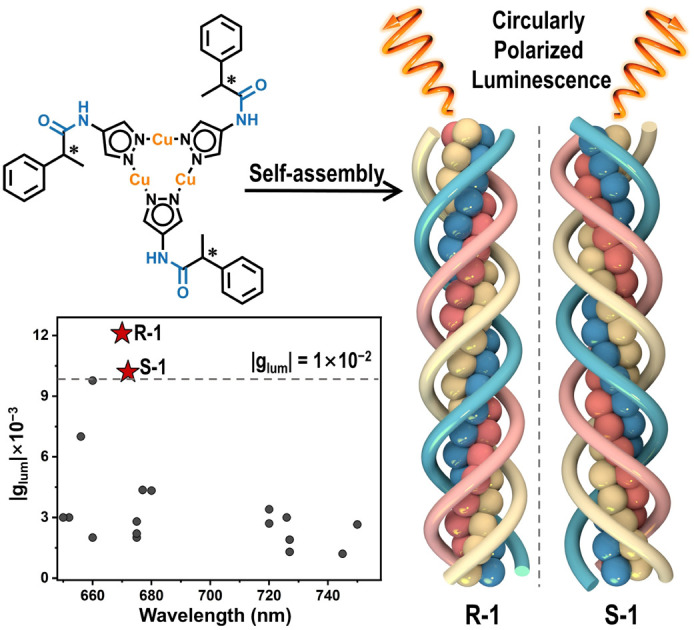

## Introduction

Circularly polarized luminescence (CPL), originating from chiral chromophores or chiral environments in the excited states,^1–5^ has garnered significant attention due to its potential applications in information encryption,^6,7^ asymmetric catalysis,^8,9^ biosensing,^10,11^*etc*. CPL activity can be evaluated by the luminescence dissymmetry factor (*g*_lum_), and achieving large *g*_lum_ values in luminescent materials through molecular design is a current research focus.^12,13^ However, the small transition magnetic dipole of most luminescent molecules impedes the advancement of CPL materials, where the |*g*_lum_| values are usually smaller than 1 × 10^−2^.^14–16^ Additionally, CPL silence may occur in chiral luminescent materials due to the disconnection between chiral and luminescent centers, restricting efficient chirality transfer in the excited states.^17^ Several strategies have been proposed to amplify the *g*_lum_ values, including Förster resonance energy transfer, charge transfer, liquid crystal doping, supramolecular self-assembly, *etc*.^14,18,19^ Among these, self-assembly seems particularly promising, as it can bypass tedious synthesis processes and allow for the formation of chiral aggregates with an ordered arrangement of luminescent molecules.^20–23^

Helix, a fascinating manifestation of chirality, has been observed across a wide range of scales, from the atomic level to large macromolecules such as DNA, and even to cosmic phenomena like nebulae.^24–26^ Particularly, in DNA, chirality is transferred hierarchically from chiral sugar molecules, resulting in homochiral double-helical structures where hydrogen-bonding interactions play a vital role.^27^ Other noncovalent interactions, including coordination bonding, electrostatic interactions, and van der Waals forces, have also been utilized to construct supramolecular helical structures,^21,28^ with some showing enhanced magnetic transition dipole moments and *g*_lum_ values.^17,20,29,30^ However, helical structures synthesized from organic molecules are typically soft entities, making it challenging to understand structure–function relationships at a molecular level due to the lack of precise structural information.^26,31,32^ While helical structures assembled from metal clusters are generally crystalline aggregates, there are rarely any reports on their CPL behavior due to the lack of chirality.^33–40^ Therefore, we envision using chiral ligand-supported metal clusters to construct crystalline helices with precise structures and understand chirality transfer and amplification.

**Scheme 1:**
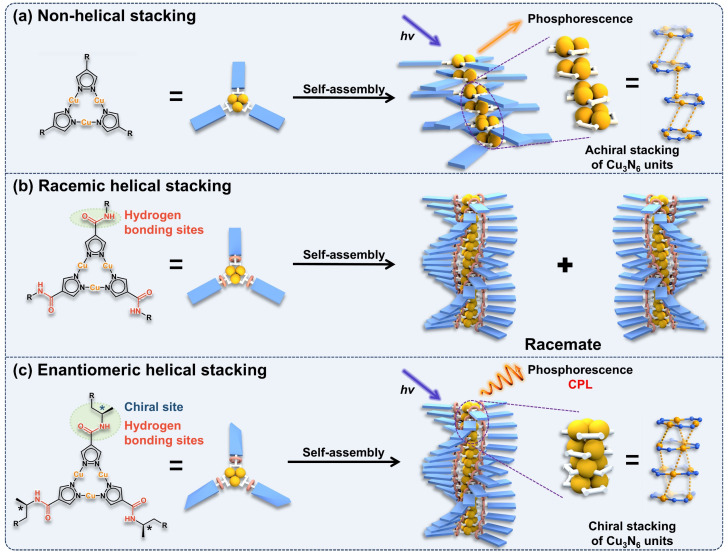
Schematic illustration of the design strategy for achieving CPL-active Cu(i) CTC aggregates. (a) Non-helical stacking of Cu(i) CTCs using pyrazolate ligands without hydrogen-bonding sites. (b) Racemic helical stacking of Cu(i) CTCs using pyrazolate ligands with hydrogen-bonding sites. (c) Enantiomeric helical stacking of Cu(i) CTCs using chiral pyrazolate ligands with hydrogen-bonding sites.

Cu(i) cyclic trinuclear complexes (CTCs) and their crystalline aggregates have been extensively studied and their bright phosphorescence was usually attributed to the intermolecular metal–metal-bond-dominated excited dimers (excimers).^41^ Driven by the intermolecular metallophilic interactions, simple Cu(i) CTCs tend to form aggregates through non-helical stacking of the metal clusters (Scheme 1a),^41^ resulting in the silence of CPL signals due to the lack of intrinsic chirality. Studies on the self-assembly of benzene-1,3,5-tricarboxamide derivatives show that helical stacking can be achieved through π⋯π stacking of the central phenyl rings and intermolecular hydrogen bonding of the peripheral amide groups.^42–44^ Inspired by this, we propose introducing hydrogen-bonding sites into the pyrazolate ligands of Cu(i) CTCs to promote intermolecular hydrogen bonding between adjacent Cu(i) CTC molecules. This may facilitate the formation of racemic helical-stacked aggregates (Scheme 1b).^45–49^ By further incorporating chiral groups into the pyrazolate ligands, it may be possible to afford homochiral helical-stacked aggregates with aligned luminescent and chiral centers, leading to direction-specific CPL emission (Scheme 1c).

**Fig. 1:**
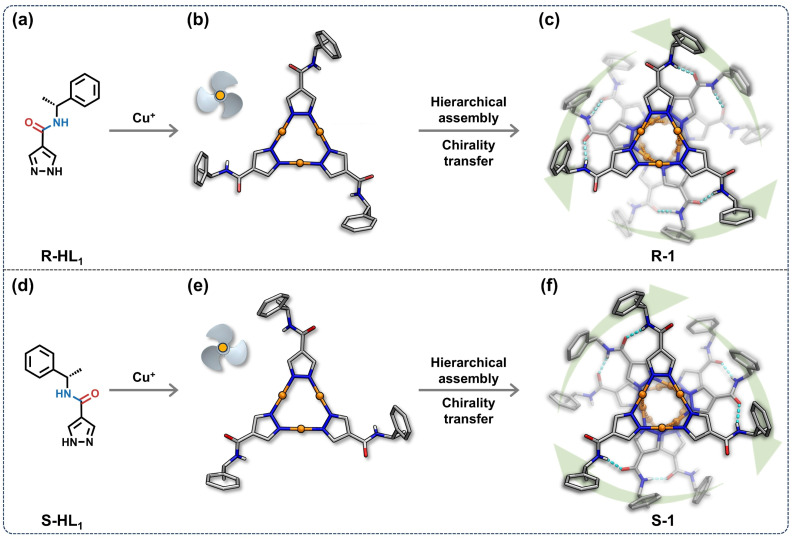
Self-assembly of R-1 (top) and S-1 (bottom). (a, d) Structures of R-HL_1_ or S-HL_1_. (b, e) *C*_3_-symmetry propeller-like conformations of the Cu(i) CTCs extracted from the crystal structures of R-1 and S-1. (c, f) Packing modes of R-1 or S-1 along the *a*-axis, with sky blue and orange dashed lines representing the hydrogen-bonding and metallophilic interactions, respectively. Colour codes: orange, Cu; grey, C; blue, N; red, O; white, H. For clarity, some H atoms have been omitted.

Herein, we present the successful construction of homochiral triple-helical supramolecular aggregates, denoted as R-1 and S-1, with enantiomeric acylamino-functionalized pyrazolate ligands and *in situ* formed Cu^+^ ions (Fig. 1). Structure analysis and theoretical calculations reveal that the metallophilic and hydrogen-bonding interactions are crucial for forming these helical stacking structures. R-1 and S-1 emit bright orange-red phosphorescence centered around 700 nm, showcasing excellent CPL signals with *g*_lum_ values of up to ±1 × 10^−2^, respectively. Photophysical measurements and computational simulations confirm that the exceptional *g*_lum_ values arise from the significant overlap of their chiral and luminescent centers at the core of the helical-stacked structures. Installing naphthyl chromophore in the pyrazolate ligands affords another set of triple-helical structures, denoted as R-2 and S-2, with unique dual CPL emission^50,51^ and anti-counterfeiting applications. Specifically, a low-energy phosphorescence arises from metal–metal-bond-dominated excimers and a high-energy ligand-centered fluorescence from the naphthyl group. This work describes an innovative approach to constructing triple-helical structures by integrating hydrogen-bonding sites into the peripheral chiral ligands of Cu(i) CTCs, enabling efficient chirality transfer and controlled CPL signals.

## Results and discussion

### Synthesis and characterization

The chiral pyrazole molecules (R-HL_1_ and S-HL_1_, Fig. 1a and d) were synthesized through amidation reactions (Scheme S1, see SI for details). Solvothermal reactions of R-HL_1_ or S-HL_1_ with Cu(NO_3_)_2_·3H_2_O in ethanol/pyridine at 140 °C for 72 hours afford colourless needle-shaped crystals of R-1 and S-1, respectively (Scheme S2). Their structures were elucidated by single-crystal X-ray diffraction (SCXRD) analysis, and phase purity was confirmed by powder X-ray diffraction (PXRD) analysis (Fig. S19). Their chemical compositions were verified through ^1^H/^13^C NMR (Fig. S9–S12) and elemental analysis. Thermogravimetric analysis (TGA) indicates that R-1 and S-1 are stable up to approximately 300 °C in an N_2_ atmosphere (Fig. S22a).

### Structural analyses

**Fig. 2:**
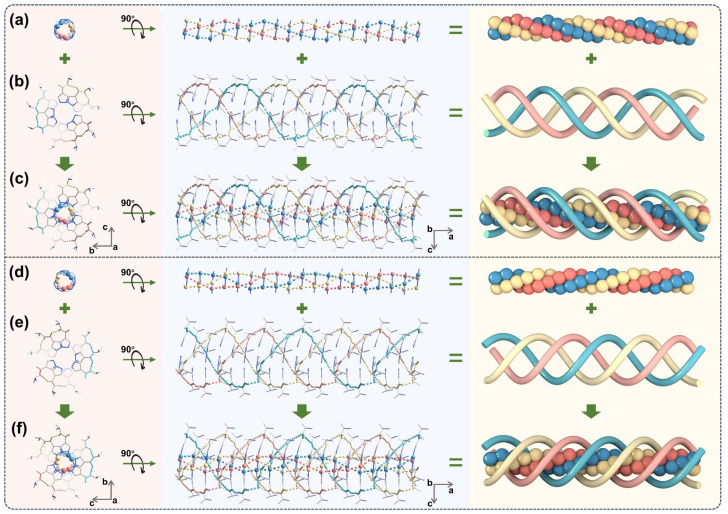
P-Type helical assembly in R-1 (top) and M-type helical assembly in S-1 (bottom). (a, d) Triple-helical metallophilic networks of Cu_3_N_6_ units. (b, e) Triple-helical hydrogen-bonding networks of peripheral ligands. (c, f) Overall triple-helical structures of R-1 and S-1. Each helical chain was depicted in one colour to highlight the triple helix. Some H atoms and all phenyl rings were omitted for clarity.

SCXRD analyses reveal that R-1 and S-1 crystallize in the monoclinic chiral space group *P*2_1_, with Flack parameters of 0.05(4) and 0.10(7), respectively (Table S3), indicating high reliability of the absolute configuration determination.^52^ As shown in Fig. 1 and 2, the structures of R-1 and S-1 are mirror images of each other. Thus, we take R-1 as an example for structural analysis. The asymmetric unit of R-1 contains three Cu(i) CTCs and two ethanol (EtOH) molecules, resulting in a molecular formula of [Cu_3_(R-L_1_)_3_]_3_·(EtOH)_2_ (Fig. S27a). Each Cu(i) CTC adopts a characteristic trinuclear structure, where three Cu^+^ ions are in linear coordination modes and bridged by three pyrazolate ligands, forming a nine-membered metallocycle (Cu_3_N_6_) (Fig. 1b). The Cu–N bond lengths vary from 1.801 Å to 1.843 Å, and the N–Cu–N bond angles span from 174.1° to 178.2° (Table S4). In a single Cu(i) CTC, the dihedral angles between the pyrazolate and phenyl rings range from 65.26° to 76.09° (Fig. S28a), closely matching those in the free R-HL_1_ molecule (67.95°, Fig. S29). This indicates that the coordination of Cu^+^ ions does not significantly distort the ligand conformation. On account of the installation of chiral carbon centers, each Cu(i) CTC adopts a *C*_3_-symmetry propeller-like conformation in R-1 (Fig. 1b).

Stacking analysis of Cu(i) CTCs in R-1 reveals intermolecular Cu⋯Cu distances ranging from 3.195 Å to 3.297 Å (Fig. S30a). These values were shorter than the sum of the van der Waals radii of the two Cu^+^ ions (1.96 Å each),^53,54^ suggesting the presence of Cu⋯Cu interactions between adjacent Cu(i) CTCs. The green isosurfaces between intermolecular Cu^+^ ions in the independent gradient model based on Hirshfeld partition (IGMH) analysis^55^ further confirm such metallophilic interactions (Fig. S57). In the peripheral pyrazolate ligands, the dihedral angles between the amide groups and pyrazolate rings range from 20.29° to 42.41° (Fig. S28b), likely due to the compromise of conjugation and intermolecular hydrogen-bonding demands.^56^ Three sets of intermolecular hydrogen bonds are observed between two adjacent Cu(i) CTCs (Fig. S27a), with C=O and N–H of amide groups serving as hydrogen bond acceptors and donors, respectively. The distances between the O and H atoms vary from 1.955 Å to 2.079 Å (Fig. S27a), and the N–H⋯O angles range from 157.9° to 171.0°.^57^ IGMH analysis reveals blue and green isosurfaces between H and O atoms, further confirming the existence of hydrogen-bonding interactions (Fig. S57).

The metallophilic interactions between the Cu_3_N_6_ units and the hydrogen-bonding interactions between the peripheral chiral pyrazolate ligands lead to P- and M-type^58^ triple helical assemblies (R-1 and S-1) (Fig. 2). Zhu *et al.* reported a triple-helical stacking through the self-assembly of Au_6_Cu_6_(4-methoxybenzenethiolate)_12_.^37^ However, the helical aggregates are achiral due to the coexistence of left- and right-handed helical chains in the same crystal, possibly attributable to the use of achiral ligands. Similarly, Sun *et al.* reported a meso-helical structure constructed from nanoclusters Cu_18_H(PET)_14_(PPh_3_)_6_(isothiocyanate)_3_, with both P- and M-type helices in a single crystal due to the use of achiral ligands.^38^ In contrast, we demonstrate the assembly of triple-helical aggregates with discrete P- or M-type configurations using corresponding chiral ligands. Interestingly, some R-1 and S-1 crystals exhibit distinct chirality on the macroscopic scale, with helix-like morphology revealed by scanning electron microscopy (SEM) images (Fig. S40).

The inner and outer triple helices in the crystals of R-1 were further dissected. In each triple-helical chain, the twist angles between adjacent Cu(i) CTCs are approximately 40° (Fig. S33). Consequently, a full turn of the outer triple-helical hydrogen-bonding network involves nine amide groups from pyrazolate ligands and two hydroxyl groups from ethanol molecules (Fig. S34a). Meanwhile, the inner metallophilic network completes a turn with nine Cu^+^ ions per strand (Fig. S35a). The pitches of both the inner and outer triple-helix are approximately 24.3 Å (Fig. S35b). Adjacent helical chains form pseudo-hexagonal packing along the *a*-axis (Fig. S36a), with 2_1_ axes parallel to the *b-*axis (Fig. S37). These stacking patterns resemble natural collagen,^59^ in which the pitch length is approximately 10 Å.^60,61^ Moreover, multiple C–H⋯π interactions can be observed between neighboring helical chains, with hydrogen atoms from methyl groups or benzene rings interacting with adjacent phenyl rings. The distances of the C–H⋯π interactions range from 2.246 Å to 2.838 Å (Fig. S39), and these interactions may play a role in restricting the rotation of phenyl rings within the crystal lattice.^62^

### Photophysical studies

**Fig. 3:**
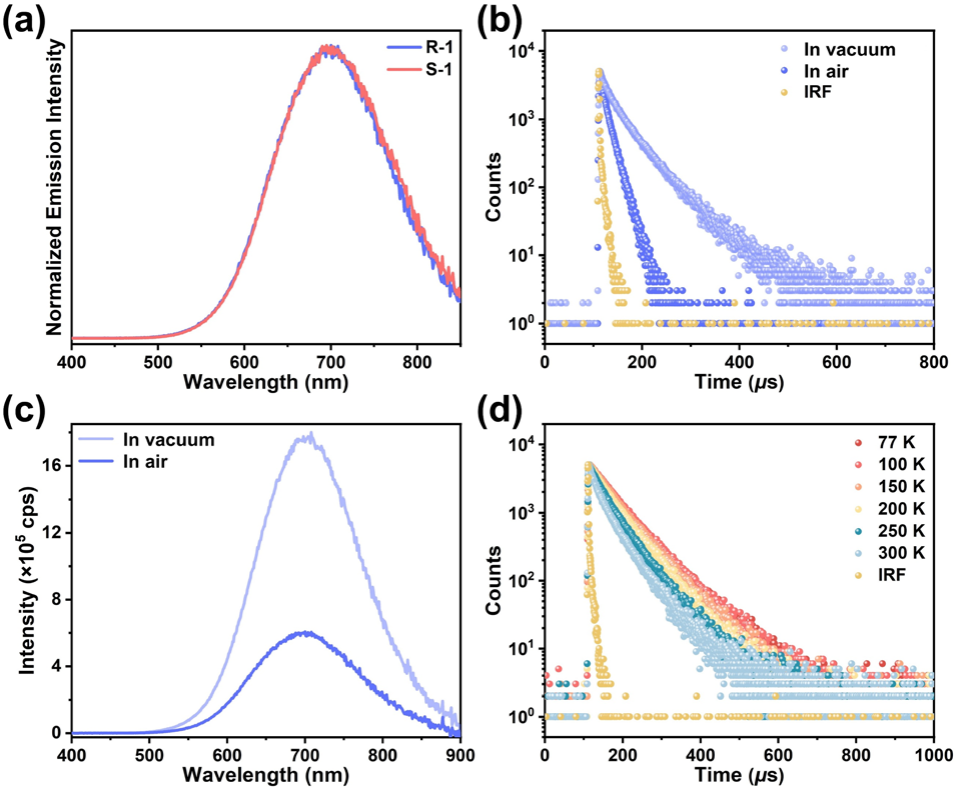
(a) Normalized emission spectra of R-1 and S-1 excited at 310 nm under ambient conditions. (b) Lifetime decay curves and (c) emission spectra (*λ*_ex_ = 310 nm) of R-1 in air and vacuum at room temperature. (d) Temperature-dependent lifetime decay curves of R-1 in vacuum. IRF: instrument response function.

The colourless crystals of R-1 and S-1 showed strong electronic absorption peaks near 270 nm (Fig. S44a). Upon excitation at 310 nm, both R-1 and S-1 exhibit broad orange-red emission bands peaking at about 700 nm (Fig. 3a), with average lifetimes in microseconds under ambient conditions (12.27 µs for R-1 and 14.23 µs for S-1, Fig. 3b, S46b, and Tables S5–S7). The results suggest that both R-1 and S-1 are phosphorescent emitters. Such emission behaviors are similar to traditional Cu(i) CTCs, indicating that the phosphorescence of R-1 and S-1 originates from their Cu–Cu-bonded excimers. Additionally, the emission intensity of R-1 and S-1 in vacuum are stronger than that in air (Fig. 3c and S46a), and the lifetimes of R-1 and S-1 in vacuum (41.04 µs for R-1 and 37.12 µs for S-1, Fig. 3b, S46b, and Tables S5–S7) are sustainably longer than those in air, the result suggest that oxygen could quench their emission, a phenomenon commonly observed in phosphorescence emission.^63^

Variable-temperature emission spectra of R-1 were collected, showing a broad emission band centered at 719 nm at 77 K, blue-shifting to 702 nm upon heating to 300 K (Fig. S48 and Table S6). The emission intensity at 77 K was approximately twice as large as that at 300 K, suggesting that low temperatures can effectively suppress thermally activated nonradiative transitions in R-1. The blue-shifting and emission quenching upon heating of R-1 are similar to traditional Cu(i) CTCs, and such a phenomenon was commonly attributed to lattice expansion and weakened intermolecular Cu⋯Cu interactions upon heating.^41^ The emission decay times for R-1 decrease from 63.82 µs at 77 K to 41.04 µs at 300 K in vacuum (Fig. 3d and Table S6), consistent with phosphorescence emission. Similar results were also observed for S-1 (Fig. S49 and Table S7).

**Fig. 4:**
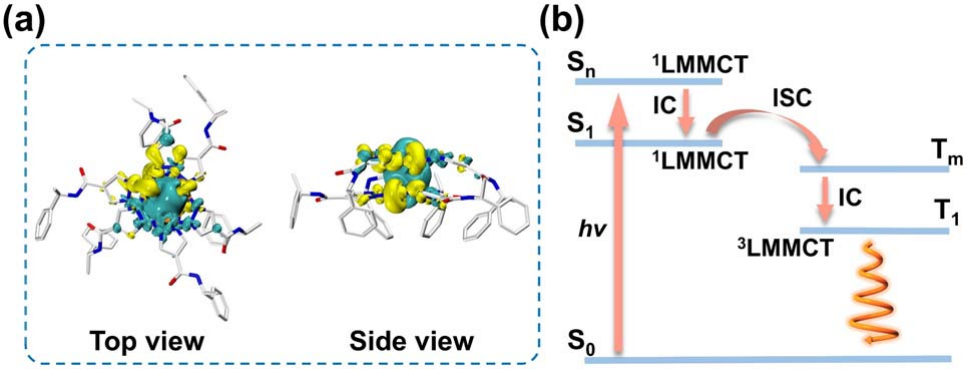
(a) EDD maps of the T_1_ state of R-1. The yellow colour indicates decreasing electron density in transition and the cyan colour indicates increasing electron density in transition. (b) The proposed photophysical process of R-1 at room temperature. LMMCT: ligand to metal−metal charge transfer, IC: internal conversion, ISC: intersystem crossing.

To further investigate the excitation and emission mechanism of R-1, density functional theory (DFT) and time-dependent density functional theory (TDDFT) calculations were performed (see SI for details). The initial dimer model was extracted from the crystal structure and optimized as the ground state (S_0_). As depicted in Fig. S58, the oscillator strength of the simulated UV-vis spectrum was similar to that of the experimental one. The main absorption band could be attributed to the transitions of S_0_ → S_6_ and S_0_ → S_7_, indicating the ligand-to-metal–metal charge transfer (^1^LMMCT) and minor ligand-centered electronic transition. According to Kasha's rule,^64^ the phosphorescence of the emitter originates from the lowest triplet state. Thus, the optimized dimer model (S_0_) was further used for geometrical optimization of the lowest-energy triplet excited state (T_1_). Interestingly, the shortest intermolecular Cu⋯Cu distances of the excimer were found to be 2.637 Å, significantly shorter than the ground state of 3.280 Å, suggesting enhanced intermolecular Cu⋯Cu interaction in the excited state (Fig. S59). Electron density difference (EDD) maps of T_1_ reveal that electron transfer primarily from localized orbitals of Cu^+^ ions and ligands to delocalized orbitals of intra/intermolecular Cu⋯Cu bonds upon excitation (Fig. 4a). Hence, the phosphorescence emission mechanism of R-1 can be attributed to the ^3^LMMCT, with the luminescent center predominantly at the Cu_3_N_6_ units. Upon excitation, high-lying S_6_ (*f* = 0.066) and S_7_ (*f* = 0.058) are efficiently populated, followed by internal conversion (IC) to the lowest singlet state S_1_ (^1^LMMCT/^1^LC, LC = ligand-centered) (Table S14). Subsequently, intersystem crossing (ISC) occurs from the ^1^LMMCT/^1^LC state (S_1_) to a triplet state at a similar energy level (T_m_), proceeding to T_1_*via* IC, and then giving out orange-red phosphorescence CPL emission (Fig. 4b).

### CD and CPL

**Fig. 5:**
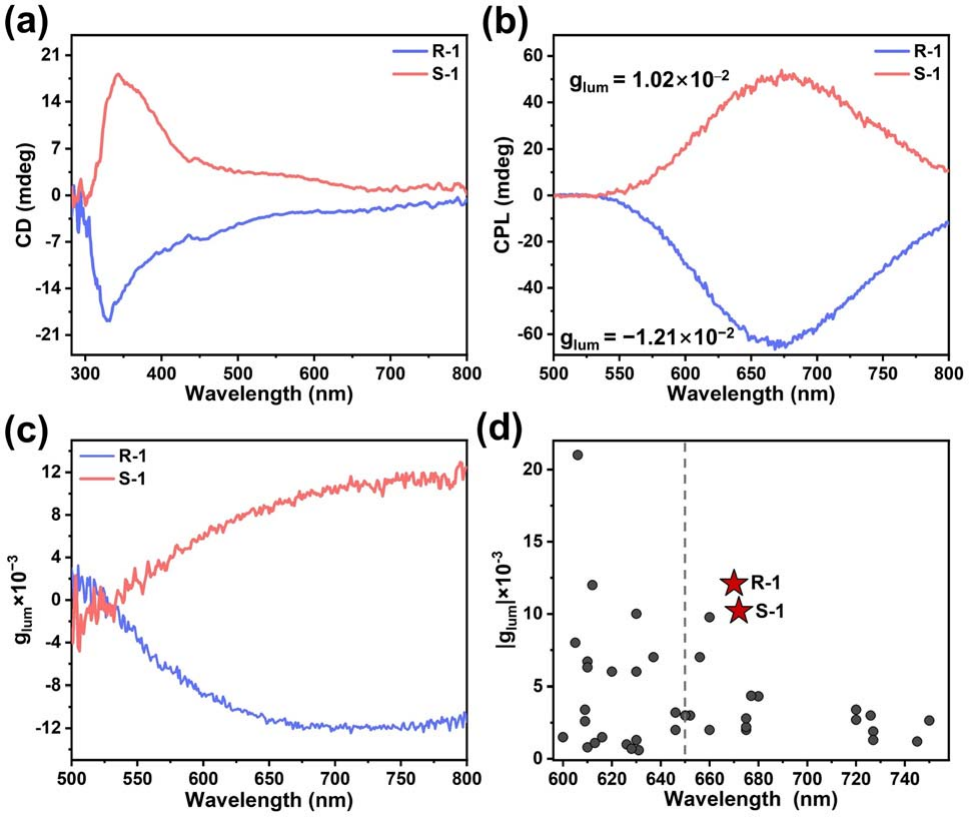
(a) CD spectra of R-1 and S-1 in solid state. (b) CPL spectra and (c) *g*_lum_ values of R-1 and S-1 in the crystal state (*λ*_ex_ = 305 nm), the monitoring DV values were set to 0.38 V. (d) Summary of the reported *g*_lum_ values of coinage metal complexes in solid state with emission wavelength exceeding 600 nm (see Table S18 for details).

Both R-1 and S-1 were found to crystallize in the chiral space group *P*2_1_, prompting us to investigate their chiroptical activities. As shown in Fig. 5a, circular dichroism (CD) spectra of R-1 and S-1 are nearly mirror images of each other, indicating opposite Cotton effects. In addition, the CD signals of R-1 and S-1 appeared around 330 nm (Fig. 5a), while R-HL_1_ and S-HL_1_ exhibited CD signals around 260 nm (Fig. S60b), suggesting chirality transfer from ligands to CTC molecules.^65^ The asymmetric absorbance factors (*g*_abs_) of R-1 and S-1 were determined to be −2.81 × 10^−4^ and 2.57 × 10^−4^, respectively.

R-1 and S-1 exhibit strong CPL signals in the wavelength of 500–800 nm (Fig. 5b), with *g*_lum_ values of −1.21 × 10^−2^ (*λ*_em_ = 676 nm) and +1.02 × 10^−2^ (*λ*_em_ = 673 nm), respectively (Fig. 5c). These *g*_lum_ values are the highest among the reported coinage metal complexes exhibiting red/near-infrared emission (*λ*_em_ > 650 nm) in the solid state (Fig. 5d and Table S18).^66,67^ The figure of merit (FM) values of R-1 and S-1 are modest, which can be attributed to partial quenching of their emission in air (Fig. S72 and Table S18). The exceptional CPL emission can be attributed to the overlap between the chiral center and luminescent center,^29,68–71^ both located at the triple-helical metallophilic networks. Additionally, hydrogen-bonding interactions between the peripheral ligands enhance structural rigidity, which may also contribute to the large *g*_lum_ values.^72^ To investigate the effect of helical structures on CPL signals, the crystals of R-1 and S-1 were ground, and the resulting solids showed reduced CPL signals (Fig. S62 and S63), consistent with their decreased crystallinity (Fig. S20). These results suggest the importance of helical packing in supramolecular aggregates to achieve large *g*_lum_ values.^73^

By replacing the phenyl with naphthyl in the pyrazolate ligands (Scheme S1), we successfully obtained another set of homochiral aggregates, R-2 and S-2 (Scheme S2), with PXRD patterns similar to R-1 and S-1 (Fig. S21). Theoretical simulations using the Materials Studio software package (see SI for details, Fig. S71, Tables S16 and S17) confirm the triple-helical structures of R-2 and S-2 (Fig. S68–S70), suggesting the generality of this self-assembly strategy. Interestingly, R-2 and S-2 exhibited dual-emission behaviors upon excitation at 370 nm (Fig. S51–S56). The high-energy emissions were centered at about 425 nm with lifetimes of approximately 2 ns, indicative of ligand-centered fluorescence (Fig. S43, S54a, and Table S5). The low-energy emissions were centered at about 680 nm with microsecond lifetimes, indicative of phosphorescence arising from metallophilic interactions (Fig. S54b, S55, S56b, Tables S5 and S8).^74,75^ Consequently, R-2 and S-2 displayed two sets of mirrored CPL signals (Fig. S67) with *g*_lum_ values both on the order of 10^−3^, realizing dual CPL emission in single-phased supramolecular aggregates. Such a dual emission enables multi-level encryptions, which may have significant implications in anti-counterfeiting applications (Fig. S73).^76,77^

## Conclusions

In summary, we have successfully demonstrated the self-assembly of homochiral Cu(i) CTC-based triple-helical aggregates using acylamino-functionalized chiral pyrazolate ligands. Structural and theoretical analyses have confirmed the significance of hydrogen-bonding and metallophilic interactions in triple-helical packing, leading to chirality transfer from ligands to aggregates. As such, the overlap of the chiral and luminescent centers enables exceptional CPL signals with large *g*_lum_ values. Furthermore, the incorporation of naphthyl chromophore into the pyrazolate ligands allows for assembling similar triple-helical structures that exhibit dual CPL signals, a phenomenon rarely observed in single-phased supramolecular aggregates. This work presents an innovative approach to creating metal clusters with well-defined helical stacking structures for direction-specific CPL emissions, advancing our understanding of helix formation in supramolecular aggregates, and aiding in the customized design of CPL materials for various applications.

## Author contributions

W. L., J. Z., X.-P. Z., and D. L. designed the research; G.-Q. H., H. Y., R.-Q. X., K. W., Y.-L. H. D.-B. H. and S.-B. L. conducted the experiments and data analysis; G.-Q. H. and H. Y. contributed to data analysis and theoretical calculation; G.-Q. H., H. Y., W. L., J. Z., X.-P. Z., and D. L. co-wrote the manuscript. All authors read and commented on the manuscript.

## Conflicts of interest

The authors declare no conflict of interest.

## Supplementary Material

SC-OLF-D5SC04965B-s001

SC-OLF-D5SC04965B-s002

## Data Availability

Supplementary information (SI) is available: includes full experimental details, synthesis protocols, characterization data, and computational details, *etc.* See DOI: https://doi.org/10.1039/d5sc04956b. CCDC 2378497 (R-HL_1_), 2378498 (S-HL_1_), 2378499 (R-HL_2_), 2378500 (S-HL_2_), 2378501 (R-1), and 2378502 (S-1) contain the supplementary crystallographic data for this paper.^78*a*–*f*^
