# Supplementary material for: Triple-helical aggregates of copper(i) cyclic trinuclear complexes for circularly polarized luminescence
Source: Chem Sci. 2025 Nov 25;17(5):2646–53. doi: 10.1039/d5sc04965b (PMC12687366; doi:10.1039/d5sc04965b)
Supplement: SC-017-D5SC04965B-s001 [file SC-017-D5SC04965B-s001.pdf]

Supplementary Information (SI) for Chemical Science.
This journal is © The Royal Society of Chemistry 2025

Supplementary Information (SI) for Chemical Science.
This journal is © The Royal Society of Chemistry 2025

Supplementary Information

Triple-Helical Aggregates of Copper(I) Cyclic Trinuclear Complexes for Circularly Polarized Luminescence

Guo-Quan Huang,[#a] Hu Yang,[#a] Ri-Qin Xia,[a] Kun Wu,[a] Yong-Liang Huang,[b] De-Bo Hao,[a] Shun-Bo Li,[a] Weigang Lu,*[a] Ji Zheng,*[a] Xiao-Ping Zhou*[a] and Dan Li*[a]

[a]College of Chemistry and Materials Science, Guangdong Provincial Key Laboratory of Supramolecular Coordination Chemistry, Jinan University, Guangzhou, Guangdong 510632, P. R. China

[b]Department of Chemistry, Shantou University Medical College, Shantou, Guangdong 515041, P. R. China.

[#]G.-Q. Huang and H. Yang contributed equally to this work.

Contents

1. General procedure

2. Experimental section

3. $^{1}H/^{13}C$ NMR spectra

4. Powder X-ray diffraction (PXRD) patterns

5. Thermal stabilities

6. Structural determination of R-HL_1/S-HL_1, R-HL_2/S-HL_2 and **R-1/S-1**

7. Scanning electron microscopy (SEM) images

8. Photophysical investigation

9. Computational details and results

10. Circular dichroism (CD) and circularly polarized luminescence (CPL)

11. Crystal structures modeling of **R-2/S-2**

12. Literature survey

13. Application showcase

14. References

1. General procedure

Infrared spectra were recorded using KBr disks on a Thermo Scientific Fourier transform infrared spectroscopy (FT-IR) Nicolet iS10 spectrometer, covering the range of 4000~400 cm^{-1}, the abbreviations used for the IR bands are: w = weak, m = medium, b = broad, s = strong, vs = very strong. ^{1}H and ^{13}C NMR spectra were obtained by using a Bruker Biospin Advance spectrometer (400 MHz). Elemental analyses were carried out with an Elementar vario MICRO CUBE equipment. Thermogravimetric analysis (TGA) curves were recorded by using TGA Q50 V20.6 with a heating rate of 10 °C/min from 40 to 800 °C in a nitrogen atmosphere. Powder X-ray diffraction (PXRD) experiments were performed on an Rigaku miniflex600 (Cu Kα, λ = 1.5418 Å) in the step of 0.02° under the conditions 40 kV and 40 mA. Scanning electron microscopy (SEM) analyses were performed on a COXEM EM 30AX PLUS microscope. Photoluminescence spectra and emission decay times at room temperature were acquired using the FLS-1000, while temperature-dependent photoluminescence spectra and emission decay times were acquired using the FLS-1000 equipped with an Oxford OptistatDN2 optical cryostat. Absolute quantum yields (QYs) were recorded by Hamamatsu C11347-11 absolute PL quantum yield spectrometer. Solid-state UV-vis absorption and circular dichroism (CD) spectra of **R-1/S-1**, **R-2/S-2** were thoroughly mixed with KBr through grinding and pressed into disks, and recorded by Bio-Logic MOS-500 multifunctional circular dichroism spectrometer. The circularly polarized luminescence (CPL) spectra of crystalline and non-crystalline samples were recorded by JASCO CPL-300 with scanning rate of 50 $nm\cdot min^{-1}$. The g_{lum} values were transferred from CPL spectra using the Spectra Manager software of JASCO.

2. Experimental section

All reagents and materials were obtained from Bidepharm, *J&K* Scientific and GHTECH *et al.*, and used as received without further purification.

2.1 Preparation of R-HL$_1$/S-HL$_1$ and R-HL$_2$/S-HL$_2$

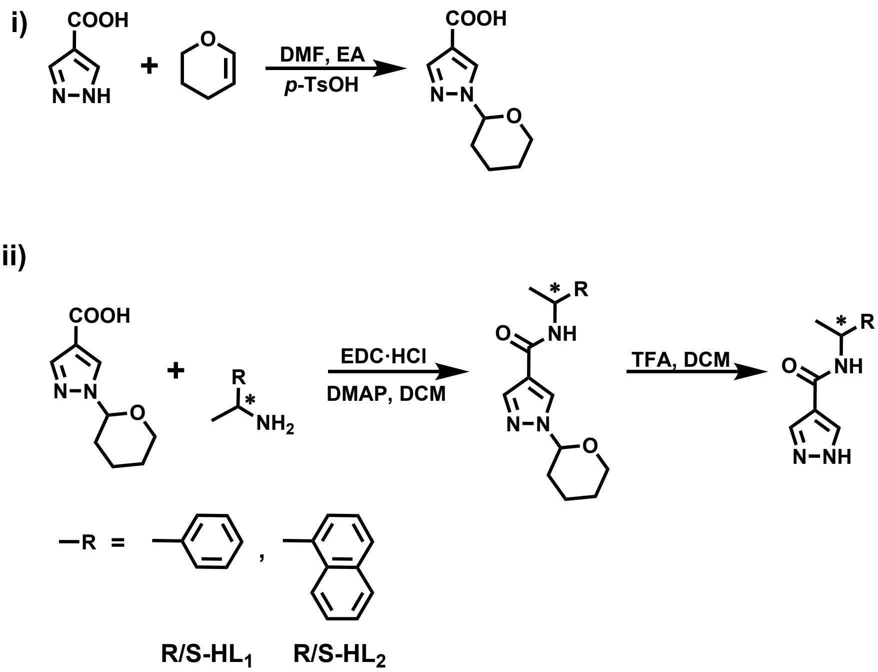

Scheme S1 The Synthesis of R-HL$_1$/S-HL$_1$ and R-HL$_2$/S-HL$_2$.

Synthesis of 1-(tetrahydro-2H-pyran-2-yl)-1H-pyrazole-4-carboxylic acid

This complex was synthesized according to the previously reported methods.[1]

Synthesis of (R/S)-*N*-(1-phenylethyl)-1*H*-pyrazole-4-carboxamide (R-HL$_1$/S-HL$_1$)

A solution of 1-(tetrahydro-2H-pyran-2-yl)-1H-pyrazole-4-carboxylic acid (10 mmol, 1.96 g) in dichloromethane (DCM) (200 mL) was prepared. To this, 4-dimethylaminopyridine (DMAP) (2 mmol, 0.25 g), 1-(3-dimethylaminopropyl)-3-ethylcarbodiimide hydrochloride (EDC·HCl) (25 mmol, 4.70 g), and (R/S)-1-phenylethan-1-amine (10 mmol, 1.3 mL) were added at room temperature. The reaction mixture was then refluxed for 12 hours, with progress monitored by TLC. Upon completion, 200 mL of water was added to the mixture. The solution was extracted with

DCM, and the organic layer was washed with brine (100 mL), dried over anhydrous sodium sulfate, and concentrated under reduced pressure using a rotary evaporator. The resulting yellow crude product was used directly in the next step without further purification.

The crude product obtained in the previous step was dissolved in 100 mL of DCM, followed by the addition of 20 mL of trifluoroacetic acid (TFA). The reaction mixture was stirred at room temperature for 24 hours. Upon completion, monitored by TLC, the solvent was removed under reduced pressure using a rotary evaporator, yielding a dark brown gel. The crude product was then purified by column chromatography, initially using a mixture of petroleum ether (PE) and ethyl acetate (EA) (PE:EA = 1:1), followed by pure EA, to yield R-HL_1/S-HL_1 as a white solid with a yield of 0.65 g (30%). The corresponding crystals were obtained by volatilization of methanol solution of R-HL_1/S-HL_1.

Synthesis of (R/S)-*N*-(1-(naphthalen-1-yl)ethyl)-1*H*-pyrazole-4-carboxamide (R-HL_2/S-HL_2)

The synthesis steps for R-HL_2/S-HL_2 were identical to those for R-HL_1/S-HL_1, with the exception that (R/S)-1-phenylethan-1-amine (10 mmol, 1.3 mL) was replaced by (R/S)-1-(naphthalen-1-yl)ethan-1-amine (10 mmol, 1.6 mL). R-HL_2/S-HL_2 was obtained as a colourless solid with a yield of 0.56 g (21%). The corresponding crystals were obtained by volatilization of methanol solution of R-HL_2/S-HL_2.

2.2. Preparation of R-1/S-1 and R-2/S-2

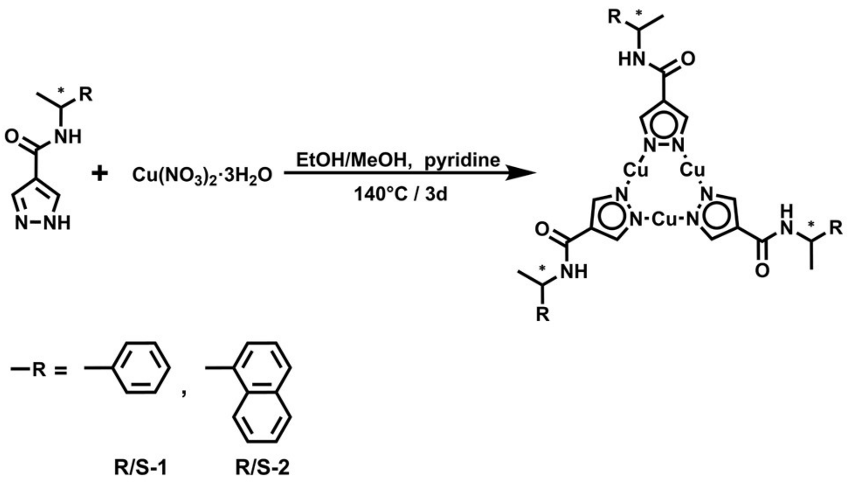

Scheme S2 The Synthesis of **R-1/S-1** and **R-2/S-2**.

Synthesis of R-1/S-1

After mixing $Cu(NO_3)_2·3H_2O$ (0.01 mmol, 2.4 mg), R-HL_1/S-HL_1 (0.015 mmol, 3.2 mg) and 10 μL pyridine in 2 mL of EtOH in 8-mm-inside-diameter Pyrex tube, the tube was sealed and then heated to 140 °C for 72 h in a programmable oven, followed by the slow cooling down to room temperature with the rate of 3 °C/h. Colourless needle crystals were collected and air-dried. Yield: 1.0 mg (35% based on $Cu(NO_3)_2·3H_2O$) for **R-1**, 0.9 mg (31% based on $Cu(NO_3)_2·3H_2O$) for **S-1**. Elemental analyses of **R-1** ($C_{36}H_{36}Cu_3N_9O_3$, %): calculated: C, 51.89; H, 4.67; N, 14.59; found: C, 51.50; H, 4.79; N, 14.94. Elemental analyses of **S-1** ($C_{36}H_{36}Cu_3N_9O_3$, %): calculated: C, 51.89; H, 4.67; N, 14.59; found: C, 51.54; H, 4.62; N, 14.93. FT-IR data (KBr, cm^{-1}) of **R-1**: 553 (s), 603 (w), 630 (w), 698 (w), 762 (s), 841 (m), 865 (m), 913 (w), 1018 (m), 1054 (s), 1094 (w), 1109 (w), 1127 (w), 1184 (m), 1210 (m), 1253 (vs), 1375 (m), 1402 (m), 1446 (s), 1494 (s), 1557 (vs), 1633 (vs), 2870 (w), 2933 (w), 2973 (m), 3031 (m), 3060 (w), 3087 (w), 3114 (w), 3308 (b), 3420 (b). FT-IR data (KBr, cm^{-1}) of **S-1**: 552 (s), 603 (w), 629 (w), 700 (w), 749 (s), 841 (m), 864 (m), 913 (w), 1019 (m), 1052 (s), 1098 (w), 1126 (w), 1189 (w), 1207 (w), 1253 (vs), 1374 (w), 1399 (m), 1448 (s), 1492 (s), 1557 (vs), 1635 (vs), 2869 (w), 2926 (w), 2970 (m), 3031 (m), 3065 (w), 3085 (w), 3105 (w), 3269 (b), 3424 (b).

Synthesis of R-2/S-2

After mixing $Cu(NO_3)_2·3H_2O$ (0.01 mmol, 2.4 mg), R-HL_2/S-HL_2 (0.015 mmol, 4.0 mg) and 10 μL pyridine in 3 mL of MeOH in 8-mm-inside-diameter Pyrex tube, the tube was sealed and then heated to 140 °C for 72 h in a programmable oven, followed by the slow cooling down to room temperature with the rate of 3 °C/h. Colourless needle crystals were collected and air-dried. Yield: 1.6 mg (48% based on $Cu(NO_3)_2·3H_2O$) for **R-2**, 1.8 mg (54% based on $Cu(NO_3)_2·3H_2O$) for **S-2**. Elemental analyses for **R-2** ($C_{48}H_{42}Cu_3N_9O_3$, %): calculated: C, 58.62; H, 4.30; N, 12.82; found: C, 57.60; H, 4.81; N, 12.94. Elemental analyses for **S-2** ($C_{48}H_{42}Cu_3N_9O_3$, %): calculated: C, 58.62; H, 4.30; N, 12.82; found: C, 58.53; H, 4.23; N, 12.81. FT-IR data (KBr, cm^{-1}) of **R-2**: 439 (w), 467 (w), 509 (w), 554 (w), 575 (w), 613 (w), 636 (m), 646 (w), 651 (w), 675 (w), 707 (m), 717 (w), 733 (m), 739 (m), 774 (vs), 803 (s), 825 (w), 865 (w), 912 (w), 985 (w), 1014 (w), 1054 (s), 1089 (w), 1123 (w), 1174 (w), 1192 (w), 1249 (vs), 1334 (w), 1380 (w), 1403 (m), 1443 (m), 1512 (s), 1558 (vs), 1632 (vs), 2867 (w), 2936 (w), 2976 (w), 3056 (m), 3113 (w), 3307 (b), 3433 (b), 3622 (m), 3640 (w). FT-IR data (KBr, cm^{-1}) of **S-2**: 424 (w), 449 (w), 474 (w), 509 (w), 545 (w), 576 (w), 607 (w), 637 (m), 667 (w), 703 (m), 739 (w), 776 (vs), 806 (s), 824 (m), 861 (m), 914 (w), 995 (w), 1018 (w), 1061 (s), 1085 (w), 1116 (w), 1170 (w), 1200 (m), 1243 (vs), 1333 (w), 1376 (w), 1400 (m), 1454 (m), 1515 (s), 1558 (vs), 1631 (vs), 2863 (w), 2934 (w), 2982 (w), 3055 (m), 3122 (w), 3322 (b), 3412 (b), 3630 (w).

Film fabrication

PMMA (2.5 g) was dissolved in dichloromethane (50 mL) under magnetic stirring at room temperature until complete dissolution, yielding a PMMA matrix solution with a concentration of 50 mg/mL. Subsequently, 1 mL aliquots of the matrix solution were transferred into centrifuge tubes, followed by the addition of crystalline powders of **S-1**/**R-2**/**S-2** (0.5 mg) into individual aliquots to fabricate flexible polymer films via solvent evaporation, resulting in **S-1**@PMMA, **R-2**@PMMA, and **S-2**@PMMA, respectively.

3. ^1H / ^{13}C NMR spectra

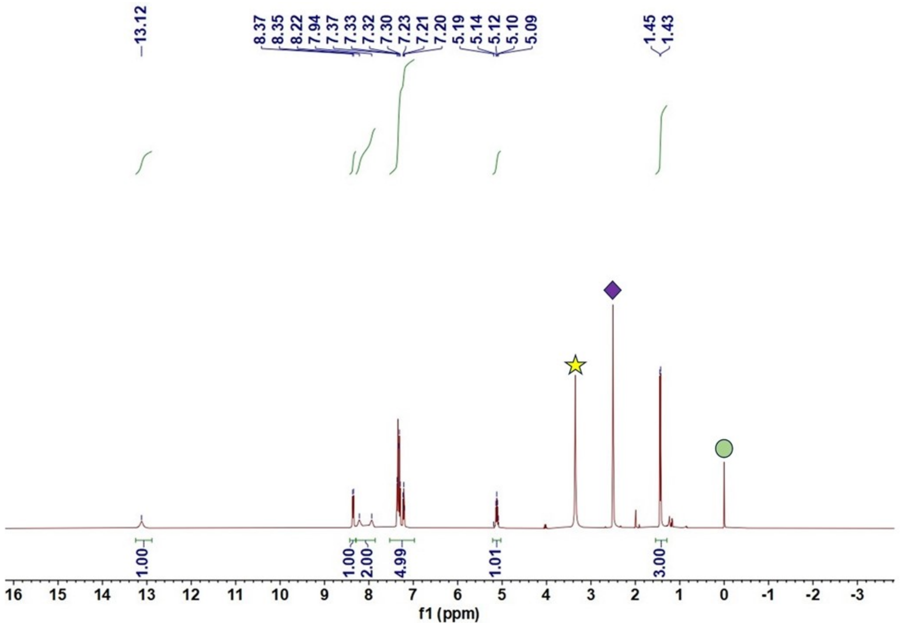

Fig. S1 ^1H NMR (400 MHz, DMSO–d_6) spectrum of R-HL_1. The star, square and circle shapes denote the solvent residual signals of H_2O, DMSO and TMS, respectively.

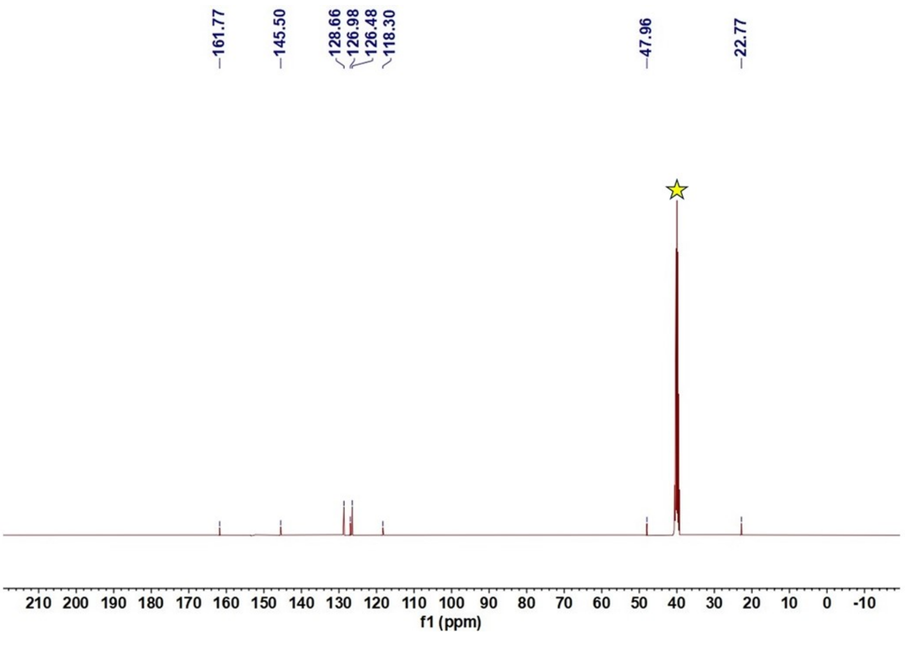

Fig. S2 ^{13}C NMR (100 MHz, DMSO–d_6) spectrum of R-HL_1. The star shape denotes the solvent residual signals of DMSO.

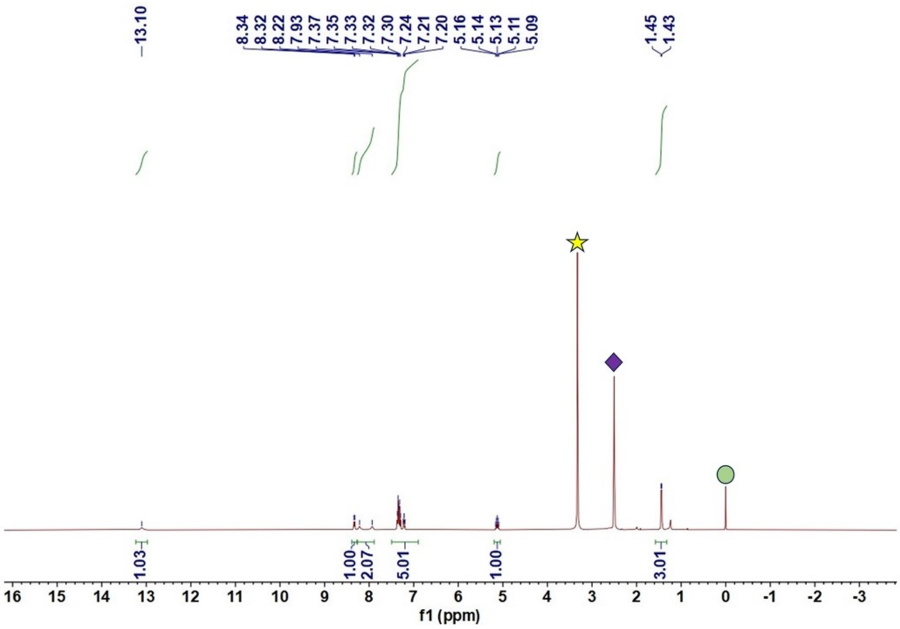

Fig. S3 ^{1}H NMR (400 MHz, DMSO–d_6) spectrum of S-HL_1. The star, square and circle shapes denote the solvent residual signals of H_2O, DMSO and TMS, respectively.

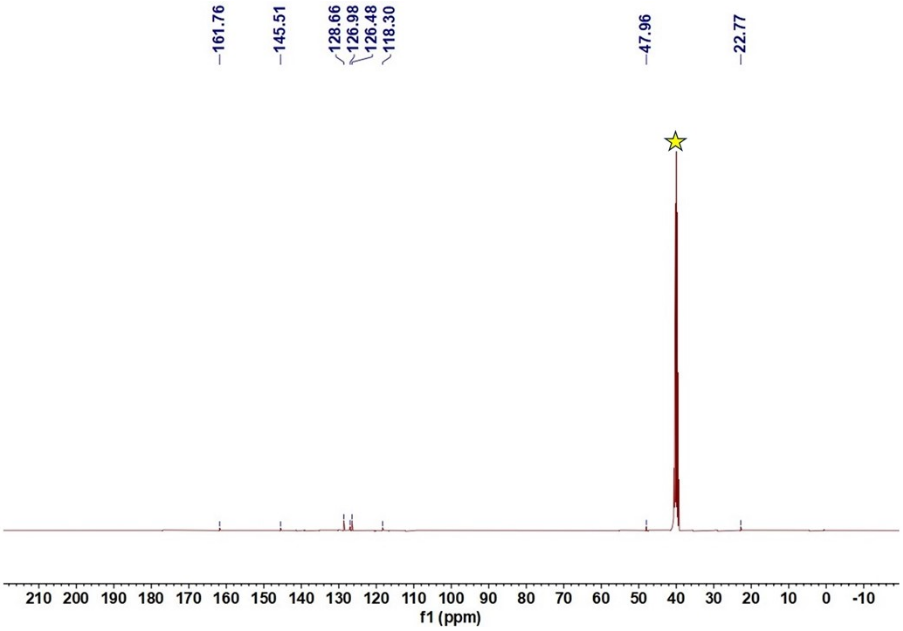

Fig. S4 ^{13}C NMR (100 MHz, DMSO–d_6) spectrum of S-HL_1. The star shape denotes the solvent residual signals of DMSO.

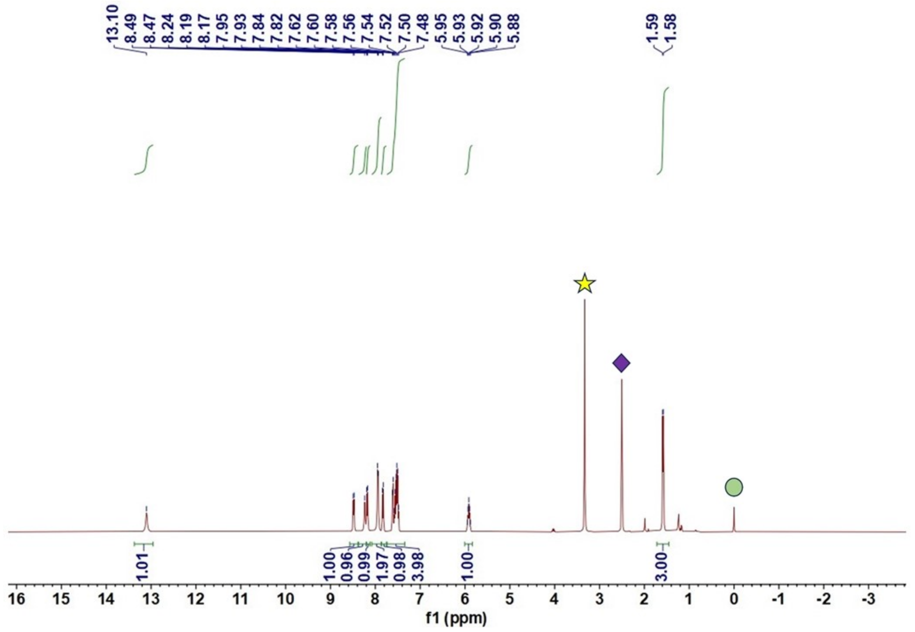

Fig. S5 ^1H NMR (400 MHz, DMSO–d_6) spectrum of R-HL_2. The star, square and circle shapes denote the solvent residual signals of H_2O, DMSO and TMS, respectively.

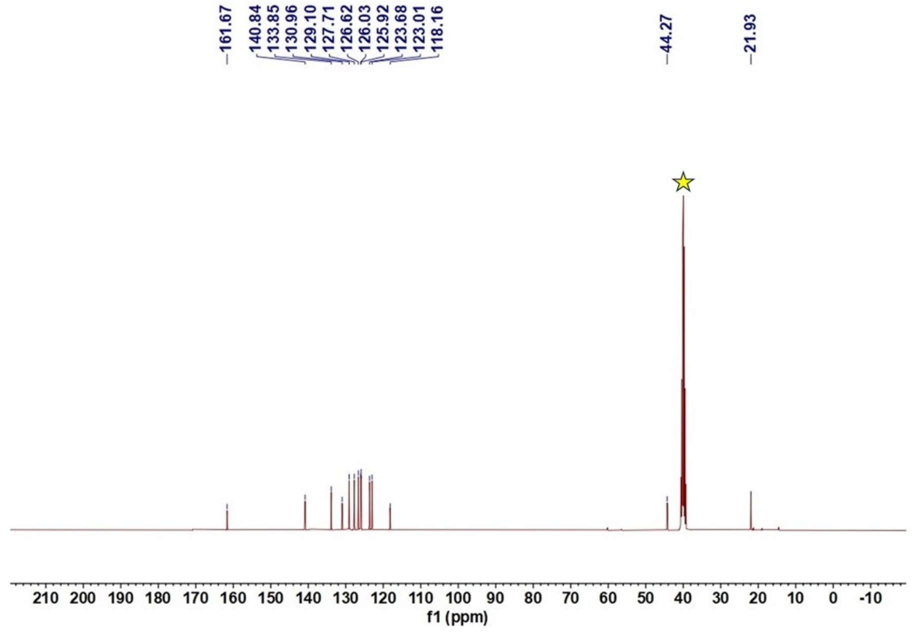

Fig. S6 ^{13}C NMR (100 MHz, DMSO–d_6) spectrum of R-HL_2. The star shape denotes the solvent residual signals of DMSO.

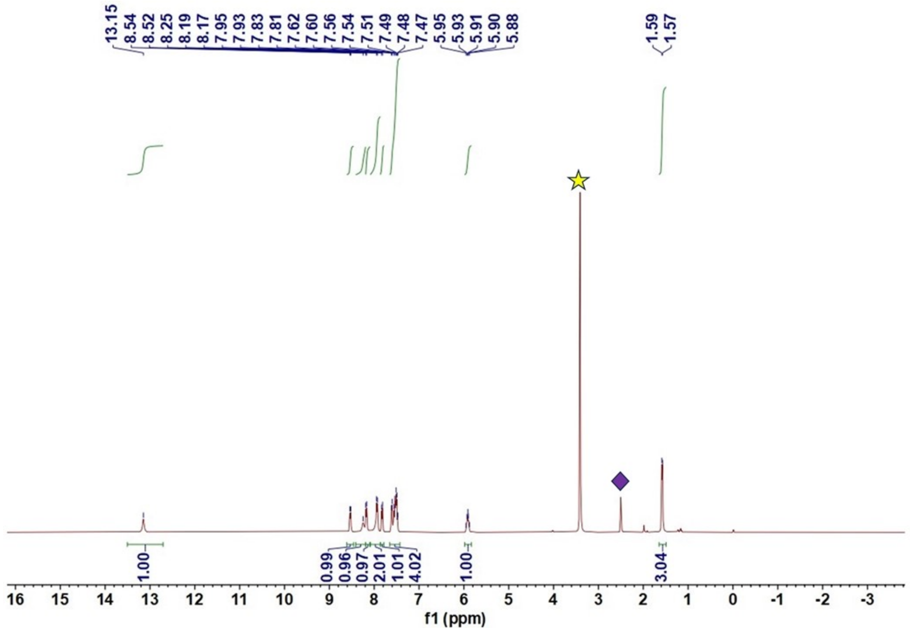

Fig. S7 ^{1}H NMR (400 MHz, DMSO–d_6) spectrum of S-HL_2. The star and square shapes denote the solvent residual signals of H_2O and DMSO, respectively.

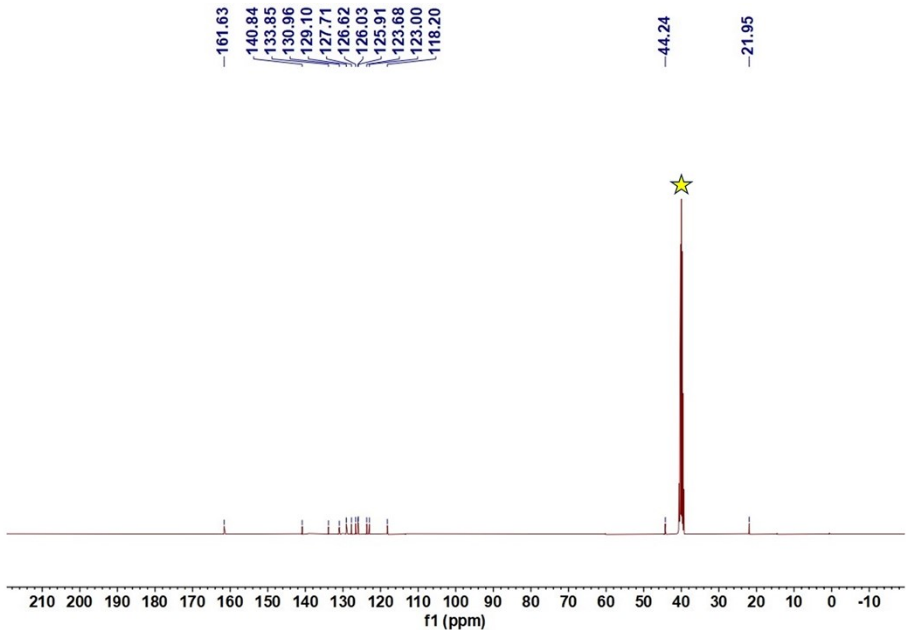

Fig. S8 ^{13}C NMR (100 MHz, DMSO–d_6) spectrum of S-HL_2. The star shape denotes the solvent residual signals of DMSO.

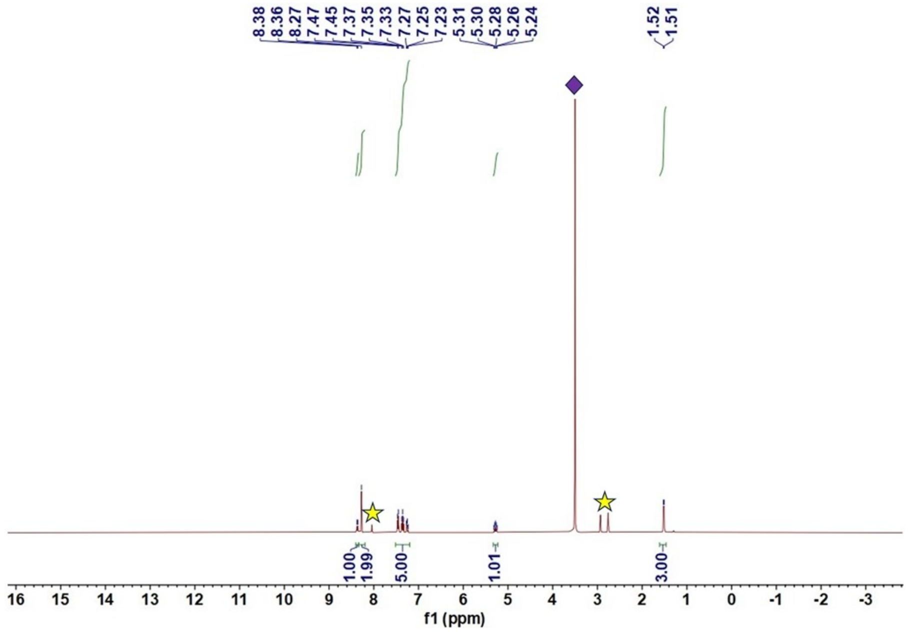

Fig. S9 ^{1}H NMR (400 MHz, DMF–d$_7$) spectrum of **R-1**. The star and square shapes denote the solvent residual signals of DMF and H_2O, respectively.

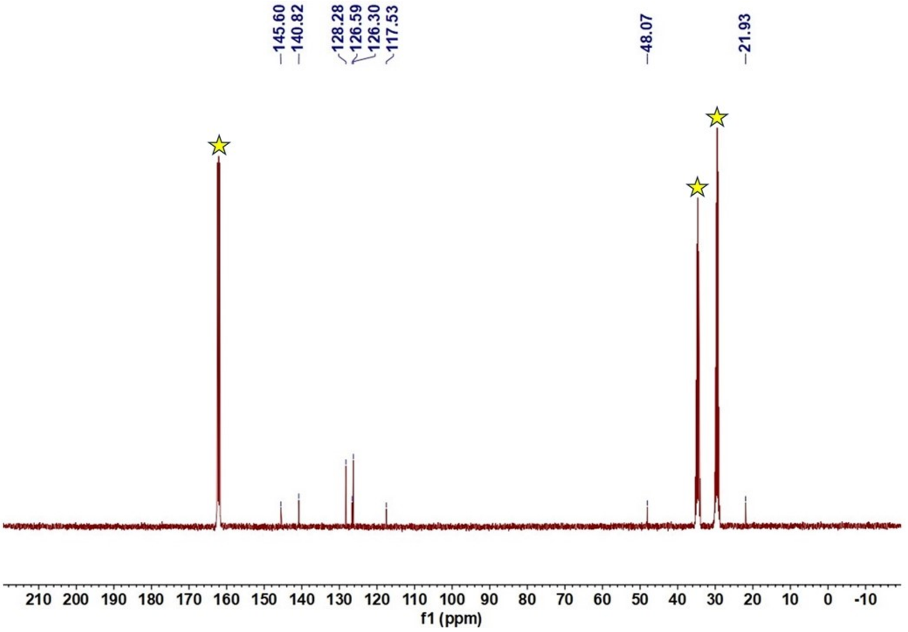

Fig. S10 ^{13}C NMR (100 MHz, DMF–d$_7$) spectrum of **R-1**. The star shape denotes the solvent residual signals of DMF.

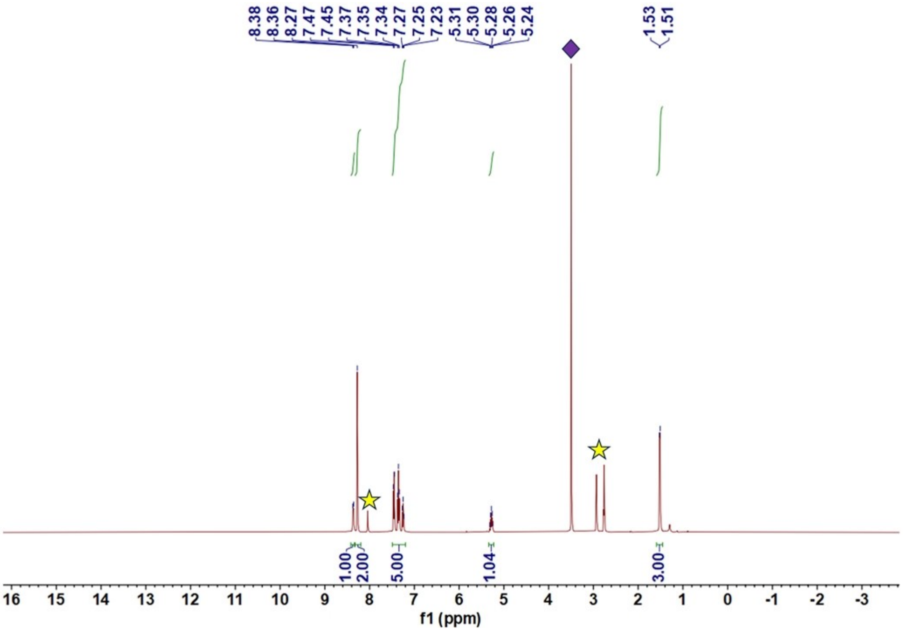

Fig. S11 ^{1}H NMR (400 MHz, DMF–d_7) spectrum of **S-1**. The star and square shapes denote the solvent residual signals of DMF and H_2O, respectively.

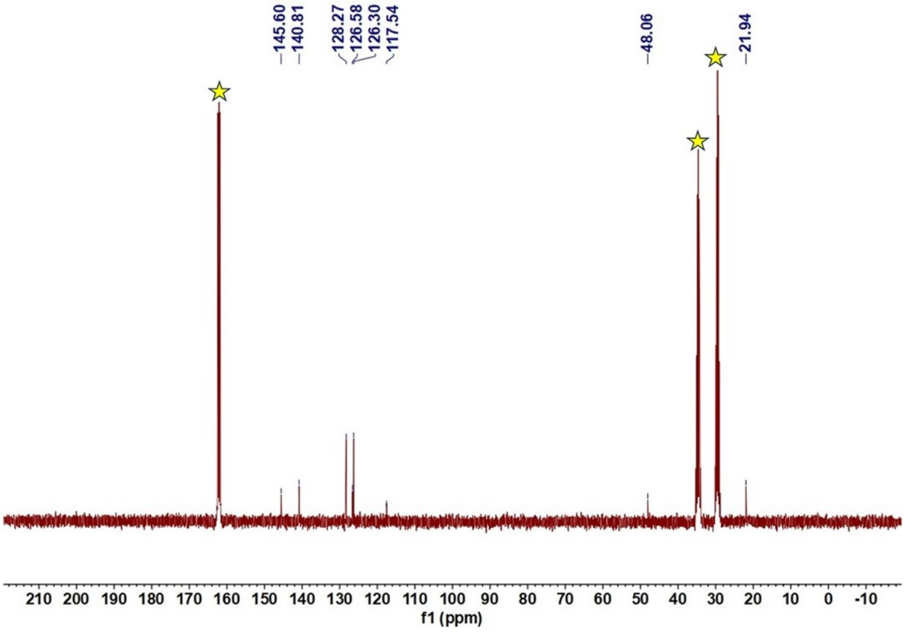

Fig. S12 ^{13}C NMR (100 MHz, DMF–d_7) spectrum of **S-1**. The star shape denotes the solvent residual signals of DMF.

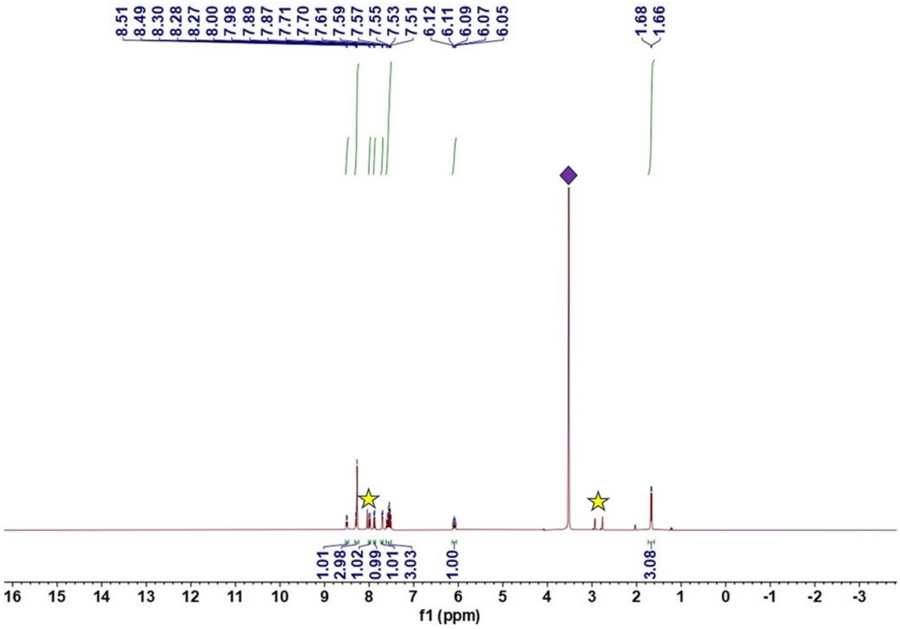

Fig. S13 ^{1}H NMR (400 MHz, DMF–d_7) spectrum of **R-2**. The star and square shapes denote the solvent residual signals of DMF and H_2O, respectively.

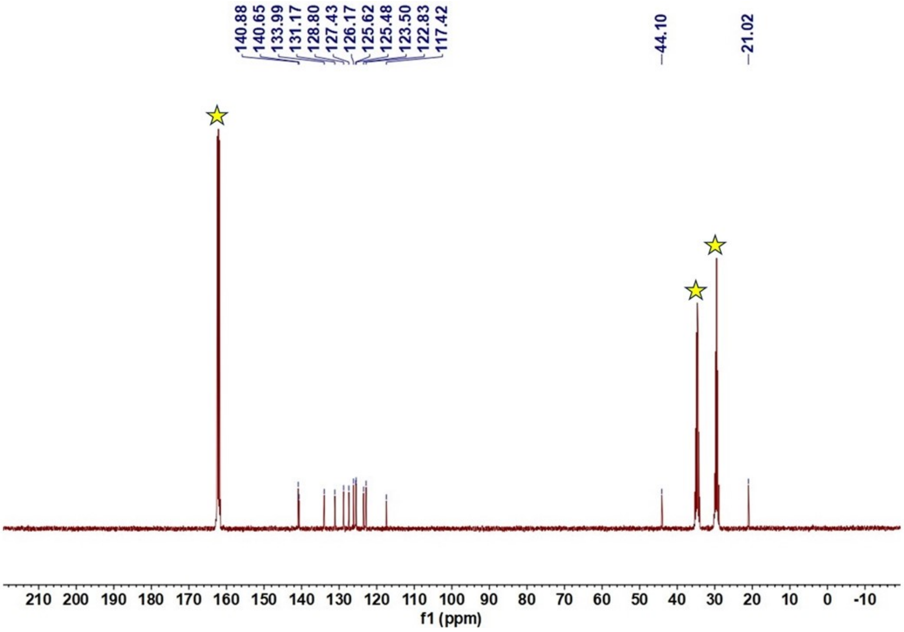

Fig. S14 ^{13}C NMR (100 MHz, DMF–d_7) spectrum of **R-2**. The star shape denotes the solvent residual signals of DMF.

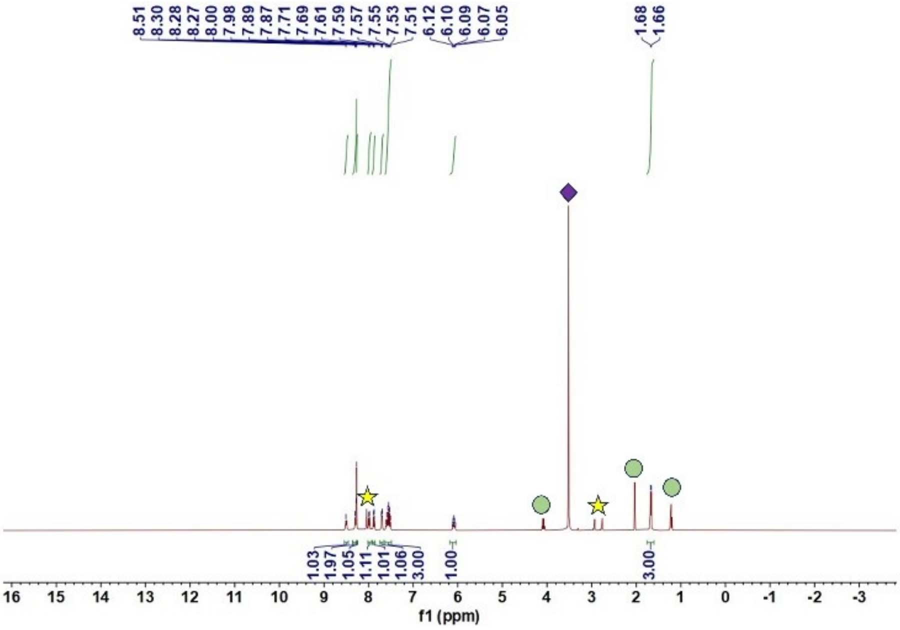

Fig. S15 ^{1}H NMR (400 MHz, DMF–d_7) spectrum of **S-2**. The star, circle and square shapes denote the solvent residual signals of DMF, ethyl acetate and H_2O, respectively.

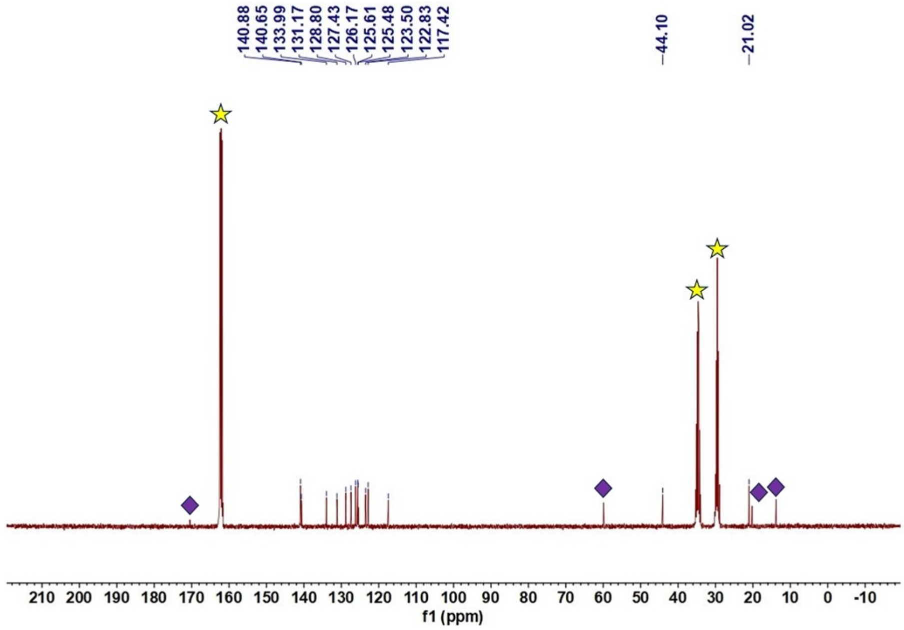

Fig. S16 ^{13}C NMR (100 MHz, DMF–d_7) spectrum of **S-2**. The square and star shapes denote the solvent residual signals of ethyl acetate and DMF, respectively.

4. PXRD patterns

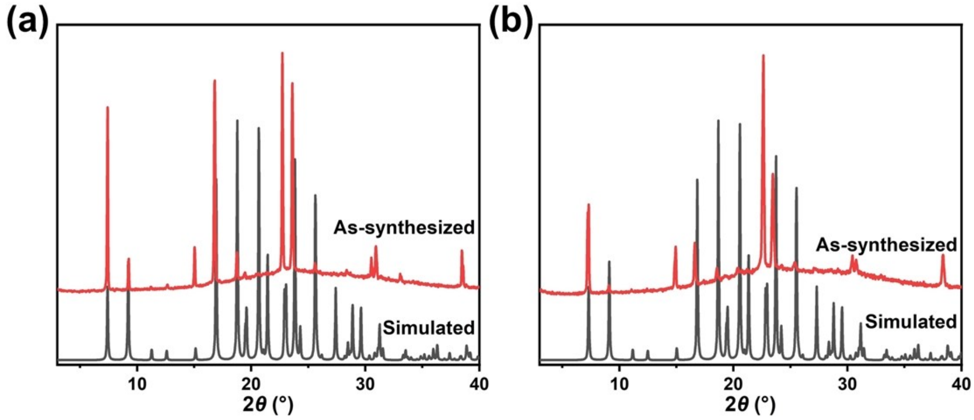

Fig. S17 PXRD patterns of crystalline (a) R-HL_1 and (b) S-HL_1.

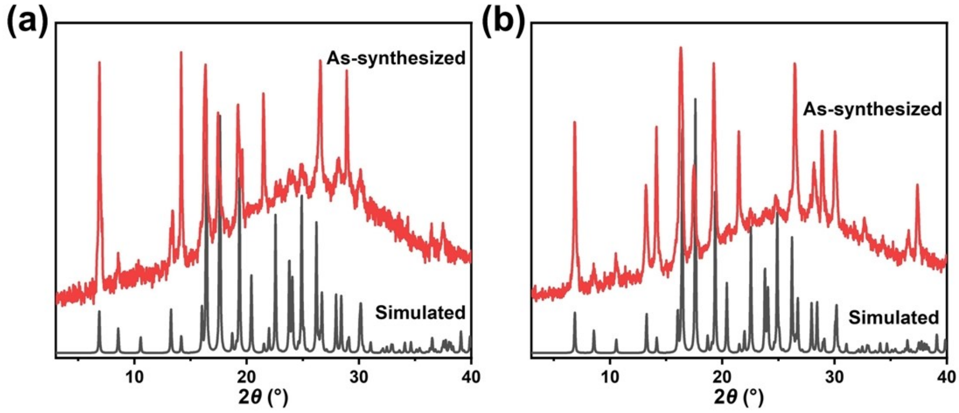

Fig. S18 PXRD patterns of crystalline (a) R-HL_2 and (b) S-HL_2.

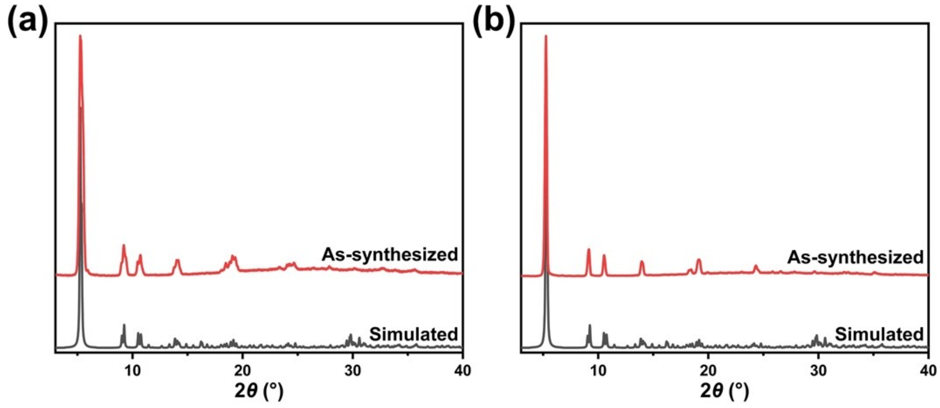

Fig. S19 PXRD patterns of (a) **R-1** and (b) **S-1**.

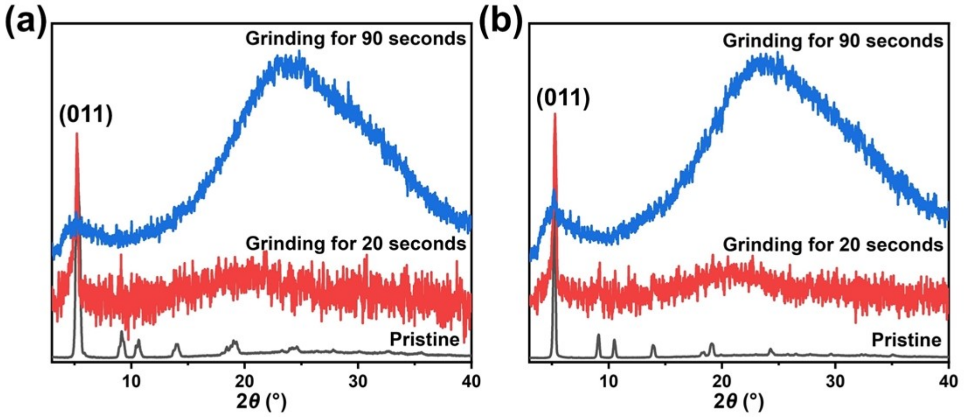

Fig. S20 PXRD patterns of (a) **R-1** and (b) **S-1** before grinding, after grinding for 20 seconds (red line) with low-crystallinity and further grinding for 70 seconds (bule line) with non-crystallinity. The low-crystallinity and non-crystallinity of **R-1**/**S-1** in powder state suggest the possible decomposition of their helical structures along the *a*-axis.

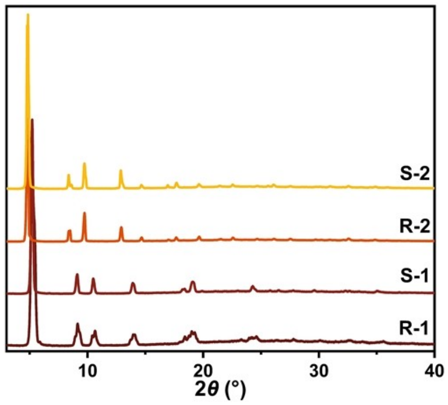

Fig. S21 PXRD patterns of **R-1**/**S-1** and **R-2**/**S-2**.

5. Thermal stabilities

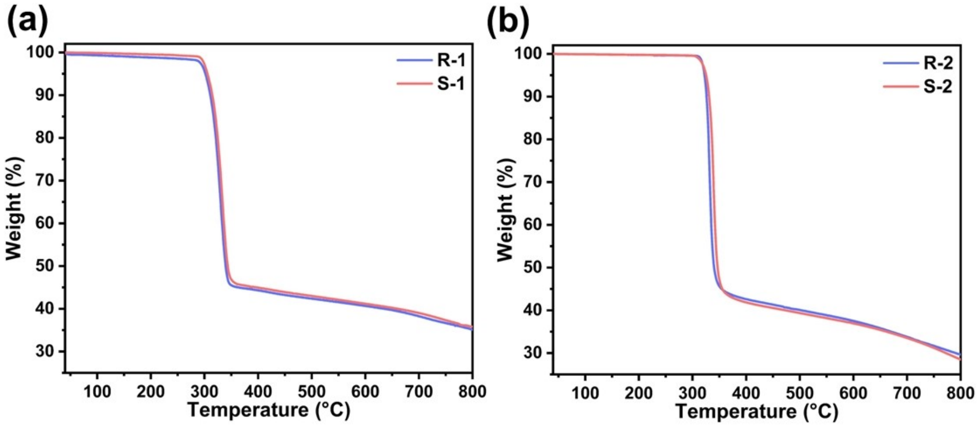

Fig. S22 TGA curves of (a) **R-1**/**S-1** and (b) **R-2**/**S-2**.

6. Structural Determination

Single crystal structures of R-HL_1/S-HL_1, R-HL_2/S-HL_2 and **R-1/S-1** were determined by single-crystal X-ray diffraction (SCXRD) at 100 K using an XtaLAB PRO MM007-DW diffractometer system, equipped with an RA-Micro7HF-MR-DW(Cu/Mo) X-ray generator and HyPix-6000HE Hybrid Photon Counting (HPC) X-ray detector (Rigaku, Japan, Cu Kα, graphite monochromator, λ = 1.54 Å). The structures were solved by direct methods and refined by full-matrix least-squares refinements based on F^2. Anisotropic thermal parameters were applied to all non-hydrogen atoms, with hydrogen atoms placed in calculated positions and refined using isotropic thermal parameters, riding on their parent atoms. All calculations were performed using the SHELXTL system of computer programs.[2] Some reflections of all the complexes were omitted owing to poor agreement. Crystallographic data and structure refinement parameters are summarized in Tables S1–S3.

6.1 Structural Determination of R-HL$_1$/S-HL$_1$

Table S1 Crystallographic data and refinement parameters of R-HL$_1$/S-HL$_1$.

Item	R-HL$_1$	S-HL$_1$
CCDC number	2378497	2378498
Temperature (K)	100.01(16)	100.00(10)
Empirical formula	$C_{12}H_{13}N_3O$	$C_{12}H_{13}N_3O$
Formula weight	215.25	215.25
Crystal system	monoclinic	monoclinic
Space group	$P2_1$	$P2_1$
a (Å)	9.3639(3)	9.3663(2)
b (Å)	5.08840(10)	5.08920(10)
c (Å)	11.5646(3)	11.5614(2)
β (°)	96.300(3)	96.3050(10)
Volume (Å^3)	547.69(3)	547.764(19)
Z	2	2
ρ_{calc} (g/cm^3)	1.305	1.305
F(000)	228.0	228.0
2θ range for data collection (°)	7.692 to 156.906	7.694 to 156.408
Index ranges	$-11 \le h \le 11$, $-6 \le k \le 6$, $-14 \le l \le 14$	$-11 \le h \le 11$, $-6 \le k \le 6$, $-14 \le l \le 14$
Reflections collected	9080	10392
Independent reflections	2284 [R_{int} = 0.0430, R_{sigma} = 0.0336]	2278 [R_{int} = 0.0323, R_{sigma} = 0.0253]
Data/restraints/ parameters	2284/1/146	2278/1/146
Goodness-of-fit on F^2	1.145	1.112
Completeness (%)	99.9	99.2
^{a}Final R indexes [I>=2σ (I)]	R_1 = 0.0356	R_1 = 0.0427
^{b}Final R indexes [all data]	wR_2 = 0.1011	wR_2 = 0.0943
Largest diff. peak/hole (e Å^{-3})	0.15/−0.22	0.19/−0.19
Flack parameter	−0.15(18)	0.08(14)

$^aR_1 = \sum||F_o|-|F_c||/\sum|F_o|$. $^bwR_2 = \{[\sum w(F_o^2-F_c^2)^2]/\sum[w(F_o^2)]\}^{1/2}$; $w = 1/[\sigma^2(F_o^2)+(aP)^2+bP]$, where $P = [\max(F_o^2, 0) + 2F_c^2]/3$ for all data.

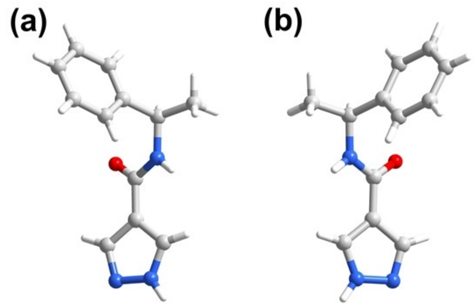

Fig. S23 Molecular structures of (a) R-HL_1 and (b) S-HL_1. Colour codes: grey, C; blue, N; red, O; white, H.

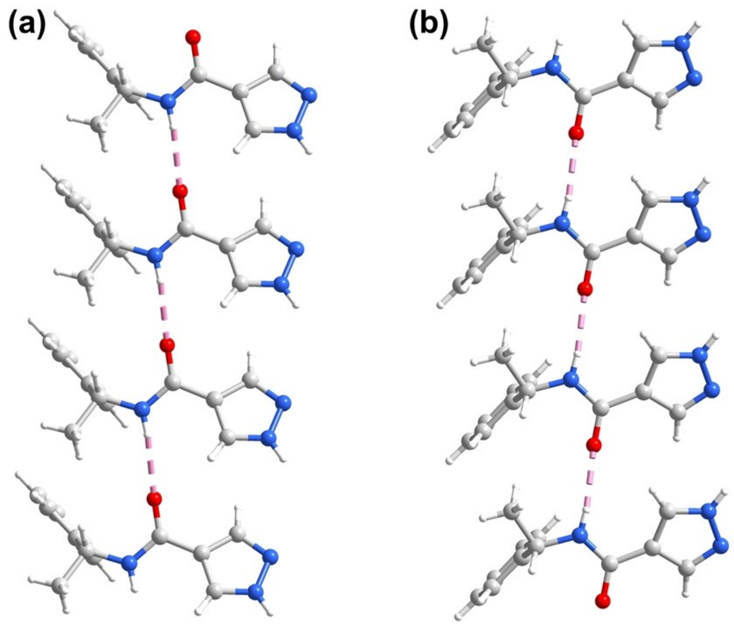

Fig. S24 Packing modes of (a) R-HL_1 and (b) S-HL_1. Dashed lines showing hydrogen-bonding interactions between the adjacent molecules. Colour codes: grey, C; blue, N; red, O; white, H.

6.2 Structural Determination of R-HL_2/S-HL_2

Table S2 Crystallographic data and refinement parameters of R-HL_2/S-HL_2.

Item	R-HL_2	S-HL_2
CCDC number	2378499	2378500
Temperature (K)	99.99(10)	99.98(10)
Empirical formula	$C_{16}H_{15}N_3O$	$C_{16}H_{15}N_3O$
Formula weight	265.31	265.31
Crystal system	orthorhombic	orthorhombic
Space group	$P2_12_12_1$	$P2_12_12_1$
a (Å)	5.02106(10)	5.02560(10)
b (Å)	10.7938(2)	10.7811(4)
c (Å)	24.2999(6)	24.3162(7)
Volume (Å^3)	1316.96(5)	1317.49(7)
Z	4	4
ρ_{calc} (g/cm^3)	1.338	1.338
F(000)	560.0	560.0
2θ range for data collection (°)	7.276 to 155.66	7.27 to 156.196
Index ranges	$-3 \le h \le 6$, $-13 \le k \le 13$, $-30 \le l \le 30$	$-6 \le h \le 6$, $-13 \le k \le 9$, $-30 \le l \le 30$
Reflections collected	5484	7298
Independent reflections	2528 [R_{int} = 0.0405, R_{sigma} = 0.0542]	2714 [R_{int} = 0.0520, R_{sigma} = 0.0615]
Data/restraints/ parameters	2528/0/170	2714/0/182
Goodness-of-fit on F^2	1.102	1.064
Completeness (%)	99.4	99.8
[a]Final R indexes [I>=2σ (I)]	R_1 = 0.0585	R_1 = 0.0486
[b]Final R indexes [all data]	wR_2 = 0.1668	wR_2 = 0.1410
Largest diff. peak/hole (e Å^{-3})	0.48/−0.55	0.44/−0.33
Flack parameter	−0.2(2)	−0.2(2)

[a]$R_1 = \sum||F_o|-|F_c||/\sum|F_o|$. [b]$wR_2 = \{[\sum w(F_o^2-F_c^2)^2]/\sum[w(F_o^2)]\}^{1/2}$; $w = 1/[\sigma^2(F_o^2)+(aP)^2+bP]$, where $P = [\max(F_o^2, 0) + 2F_c^2]/3$ for all data.

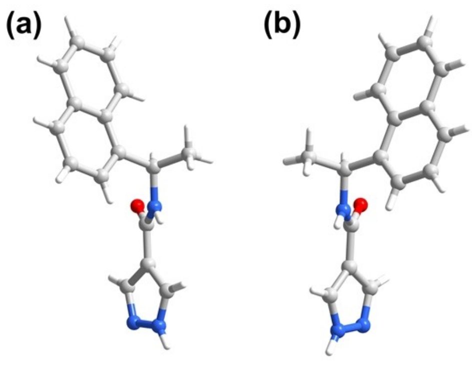

Fig. S25 Molecular structures of (a) R-HL_2 and (b) S-HL_2. Colour codes: grey, C; blue, N; red, O; white, H.

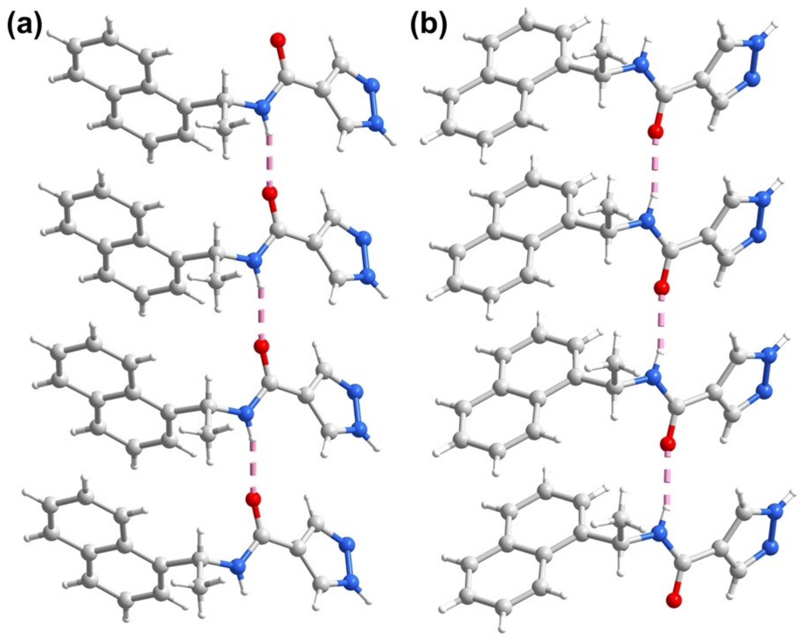

Fig. S26 Packing modes of (a) R-HL_2 and (b) S-HL_2. Dashed lines showing hydrogen-bonding interactions the between adjacent molecules. Colour codes: grey, C; blue, N; red, O; white, H.

6.3 Structural Determination of R-1/S-1

Table S3 Crystallographic data and refinement parameters of **R-1/S-1**.

Item	**R-1**	**S-1**
CCDC number	2378501	2378502
Temperature (K)	100.0(2)	100.1(4)
Empirical formula	$C_{112}H_{119}Cu_9N_{27}O_{11}$	$C_{112}H_{120}Cu_9N_{27}O_{11}$
Formula weight	2591.19	2592.20
Crystal system	monoclinic	monoclinic
Space group	$P2_1$	$P2_1$
a (Å)	9.09800(10)	9.0903(2)
b (Å)	19.5551(3)	19.5430(4)
c (Å)	33.1683(5)	33.1835(9)
β (°)	97.6660(10)	97.787(2)
Volume (Å^3)	5848.31(14)	5840.7(2)
Z	2	2
ρ_{calc} (g/cm^3)	1.471	1.474
F(000)	2658.0	2660.0
2θ range for data collection (°)	7.026 to 157.844	7.026 to 156.262
Index ranges	$-10 \leq h \leq 11$, $-24 \leq k \leq 24$, $-42 \leq l \leq 30$	$-11 \leq h \leq 11$, $-22 \leq k \leq 24$, $-38 \leq l \leq 42$
Reflections collected	60856	50804
Independent reflections	22685 [R_{int} = 0.1268, R_{sigma} = 0.0755]	20099 [R_{int} = 0.0970, R_{sigma} = 0.0754]
Data/restraints/ parameters	22685/1/1257	20099/2794/1558
Goodness-of-fit on F^2	1.039	1.041
Completeness (%)	99.6	99.3
^{a}Final R indexes [I>=2σ (I)]	R_1 = 0.0845	R_1 = 0.1093
^{b}Final R indexes [all data]	wR_2 = 0.2024	wR_2 = 0.2828
Largest diff. peak/hole (e Å^{-3})	1.06/−0.98	1.52/−1.02
Flack parameter	0.05(4)	0.10(7)

$^aR_1 = \sum||F_o|-|F_c||/\sum|F_o|$. $^bwR_2 = \{[\sum w(F_o^2-F_c^2)^2]/\sum[w(F_o^2)]\}^{1/2}$; $w = 1/[\sigma^2(F_o^2)+(aP)^2+bP]$, where $P = [\max(F_o^2, 0) + 2F_c^2]/3$ for all data.

Table S4 Bond lengths (Å) and angles (°) in the single crystals of **R-1**/**S-1**.

	**R-1**		
Cu(1)–N(2)	1.821(4)	Cu(1)–N(15)	1.822(4)
Cu(2)–N(3)	1.821(4)	Cu(2)–N(27)	1.829(4)
Cu(3)–N(9)	1.801(5)	Cu(3)–N(19)	1.808(4)
Cu(4)–N(17)	1.843(4)	Cu(4)–N(23)	1.832(4)
Cu(5)–N(7)	1.827(4)	Cu(5)–N(25)	1.821(4)
Cu(6)–N(13)	1.833(4)	Cu(6)–N(20)	1.833(4)
Cu(7)–N(18)	1.817(5)	Cu(7)–N(22)	1.817(4)
Cu(8)–N(11)	1.815(4)	Cu(8)–N(24)	1.821(4)
Cu(9)–N(5)	1.833(4)	Cu(9)–N(21)	1.827(5)
N(2)–Cu(1)–N(15)	176.2(3)	N(3)–Cu(2)–N(27)	175.1(3)
N(9)–Cu(3)–N(19)	174.1(3)	N(17)–Cu(4)–N(23)	175.3(3)
N(7)–Cu(5)–N(25)	176.5(3)	N(13)–Cu(6)–N(20)	176.8(3)
N(18)–Cu(7)–N(22)	178.2(3)	N(11)–Cu(8)–N(24)	175.1(3)
N(5)–Cu(9)–N(21)	177.1(3)		
	**S-1**		
Cu(1)–N(17)	1.826(4)	Cu(1)–N(23)	1.822(4)
Cu(2)–N(12)	1.820(4)	Cu(2)–N(26)	1.817(4)
Cu(3)–N(3)	1.821(4)	Cu(3)–N(22)	1.819(4)
Cu(4)–N(5)	1.813(4)	Cu(4)–N(24)	1.818(4)
Cu(5)–N(2)	1.820(4)	Cu(5)–N(11)	1.816(4)
Cu(6)–N(16)	1.818(4)	Cu(6)–N(21)	1.822(4)
Cu(7)–N(8)	1.815(4)	Cu(7)–N(25)	1.814(4)
Cu(8)–N(18)	1.818(4)	Cu(8)–N(20)	1.820(4)
Cu(9)–N(10)	1.820(4)	Cu(9)–N(27)	1.820(4)
N(17)–Cu(1)–N(23)	175.5(5)	N(12)–Cu(2)–N(26)	178.9(5)
N(3)–Cu(3)–N(22)	177.0(5)	N(5)–Cu(4)–N(24)	174.5(5)
N(2)–Cu(5)–N(11)	175.9(5)	N(16)–Cu(6)–N(21)	176.4(5)
N(8)–Cu(7)–N(25)	174.4(5)	N(18)–Cu(8)–N(20)	176.3(5)
N(10)–Cu(9)–N(27)	174.4(5)		

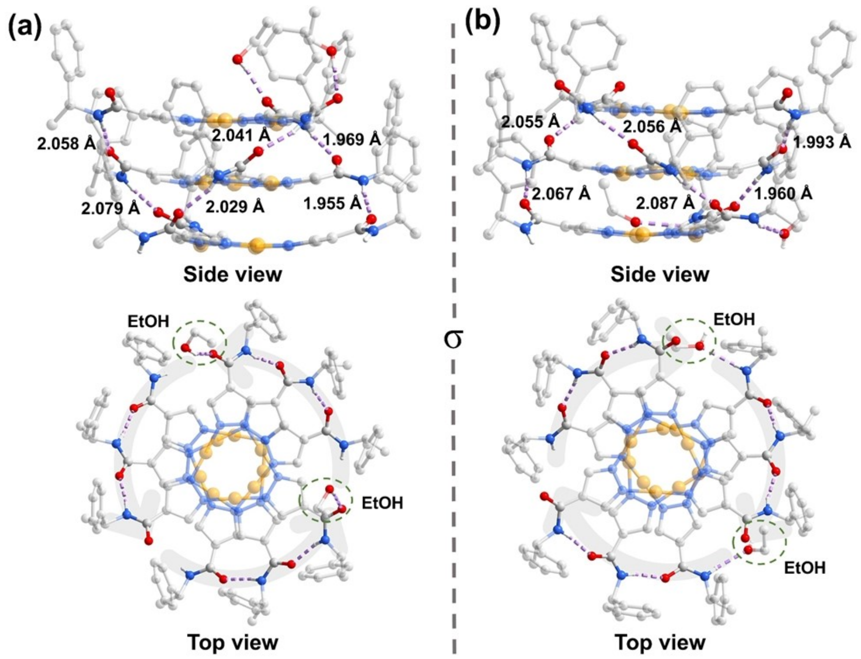

Fig. S27 Asymmetric units of (a) **R-1** and (b) **S-1**, which are both built up of three Cu(I) CTC molecules and two EtOH molecules. The pink dashed lines highlight hydrogen bonding interactions. Colour codes: orange, Cu; grey, C; blue, N; red, O; white, H. For clarity, some H atoms have been omitted. Ethanol molecules also participate in the formation of hydrogen-bonding networks between the adjacent asymmetric units, see Figure S31.

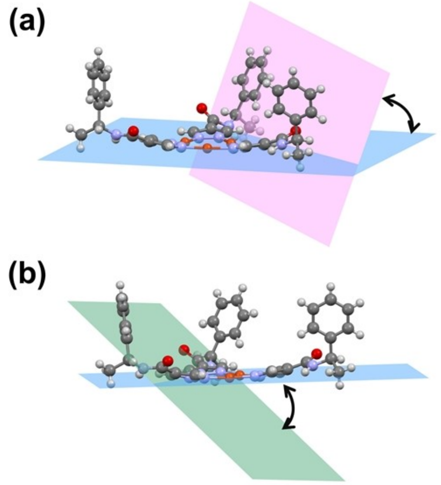

Fig. S28 Schematic diagram of dihedral angles between (a) the pyrazolate planes and phenyl rings, (b) the pyrazolate planes and amide groups in **R-1**.

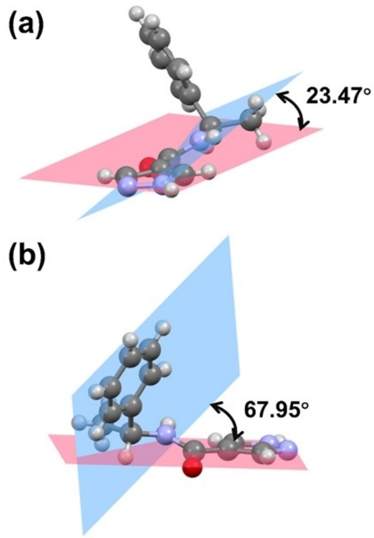

Fig. S29 Dihedral angles between the (a) amide group and pyrazole planes, (b) phenyl ring and pyrazole planes in R-HL_1.

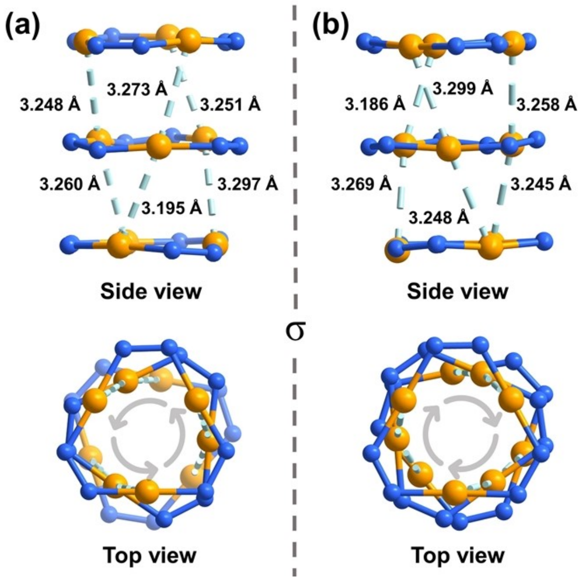

Fig. S30 Stacking modes of Cu_3N_6 units in the asymmetric units of (a) **R-1** and (b) **S-1**. The blue dashed lines highlight intermolecular Cu···Cu interactions. Colour codes: orange, Cu; blue, N.

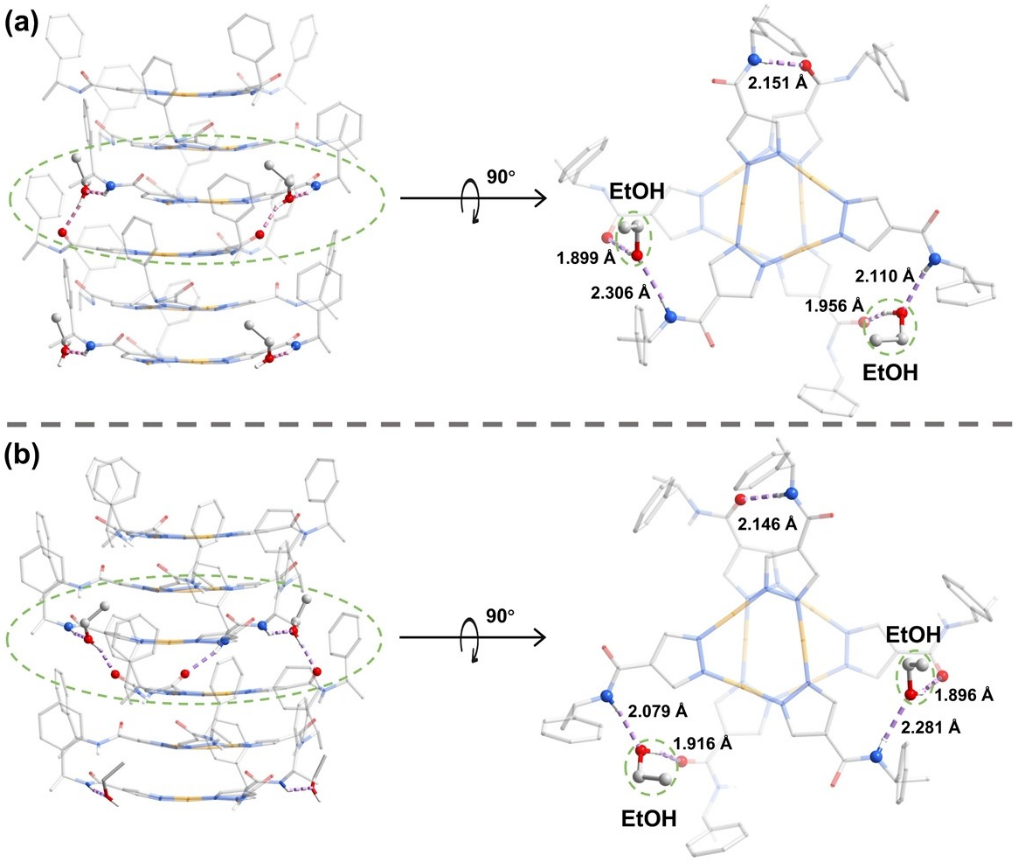

Fig. S31 The hydrogen-bonding network between the adjacent $[Cu_3(R\text{-}L_1)_3]_3\cdot(EtOH)_2$ units in (a) **R-1** and (b) **S-1**. In addition to hydrogen bonds formed by adjacent amide groups, the hydroxyl groups from ethanol molecules also participate in the hydrogen-bonding network. These hydroxyl groups act as both hydrogen bond donors and acceptors, forming hydrogen bonds through the N–H and C=O groups of adjacent amide groups. The pink dashed lines highlight hydrogen bonding interactions. Colour codes: orange, Cu; grey, C; blue, N; red, O; white, H. For clarity, some H atoms have been omitted.

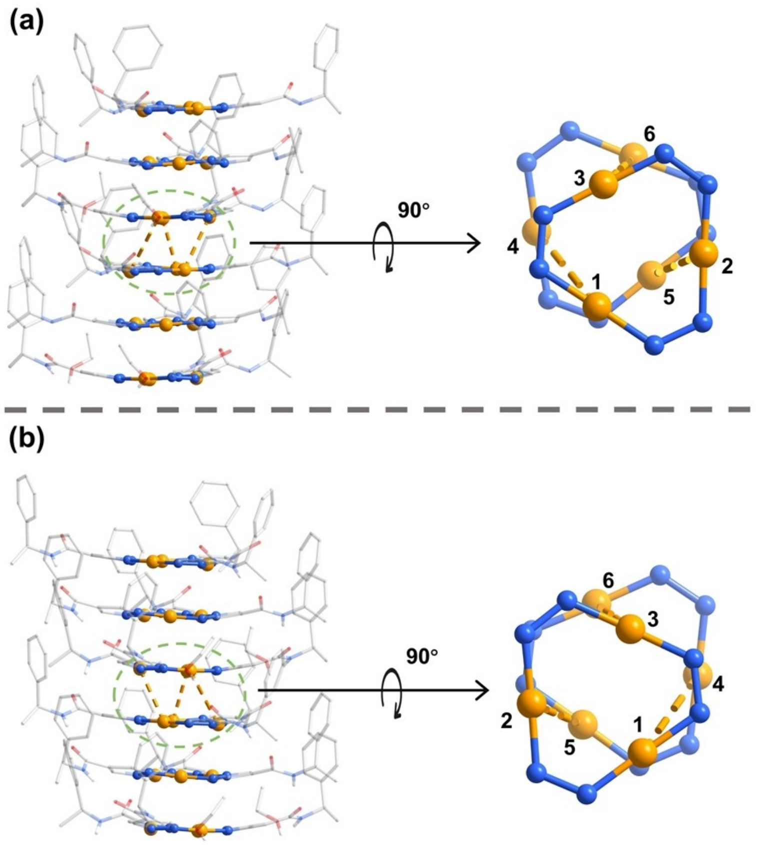

Fig. S32 The metallophilic network between the adjacent $[Cu_3(R\text{-}L_1)_3]_3\cdot(EtOH)_2$ units in (a) **R-1** and (b) **S-1**. The orange dashed lines highlight intermolecular Cu···Cu interactions. Intermolecular Cu···Cu distances (Å), **R-1**: 1–4 = 4.056, 2–5 = 3.297, 3–6 = 3.079; **S-1**: 1–4 = 4.053, 2–5 = 3.301, 3–6 = 3.074. Colour codes: orange, Cu; grey, C; blue, N; red, O; white, H. For clarity, some H atoms have been omitted.

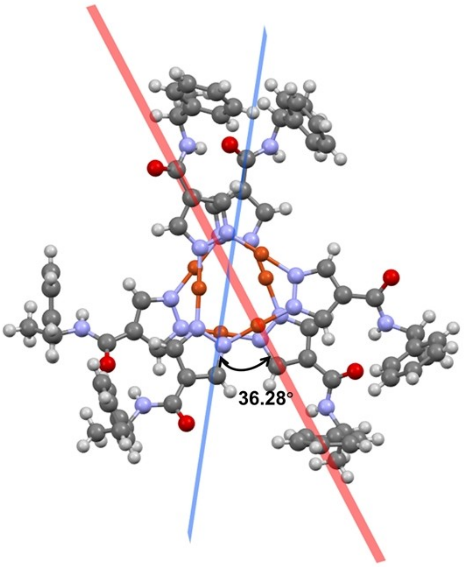

Fig. S33 The twist angle between adjacent Cu(I) CTCs in **R-1**. Colour codes: orange, Cu; dark grey, C; purple, N; red, O; light grey, H.

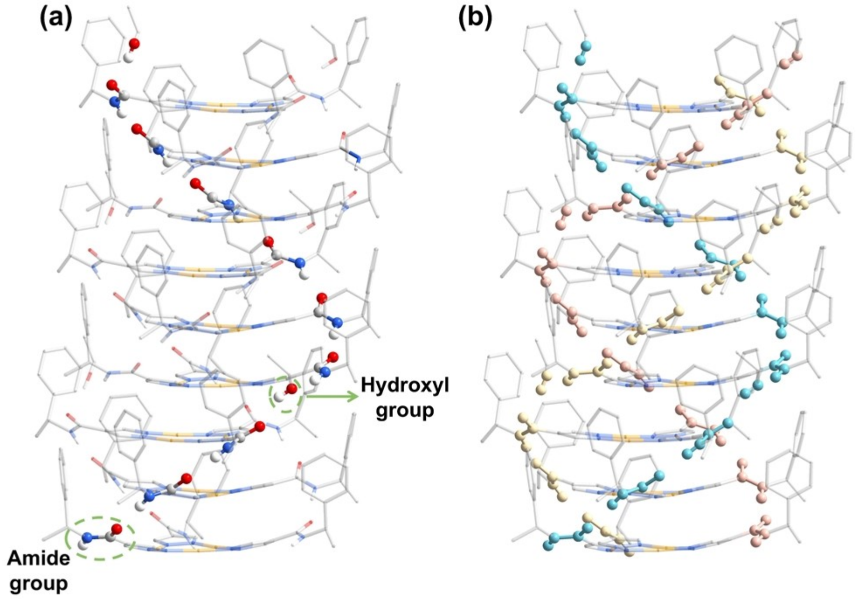

Fig. S34 (a) A full turn of a hydrogen-bonded helical chain in **R-1** formed by nine amide groups and two hydroxyl groups. (b) Full turns of triple-helical hydrogen-bonding networks of the peripheral ligands in **R-1**. The entire triple-helical network is formed by the intertwining of three hydrogen-bonded helical chains. Colour codes for Figure S34a: orange, Cu; grey, C; blue, N; red, O; white, H. In Figure S34b, each helical chain is depicted in the same colour to emphasize the triple-helical assembly. For clarity, some hydrogen atoms have been omitted.

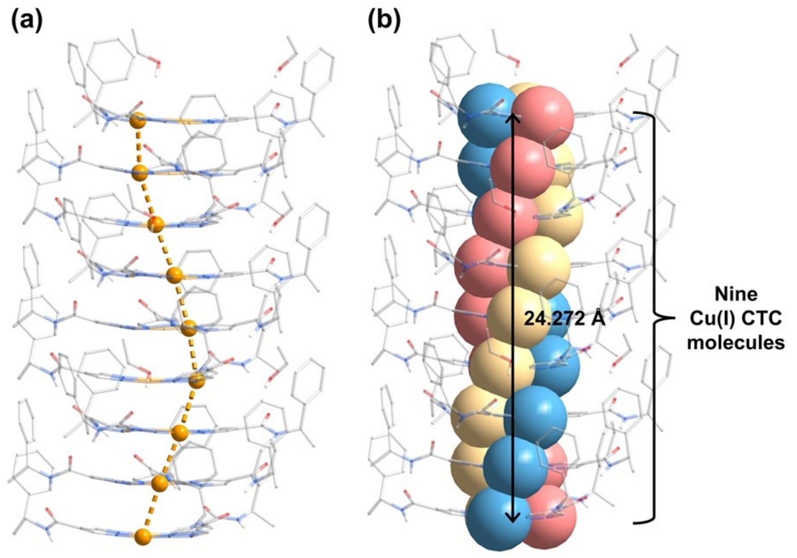

Fig. S35 (a) A full turn of a metallophilically bonded helical chain in **R-1** formed by nine Cu^{+} ions. (b) Full turns of triple-helical metallophilic networks of Cu_3N_6 units in **R-1**. The complete triple-helical network is formed by the intertwining of three metallophilically bonded helical chains. Colour codes for Figure S35a: orange, Cu; grey, C; blue, N; red, O; white, H. In Figure S35b, to emphasize the triple-helical assembly, each helical chain is depicted in the same colour. For clarity, some H atoms have been omitted.

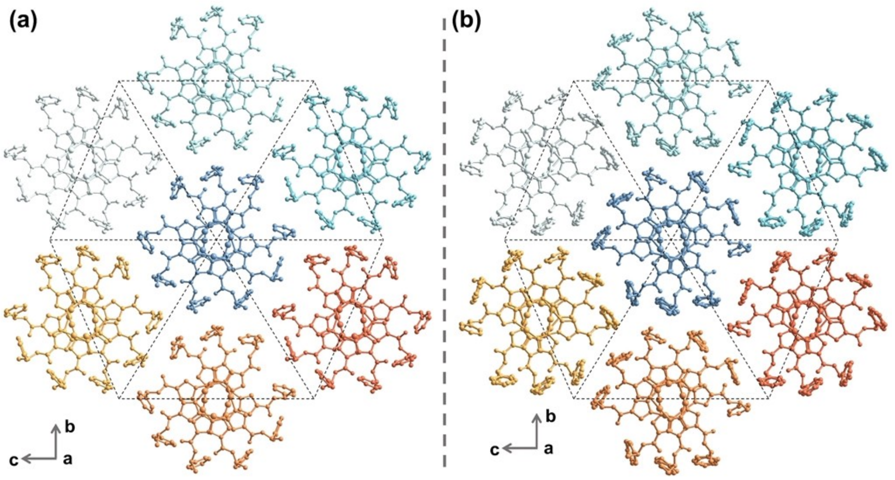

Fig. S36 Pseudo-hexagonal packing of the triple helices along the *a*-axis in (a) **R-1** and (b) **S-1**.

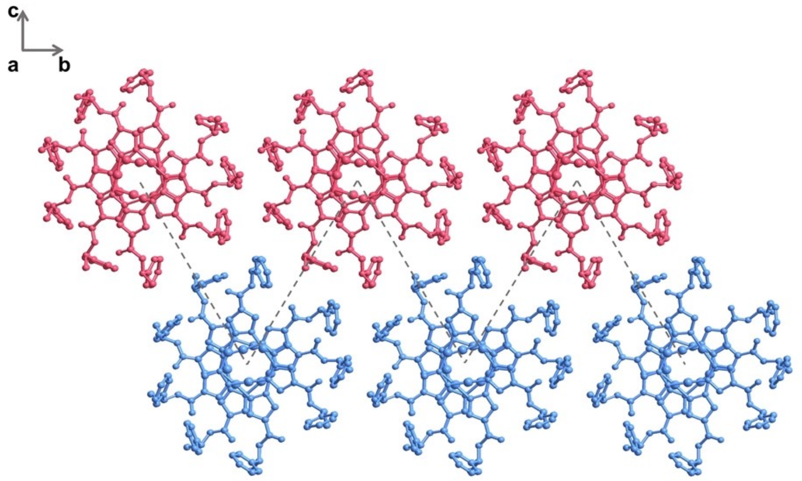

Fig. S37 Crystal packing of **R-1** in the $P2_1$ space group, showing the arrangement of molecules along the 2_1 axis parallel to the *b*-axis.

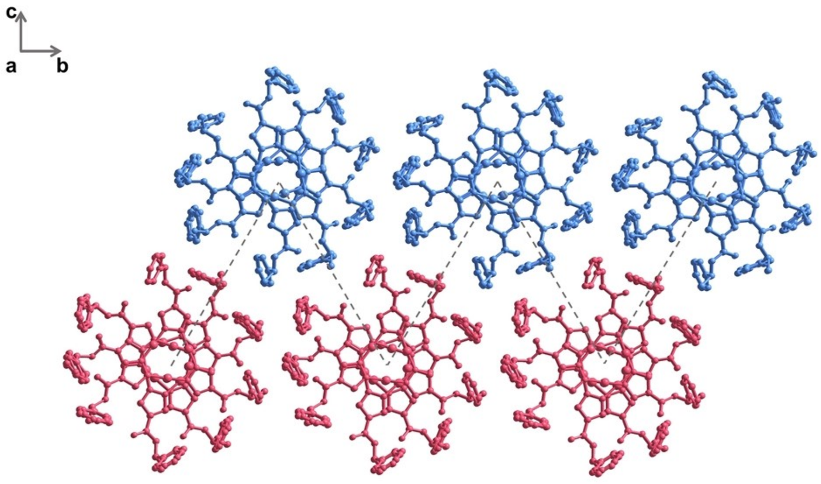

Fig. S38 Crystal packing of **S-1** in the $P2_1$ space group, showing the arrangement of molecules along the 2_1 axis parallel to the *b*-axis.

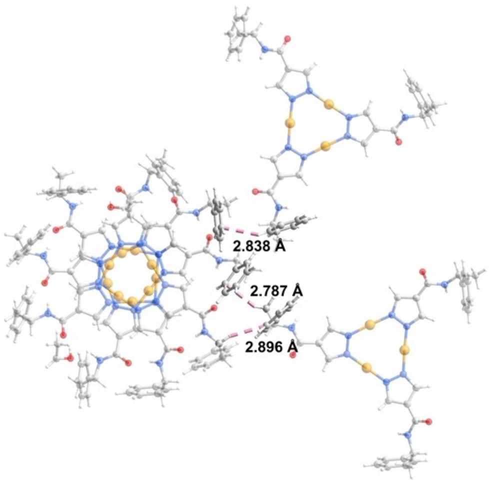

Fig. S39 The pink dashed lines highlight C–H···π interactions in **R-1**. Colour codes: orange, Cu; grey, C; blue, N; red, O; white, H.

7. SEM images

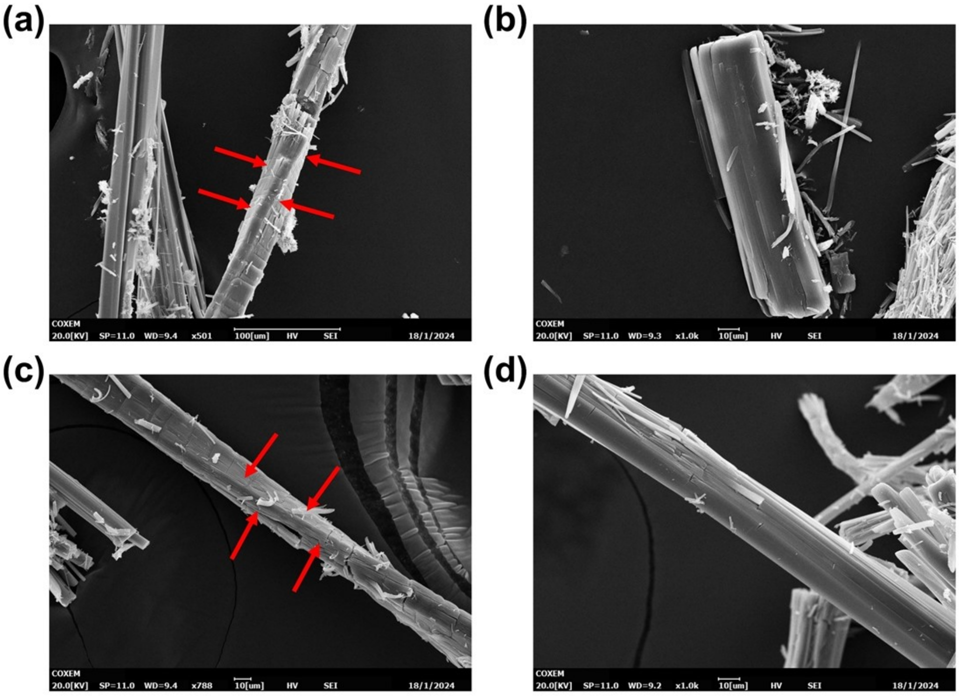

Fig. S40 SEM images of (a, b) **R-1** and (c, d) **S-1.** In Figures a, c, the crystal of **R/S-1** show hierarchical chirality on the macroscopic scale. The chiral geometry may arise from the intrinsic crystal face position and their extrinsic differential growth, while uniform growth results in 'recessive chirality'.[3]

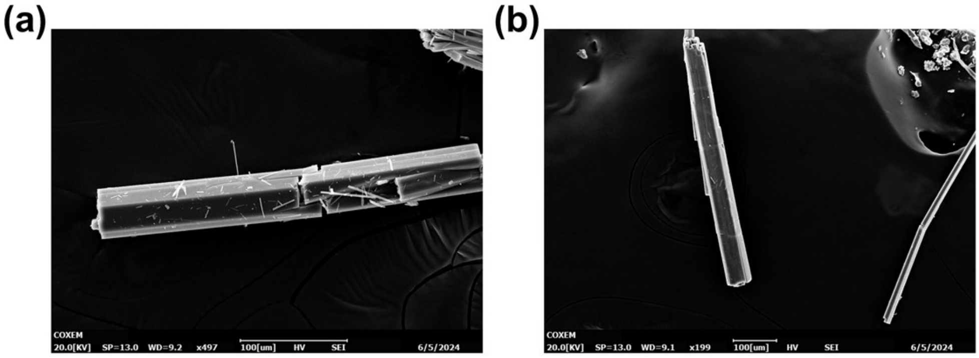

Fig. S41 SEM images of (a) **R-2** and (b) **S-2**.

8. Photophysical Investigation

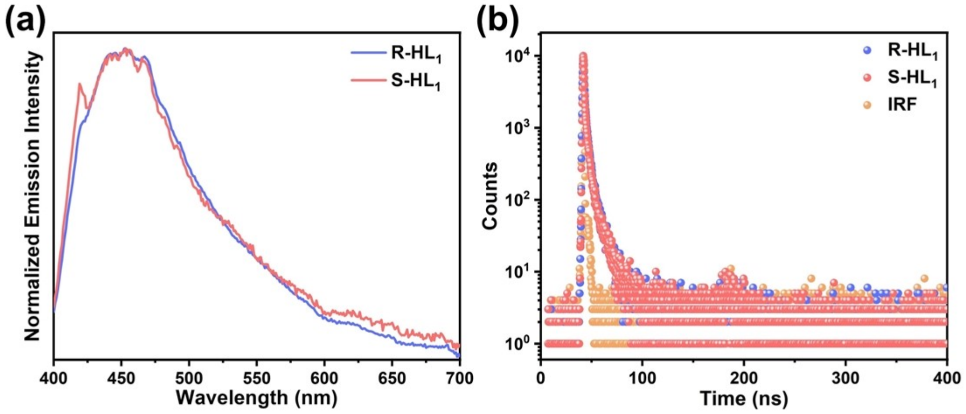

Fig. S42 (a) Normalized emission spectra of R-HL_1/S-HL_1 when excited at 370 nm. (b) Emission decay profiles of R-HL_1/S-HL_1 (τ_{ave} = 0.27 ns for R-HL_1, τ_{ave} = 0.28 ns for S-HL_1). IRF: instrument response function.

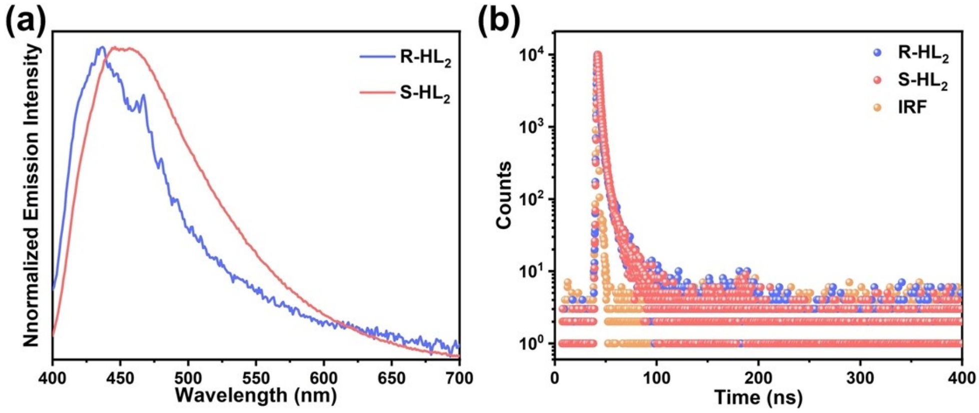

Fig. S43 (a) Normalized emission spectra of R-HL_2/S-HL_2 when excited at 370 nm. (b) Emission decay profiles of R-HL_2/S-HL_2 (τ_{ave} = 0.53 ns for R-HL_2, τ_{ave} = 1.41 ns for S-HL_2). IRF: instrument response function.

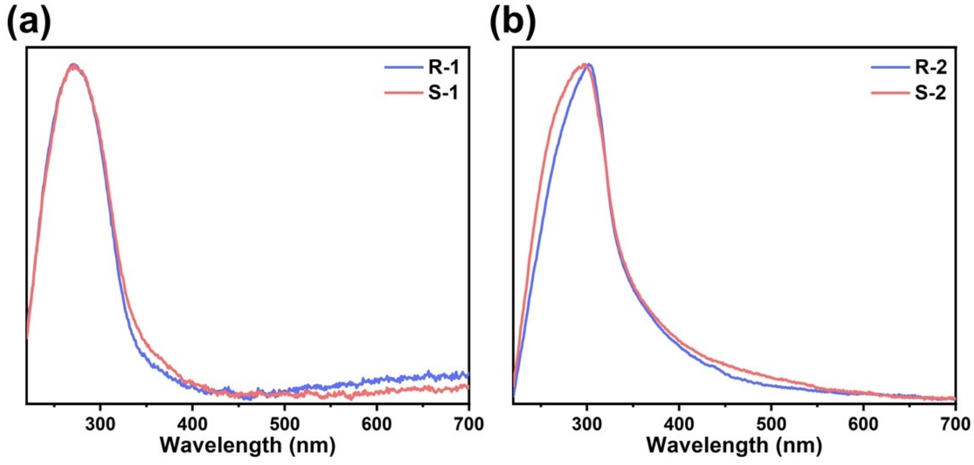

Fig. S44 The normalized steady-state UV-vis absorption spectra of (a) **R-1/S-1** and (b) **R-2/S-2** in air at room temperature.

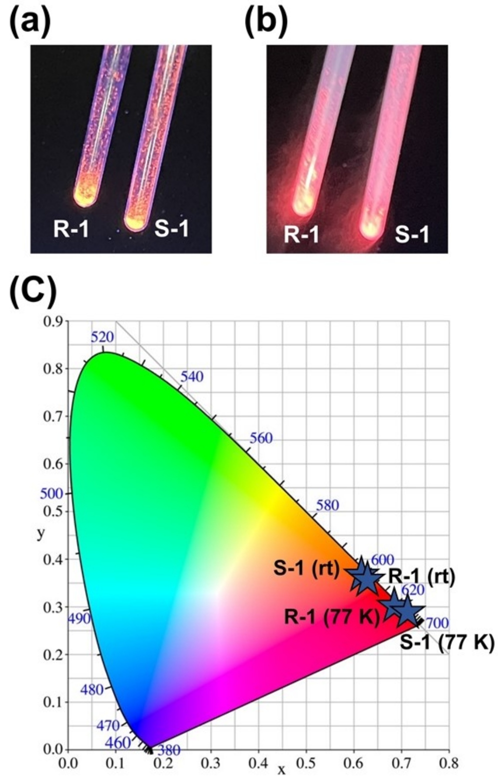

Fig. S45 The photographs of **R-1** and **S-1** in NMR tubes under the UV-light excitation of 310 nm at (a) room temperature (rt) and (b) 77 K. (c) CIE chromaticity diagram of **R-1** and **S-1** under λ = 310 nm excitation wavelength at 77 K and rt.

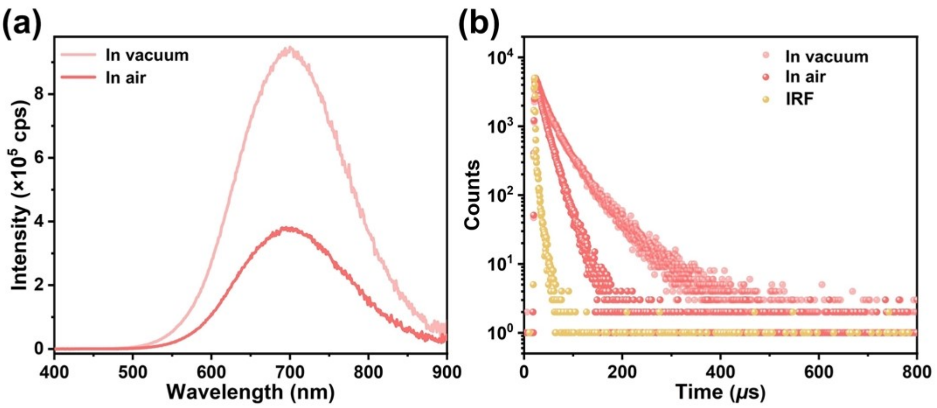

Fig. S46 Comparison of (a) emission spectra and (b) lifetime decay curves of **S-1** in vacuum and air at room temperature. IRF: instrument response function.

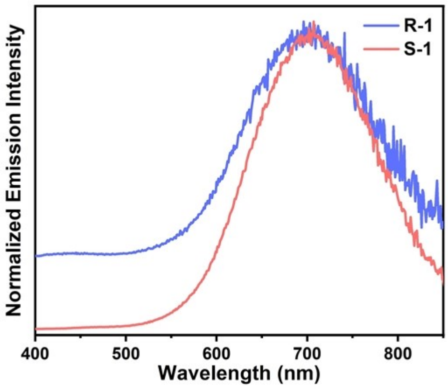

Fig. S47 The normalized emission spectra of **R-1** and **S-1** when excited at 370 nm in air at room temperature.

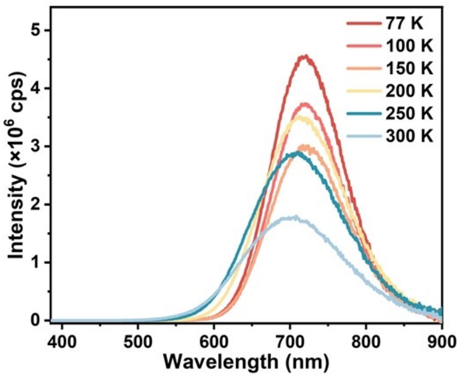

Fig. S48 Temperature-dependent emission spectra (λ_{ex} = 310 nm) of **R-1** in vacuum.

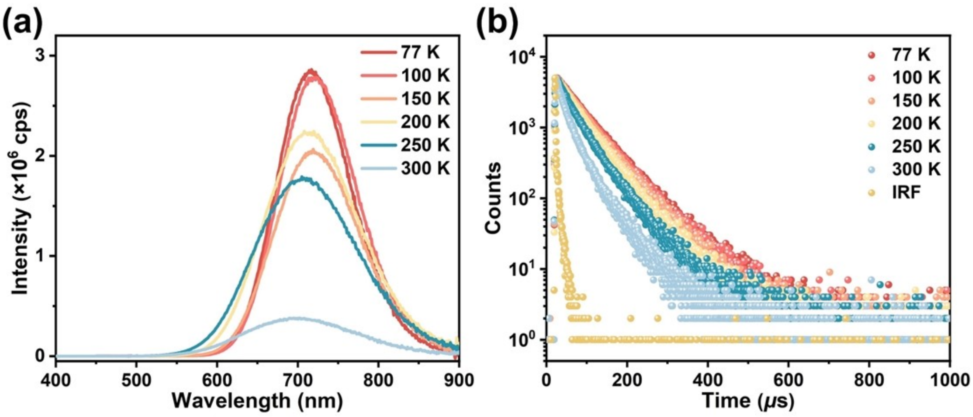

Fig. S49 Temperature-dependent (a) emission spectra (λ_{ex} = 310 nm) and (b) lifetime decay curves of **S-1** in vacuum. IRF: instrument response function.

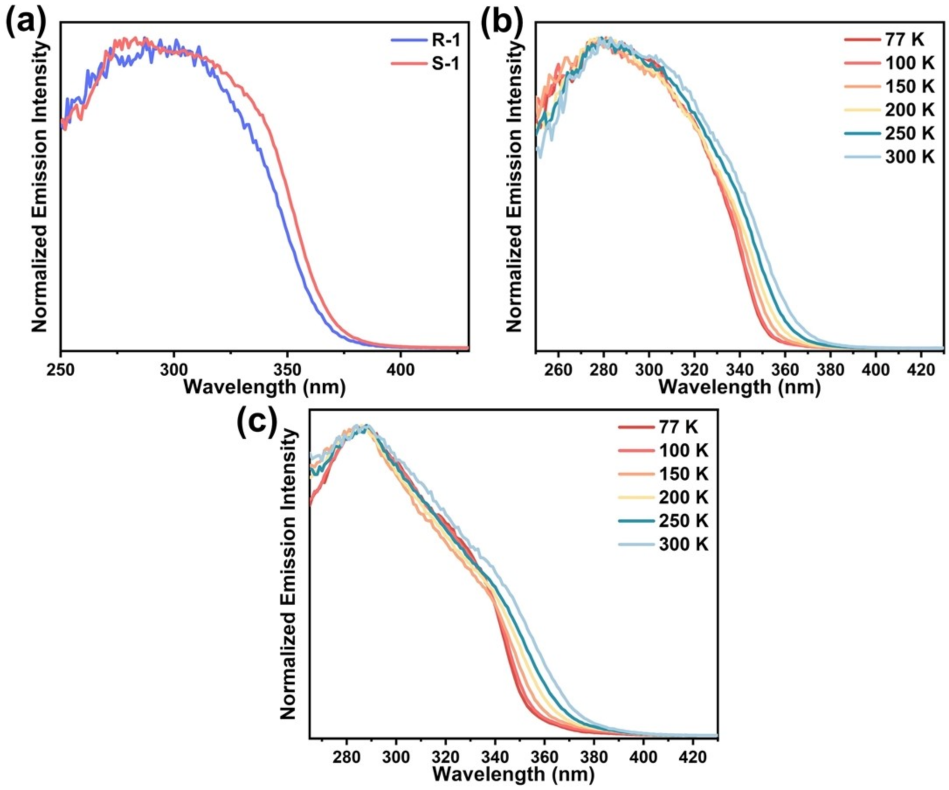

Fig. S50 (a) The normalized excitation spectra of **R-1** and **S-1** in air at room temperature. Temperature-dependent normalized excitation spectra of (b) **R-1** and (c) **S-1** in vacuum.

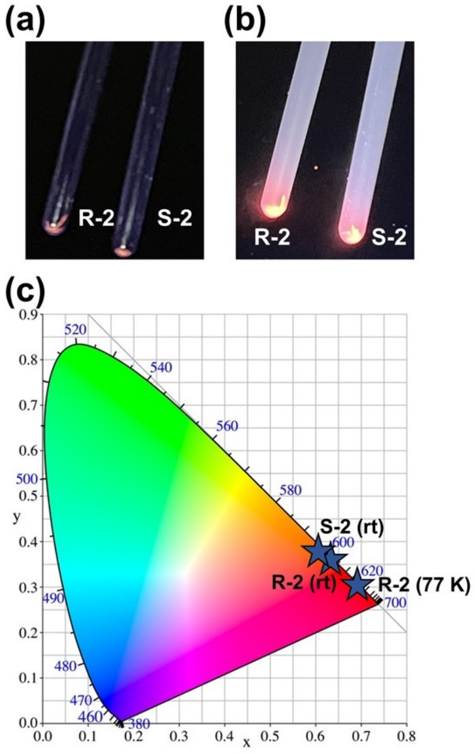

Fig. S51 The photographs of **R-2** and **S-2** in NMR tubes under the UV-light excitation of 310 nm at (a) room temperature (rt) and (b) 77 K. (c) CIE chromaticity diagram of **R-2** and **S-2** under λ = 310 nm excitation wavelength at 77 K and rt.

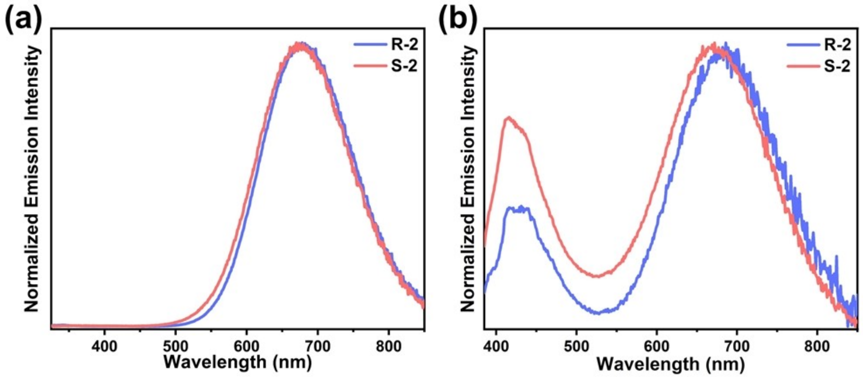

Fig. S52 The normalized emission spectra of **R-2** and **S-2** when excited at (a) 310 nm and (b) 370 nm in air at room temperature.

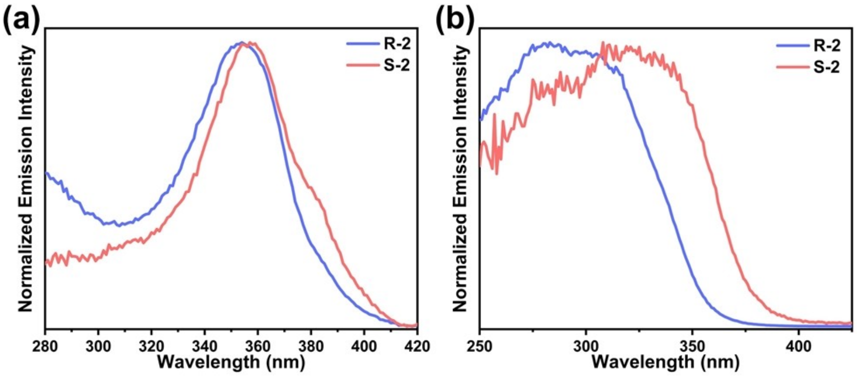

Fig. S53 The excitation spectra of (a) high-energy emission and (b) low-energy emission of **R-2** and **S-2** in air at room temperature.

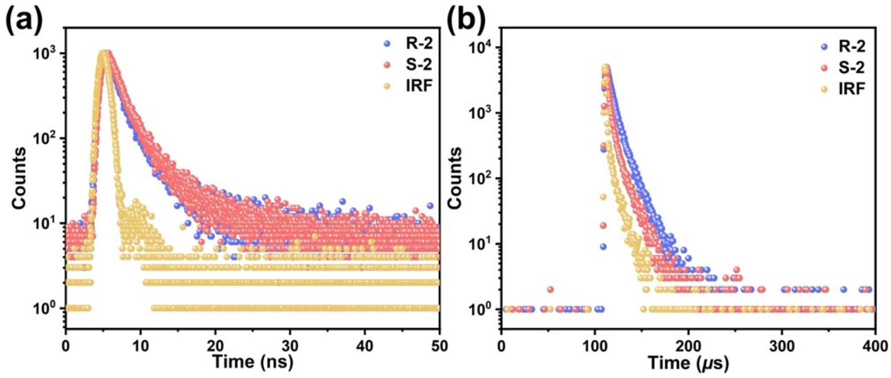

Fig. S54 Emission decay profiles of (a) high-energy emission and (b) low-energy emission of **R-2** and **S-2** in air at room temperature. IRF: instrument response function.

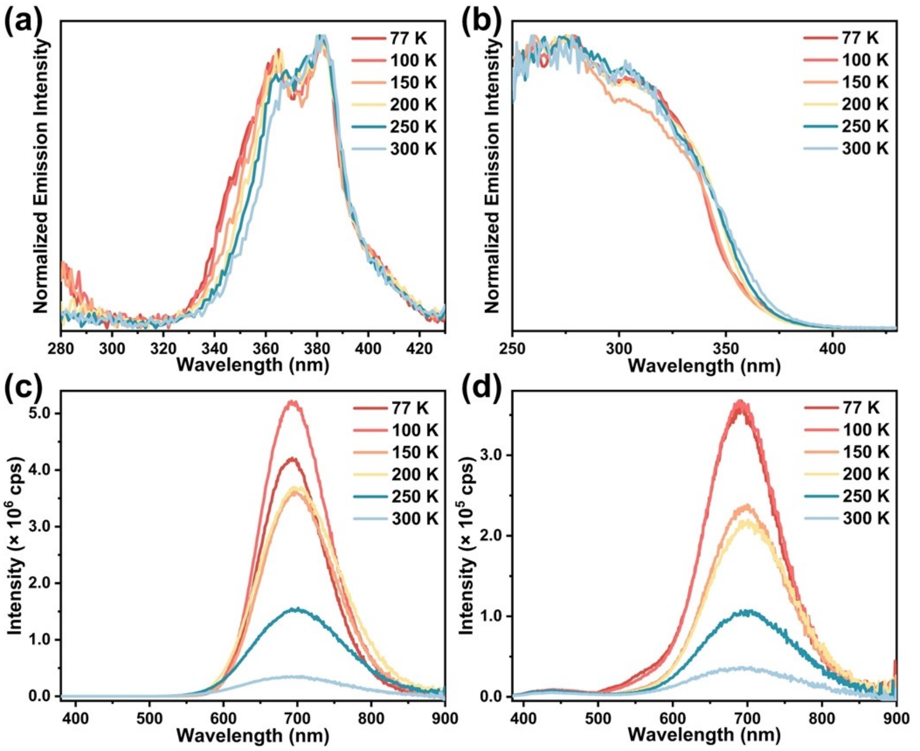

Fig. S55 Temperature-dependent normalized excitation spectra of (a) high-energy emission and (b) low-energy emission of **R-2** in vacuum. Temperature-dependent emission spectra of **R-2** when excited at (c) 310 nm and (d) 370 nm in vacuum.

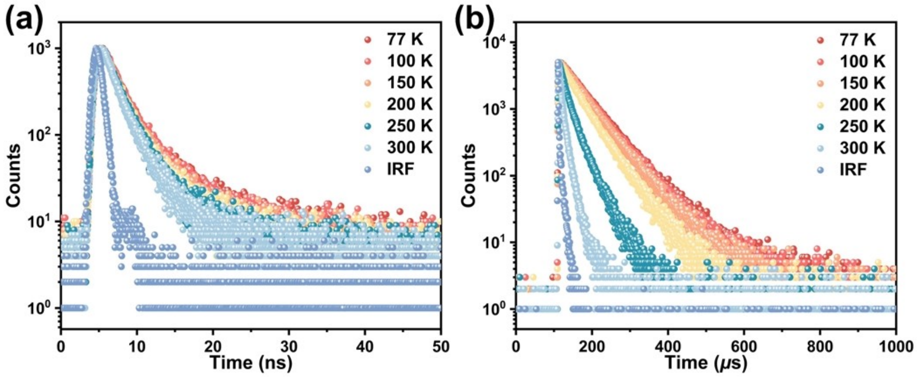

Fig. S56 Temperature-dependent emission decay profiles of (a) high-energy emission and (b) low-energy emission of **R-2** in vacuum. IRF: instrument response function.

Table S5 The summary of photophysical data of **R-1**, **S-1**, **R-2** and **S-2** in air at room temperature.

	λ_{Abs} (nm)	λ_{Ex} (nm)	λ_{Em} (nm)	τ_{ave}	Φ (%)
**R-1**	272	294	699	12.27 μs	21.7
**S-1**	271	290	700	14.23 μs	26.6
**R-2**	302	354	426	2.27 ns	13.2
		300	680	6.24 μs	
**S-2**	298	358	424	2.36 ns	12.9
		314	677	3.67 μs	

Table S6 The summary of photophysical data of **R-1** at different temperatures between 77 K and 300 K in vacuum.

T (K)	λ_{Ex} (nm)	λ_{Em} (nm)	τ_{ave} (μs)
77	287	719	63.82
100	290	718	64.25
150	287	722	59.46
200	288	716	55.21
250	288	708	48.30
300	291	702	41.04

Table S7 The summary of photophysical data of **S-1** at different temperatures between 77 K and 300 K in vacuum.

T (K)	λ_{Ex} (nm)	λ_{Em} (nm)	τ_{ave} (μs)
77	288	717	62.32
100	288	718	60.48
150	288	717	57.42
200	289	714	56.03
250	289	706	45.42
300	290	700	37.12

Table S8 The summary of photophysical data of **R-2** at different temperatures between 77 K and 300 K in vacuum.

T (K)	λ_{Ex} (nm)	λ_{Em} (nm)	τ_{ave}
77	381	440	3.07 ns
	281	694	61.63 μs
100	382	439	2.90 ns
	280	694	59.65 μs
150	382	440	2.70 ns
	276	696	56.18 μs
200	381	441	2.46 ns
	278	700	46.29 μs
250	381	440	2.25 ns
	281	697	25.80 μs
300	382	440	2.00 ns
	280	696	7.92 μs

Table S9 The luminescence lifetimes of **R-1**, **S-1**, **R-2** and **S-2** in air at room temperature.

	Lifetime 1 (τ_1)	Lifetime 2 (τ_2)	τ_1%	τ_2%
**R-1**	3.39 μs	15.16 μs	24.57	75.43
**S-1**	4.73 μs	17.74 μs	26.94	73.06
**R-2**	1.17 ns	5.24 ns	72.94	27.06
	3.70 μs	10.14 μs	60.51	39.49
**S-2**	1.27 ns	5.30 ns	72.82	27.18
	2.42 μs	10.67 μs	84.78	15.22

Table S10 The luminescence lifetimes of **R-1** at different temperatures between 77 K and 300 K in vacuum.

T (K)	Lifetime 1 (τ_1/μs)	Lifetime 2 (τ_2/μs)	τ_1%	τ_2%
77	17.62	69.12	10.30	89.70
100	23.94	69.29	11.11	88.89
150	18.44	63.97	9.89	90.11
200	17.80	60.25	11.86	88.14
250	15.15	55.45	17.74	82.26
300	10.88	51.26	25.30	74.70

Table S11 The luminescence lifetimes of **S-1** at different temperatures between 77 K and 300 K in vacuum.

T (K)	Lifetime 1 ($\tau_1/\mu s$)	Lifetime 2 ($\tau_2/\mu s$)	τ_1%	τ_2%
77	19.07	66.53	8.88	91.12
100	14.54	64.51	8.07	91.93
150	13.60	60.43	6.42	93.58
200	12.01	56.03	6.63	93.37
250	18.14	53.97	23.87	76.13
300	13.28	56.74	45.14	54.86

Table S12 The luminescence lifetimes of high-energy emission of **R-2** at different temperatures between 77 K and 300 K in vacuum.

T (K)	Lifetime 1 (τ_1/ns)	Lifetime 2 (τ_2/ns)	τ_1%	τ_2%
77	1.69	6.88	73.39	26.61
100	1.67	6.35	73.68	26.32
150	1.59	5.88	74.24	25.76
200	1.54	5.35	75.78	24.22
250	1.48	5.07	78.87	21.13
300	1.36	4.33	78.14	21.86

Table S13 The luminescence lifetimes of low-energy emission of **R-2** at different temperatures between 77 K and 300 K in vacuum.

T (K)	Lifetime 1 ($\tau_1/\mu s$)	Lifetime 2 ($\tau_2/\mu s$)	τ_1%	τ_2%
77	47.04	75.83	49.31	50.69
100	45.39	71.62	45.64	54.36
150	32.42	61.79	19.09	80.91
200	17.81	50.78	13.63	86.37
250	15.17	35.46	47.59	52.41
300	3.42	13.41	54.92	45.08

9. Computational Details and Results

To give insight into the phtotophyscial process of **R-1**, density functional theory (DFT) and time-dependent DFT (TDDFT) calculations were performed using the Gaussian 09 software package.[4] At the same time, the Multiwfn 3.8 software packages[5] were further used for wave function analysis. For simulating the UV-vis absorption spectrum and exploring the insight of the emissive state, the dimer models of **R-1** were adopted. The geometry optimizations of ground and the first triplet excited states (S_0 and T_1 states) were carried out using the PBE0[6,7]-D3[8](BJ)[9] functional together with effective core potential (ECP) of LanL2dz basis set[10,11] was used for Cu atoms, while the 6-31G** basis set[12,13] was used for other atoms. Furthermore, the optimized S_0 dimer were also used for the geometry optimizations of excited state (T_1), with all atoms except two nine-membered rings were fixed. After geometrical optimization, frequency calculations were conducted to confirm that the optimized geometries are stable structures, as indicated by the absence of imaginary frequency. To obtain the simulated UV-vis spectrum, first 50 spin-allowed transitions were calculated based on optimized S_0 dimer. Meanwhile, the 10 spin-forbidden transitions were also obtained with optimized triplet state (T_1) structure. For accurately assigning excited state of **R-1**, electron density difference (EDD) maps (isovalue = 5.0×10^{-4} a.u.) were generated. The blue- and purple- regions represent electron transfer from former to latter when excitation.

The colour-filled surfaces of independent gradient model based on the Hirshfeld partition (IGMH) method[14] were drawn by VMD 1.93 program[15] for the selected dimer models based on the optimized dimer model generated by Multiwfn 3.8 software.

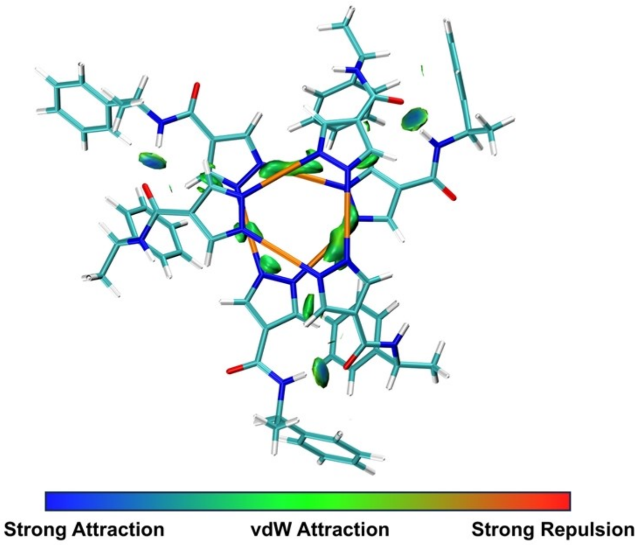

Fig. S57 IGMH analysis (isovalue = 0.01 a.u.) of two neighboring Cu(I) CTC molecules in **R-1**. The blue IGMH surfaces represents the prospective adaptive weak interaction, the green IGMH surfaces represents the van der Waals interaction. Colour codes: orange, Cu; cyan, C; blue, N; red, O; white, H.

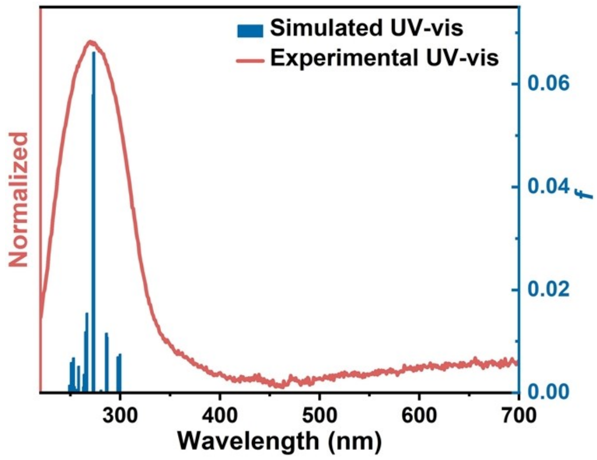

Fig. S58 The experiment solid-state UV-vis spectrum and the simulated absorption of the dimer of **R-1**. *f*: oscillator strength.

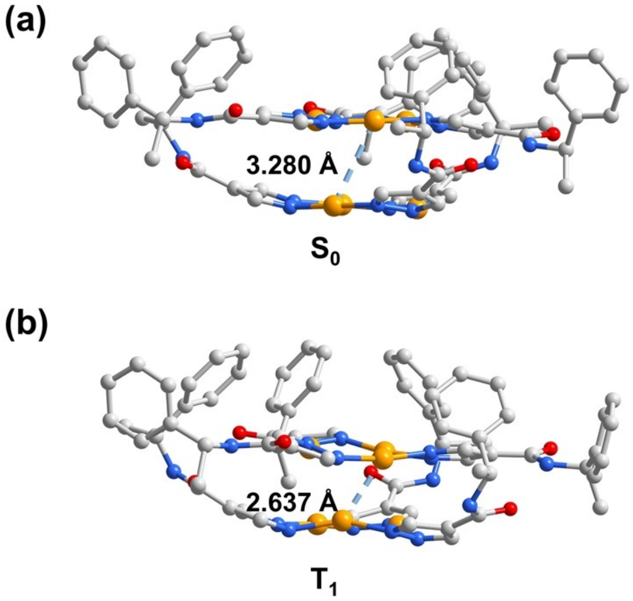

Fig. S59 Dimer of **R-1** in optimized (a) S_0 and (b) T_1 structure. Colour codes: orange, Cu; grey, C; blue, N; red, O.

Table S14 TDDFT result of selected $S_0 \rightarrow S_n$ transition for dimer of **R-1** in S_0 geometry

No.	λ (nm)	E (eV)	*f*	EDD		Assignment
				Top view	Side view	
1	299.8	4.14	0.01	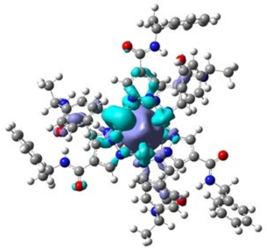	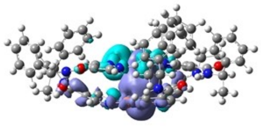	^{1}LMMCT
6	273.5	4.53	0.07	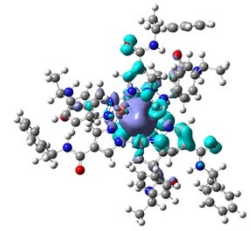	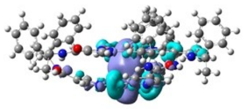	^{1}LMMCT
7	272.8	4.55	0.06	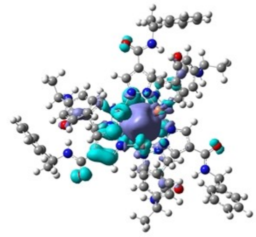	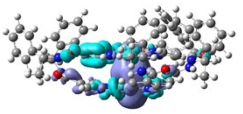	^{1}LMMCT

Table S15 TDDFT result of the $S_0 \rightarrow T_1$ transition for the dimer of **R-1** at the optimized T_1 geometry.

No.	λ (nm)	E (eV)	EDD		Assignment
			Top view	Side view	
1	524.1	2.37	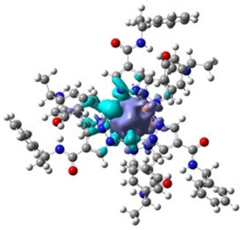	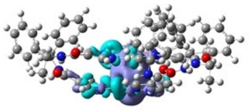	^{1}LMMCT

10. CD and CPL

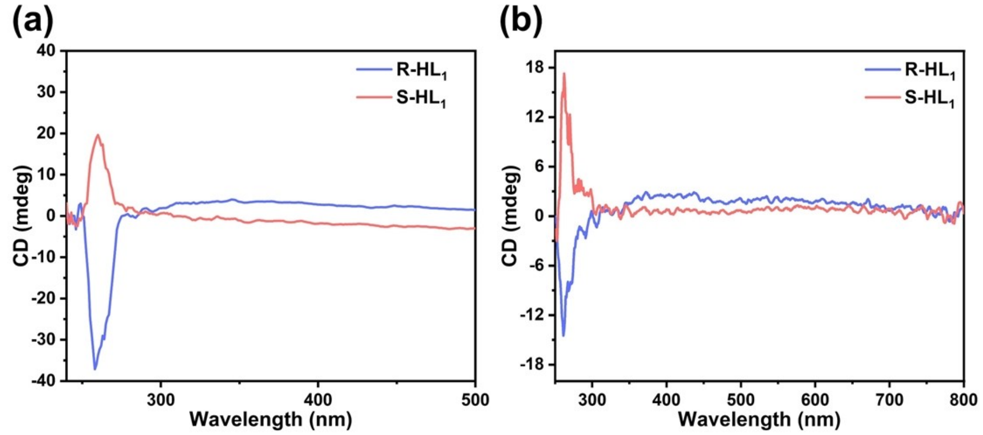

Fig. S60 CD spectra of R-HL_1/S-HL_1 in (a) MeOH solution and (b) KBr disk.

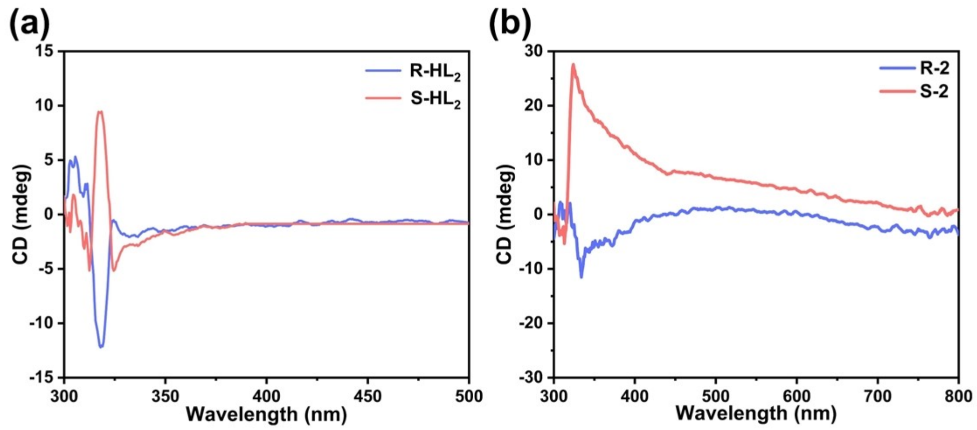

Fig. S61 (a) CD spectra of R-HL_2/S-HL_2 in MeOH. (b) CD spectra of **R-2** and **S-2** in solid.

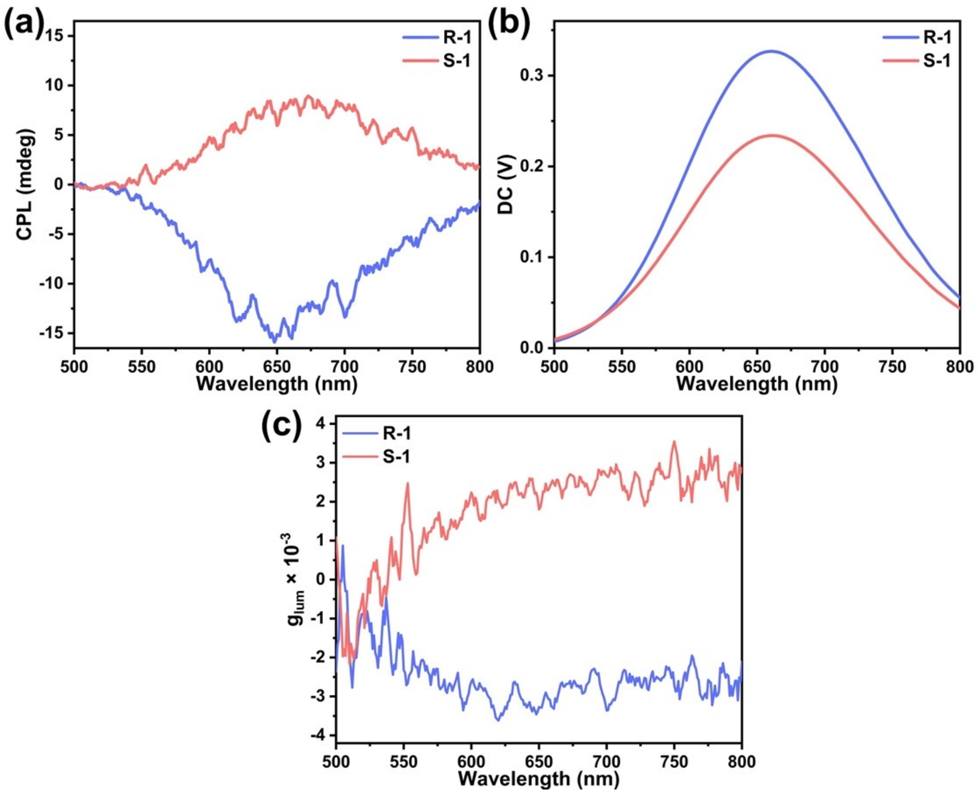

Fig. S62 The (a) CPL spectra, (b) DC spectra and (c) g_{lum} spectra of **R-1** and **S-1** in powder state with low-crystallinity upon excitation at 305 nm.

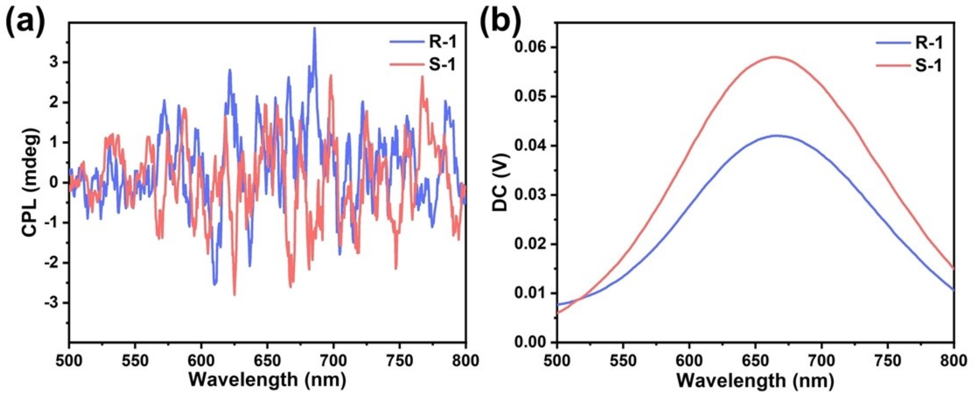

Fig. S63 The (a) CPL spectra and (b) DC spectra of **R-1** and **S-1** in powder state with non-crystallinity upon excitation at 305 nm.

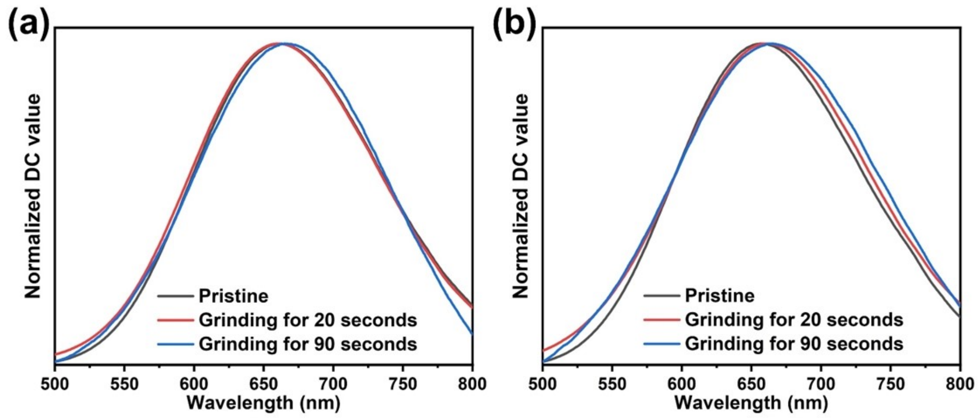

Fig. S64 The normalized DC spectra of (a) **R-1** and (b) **S-1** before grinding, after grinding for 20 seconds (red line) with low-crystallinity and further grinding for 70 seconds (bule line) with non-crystallinity. These indicate that grinding does not cause changes in the emission wavelength of **R-1**/**S-1**.

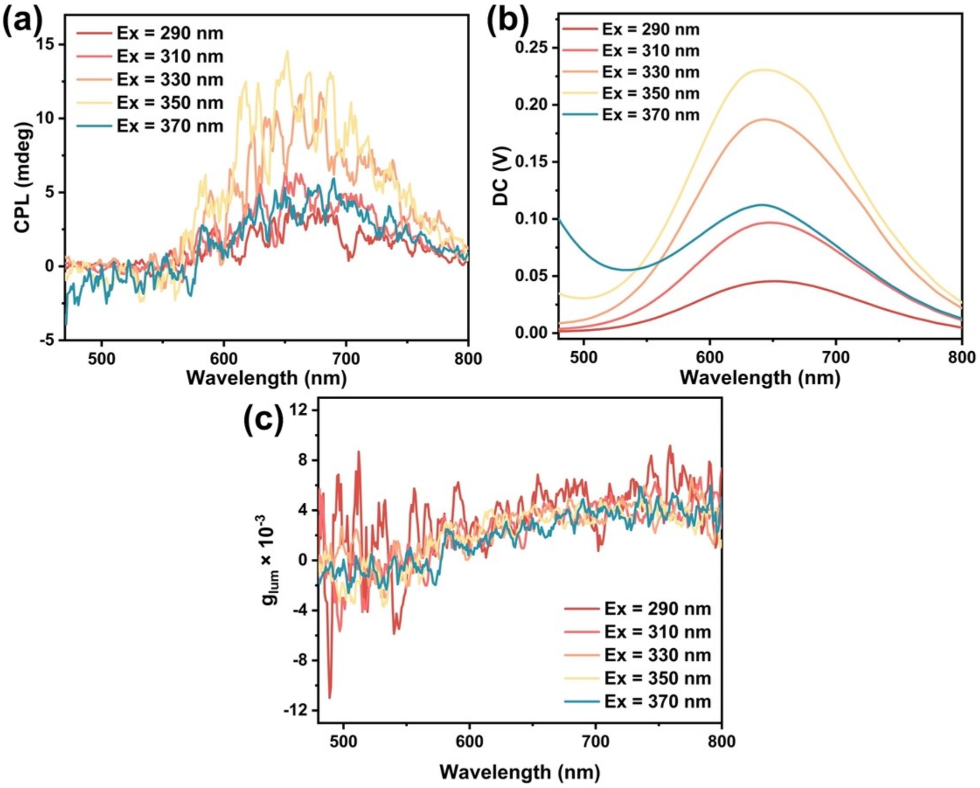

Fig. S65 (a) CPL emission spectra, (b) DC spectra and (c) g_{lum} spectra of **R-2** in crystal state upon different excitation wavelengths.

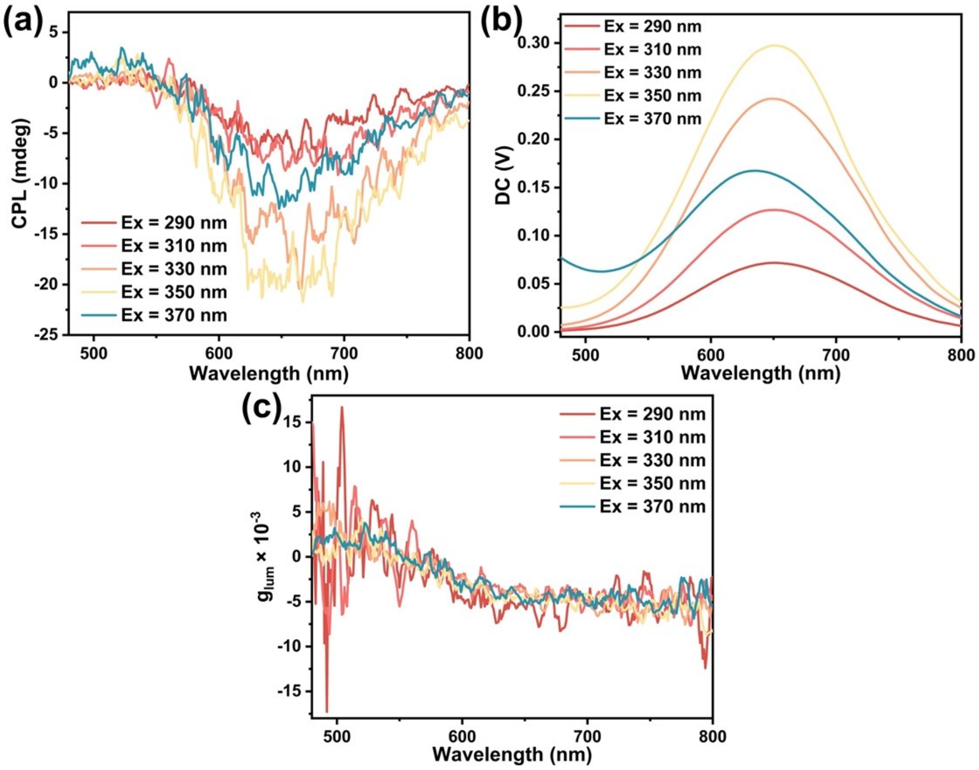

Fig. S66 (a) CPL emission spectra, (b) DC spectra and (c) g_{lum} spectra of **S-2** in crystal state upon different excitation wavelengths.

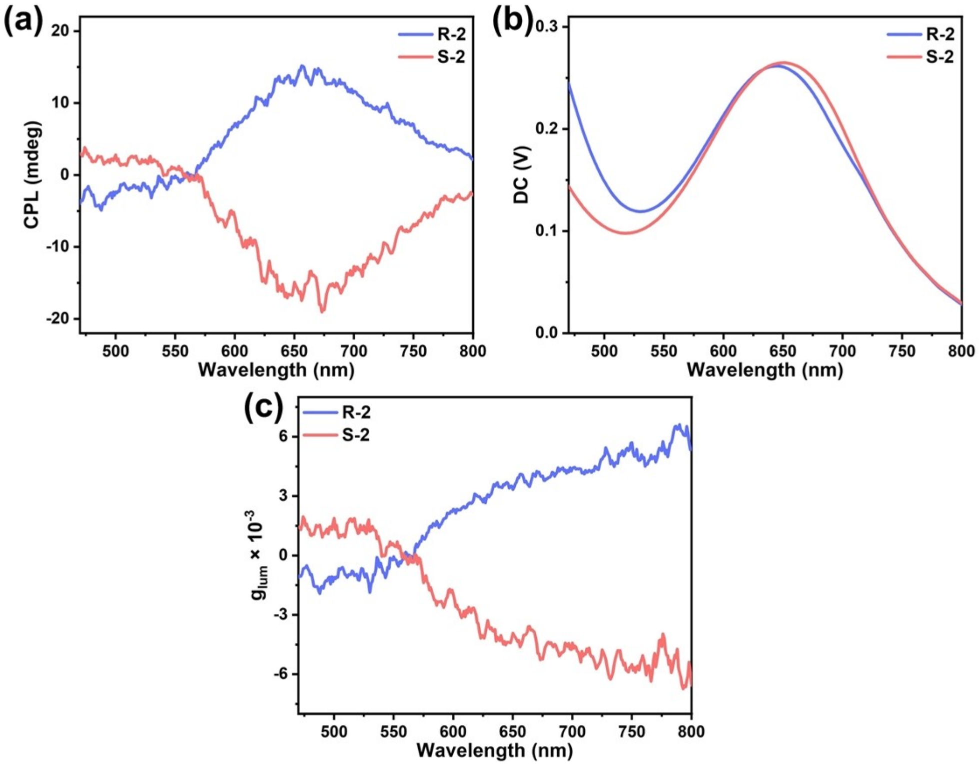

Fig. S67 (a) CPL spectra, (b) DC value and (c) g_{lum} spectra of **R-2** and **S-2** in crystal state upon excitation at 370 nm. The $|g_{lum}|$ values of **R/S-2** were smaller than those of **R/S-1**, which might be attributed to the poor crystallinity of **R/S-2**.[16]

11. Crystal structures modeling of R/S-2

The crystal structures of **R-2/S-2** were analyzed by PXRD experiments combined with theoretical simulations performed in the Materials Studio software package. Simulations of structures similar to **R/S-1**, but with naphthyl groups replacing phenyl groups, were generated. Pawley refinements based on the corresponding stacking mode of **R-2** were performed to give monoclinic space group of $P2_1$ with unit cell parameters of a = 10.6917 Å, b = 20.2477 Å, c = 36.5749 Å, β = 104.4867°, and refinement parameters of R_p = 0.90% and wR_p = 1.50%. Similarly, Pawley refinements based on the corresponding stacking mode of **S-2** were performed to give monoclinic space group of $P2_1$ with unit cell parameters of a = 9.9287 Å, b = 20.9692 Å, c = 35.7146 Å, β = 91.3000°, and refinement parameters of R_p = 1.51% and wR_p = 3.90%. The resulting refined PXRD patterns are in good agreement with the experimental data, as confirmed by the negligible difference curve (Figures S72).

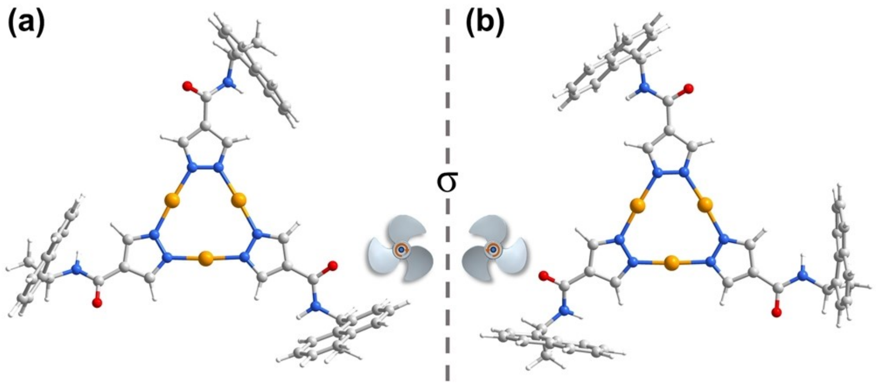

Fig. S68 Molecular structure models of (a) **R-2** and (b) **S-2**. Colour codes: orange, Cu; grey, C; blue, N; red, O; white, H.

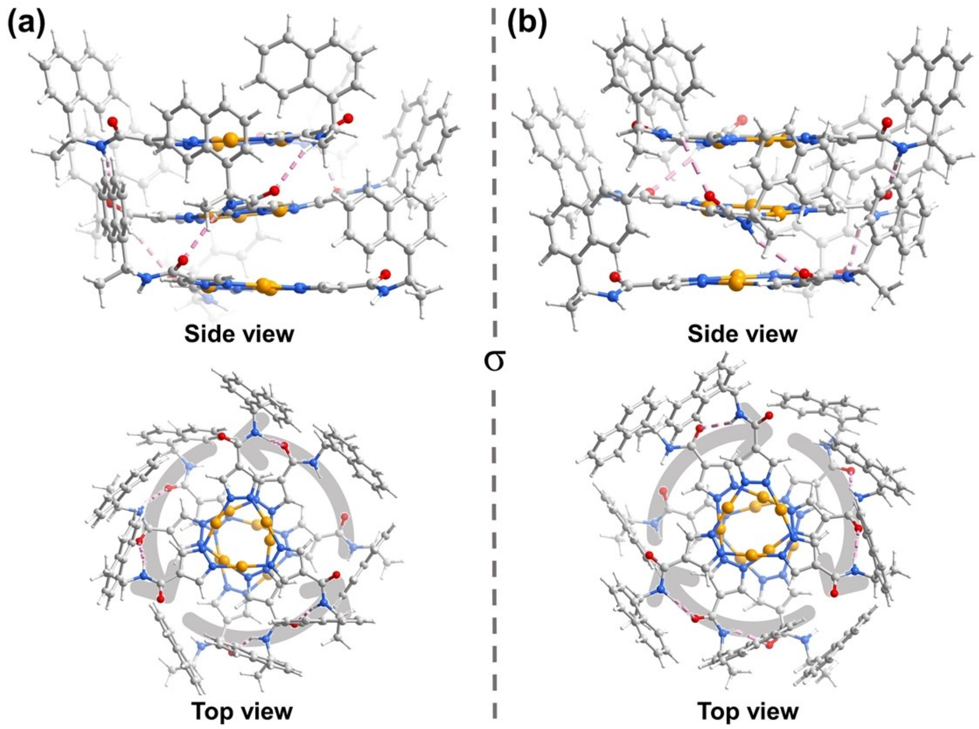

Fig. S69 Structure of stacking models of (a) **R-2** and (b) **S-2**. The pink dashed lines highlight hydrogen-bonding interactions. Colour codes: orange, Cu; grey, C; blue, N; red, O; white, H.

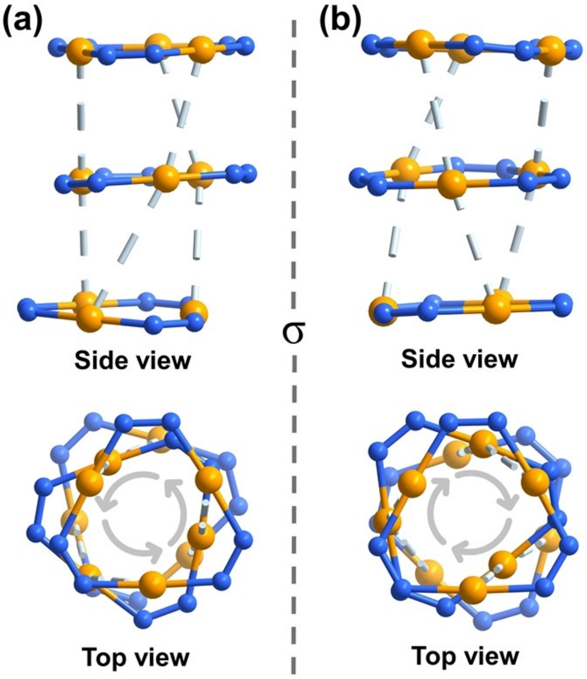

Fig. S70 Stacking model of Cu_3 planes in (a) **R-2** and (b) **S-2**. The blue dashed lines highlight intermolecular Cu···Cu interactions. Colour codes: orange, Cu; blue, N.

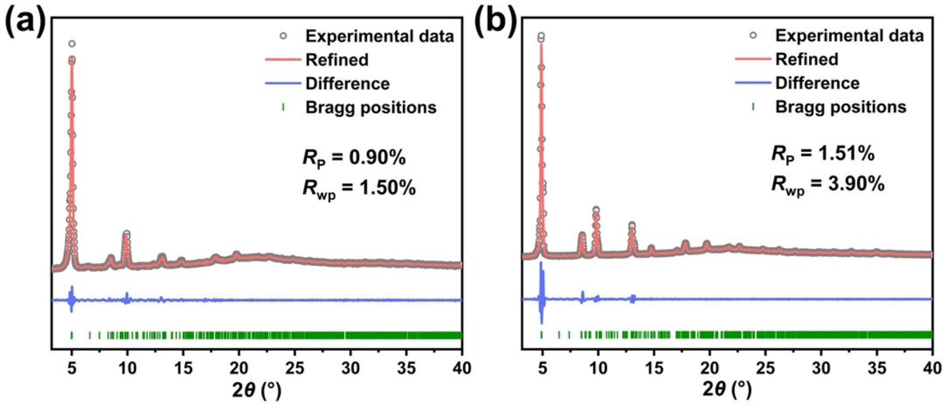

Fig. S71 PXRD structural analysis of (a) **R-2** and (b) **S-2**.

Table S16 Parameter of Spiral stacking model for **R-2**.

Crystal system				monoclinic			
Space group				$P2_1$			
Unit Cell				a = 10.6917 Å, b = 20.2477 Å, c = 36.5749 Å, β = 104.4867°			
Atom	x	y	z	Atom	x	y	z
Cu1	0.5227	0.28965	0.02884	C159	0.35983	1.32499	0.66103
Cu2	0.60831	0.58926	0.25929	C160	0.23863	1.30098	0.66158
Cu3	1.25187	0.56937	0.21752	C161	0.175 93	1.32402	0.68821
Cu4	0.87897	0.37113	0.2556	C162	0.58034	1.33565	0.82228
Cu5	0.85188	0.5002	0.19594	C163	0.63555	1.32747	0.79182
Cu6	0.58976	0.43102	0.28434	C164	0.76686	1.33842	0.79624
Cu7	1.28069	0.48189	0.297	C165	0.84382	1.35864	0.83105
Cu8	0.50863	0.4725	0.19233	C166	0.22277	0.70946	0.60692
Cu9	1.20037	0.40515	0.21285	C167	0.32831	0.68752	0.63493
O10	0.96659	0.39502	0.43327	C168	0.45151	0.68842	0.62897
O11	1.29465	0.7931	0.32204	C169	0.47134	0.713	0.59531
O12	0.41833	0.16802	0.22983	C170	1.26146	0.59128	0.72362
O13	1.03008	0.46084	0.0504	C171	1.25454	0.61316	0.75917
O14	0.41956	0.70542	0.09203	C172	1.13501	0.62269	0.76694
O15	0.61877	0.22477	0.12234	C173	1.02121	0.61103	0.73919
O16	0.86849	0.78975	0.19347	C174	1.1111	0.62225	0.87365
O17	0.66355	0.63011	0.42227	C175	1.11176	0.67735	0.89646
O18	1.19772	0.21996	0.35484	C176	0.99603	0.70595	0.89905
N19	1.14183	0.55495	0.05021	C177	0.87833	0.67806	0.88012
N20	0.98327	0.80597	0.25319	C178	0.54383	0.89045	0.99979
N21	0.4051	0.18135	0.16797	C179	0.57523	0.94632	1.02259
N22	0.75562	0.53216	0.44185	C180	0.7037	0.96343	1.03788
N23	1.40128	0.7404	0.3748	C181	0.80189	0.92465	1.03043
N26	1.02016	0.29265	0.41882	C182	0.99818	1.17857	0.93363
N27	1.23489	0.16093	0.30656	C183	1.03094	1.22181	0.90779
N28	0.56664	0.77204	0.12914	C184	1.15989	1.23325	0.90858
N29	0.52529	0.56495	0.17939	C185	1.25723	1.20164	0.93533
N30	1.20831	0.52233	0.16972	C186	0.70068	0.94319	0.54179
N32	0.49653	0.38172	0.20888	C187	0.67316	0.89571	0.56622
N34	0.81485	0.36064	0.20129	C188	0.55063	0.89248	0.57268
N36	0.59346	0.3045	0.07819	C189	0.45389	0.93595	0.55441
N40	1.18547	0.45822	0.16768	H190	1.21937	0.58367	0.06498
N41	0.53023	0.36561	0.24494	H191	1.04746	0.78489	0.27657
N42	0.94761	0.59505	0.25415	H192	0.41506	0.21343	0.14655
N43	0.97026	0.44728	0.3229	H193	0.80249	0.49107	0.43422
N44	0.64726	0.56281	0.3121	H194	1.4541	0.69832	0.38571
N46	1.30959	0.57651	0.29806	H195	1.05336	0.26351	0.39962

Table S16 continued

Atom	x	y	z	Atom	x	y	z
N50	1.24873	0.38756	0.29288	H196	1.26136	0.16235	0.28093
N55	0.80226	0.41125	0.17793	H197	0.64578	0.77396	0.15254
N57	0.9039	0.58685	0.21738	H198	0.61158	0.35342	0.07233
N62	0.9417	0.38722	0.30947	H199	0.66637	0.44949	0.37445
N65	0.56695	0.61004	0.20564	H200	0.45245	0.08205	0.17853
N70	1.21826	0.35726	0.25993	H201	1.15886	0.05702	0.38681
N75	0.64576	0.50015	0.32171	H202	0.67031	0.65402	0.34354
N95	1.29466	0.61141	0.26683	H203	1.01556	0.48919	0.3788
C24	1.10424	0.50198	0.06822	H204	0.71367	0.4242	0.11888
C25	0.68488	0.5586	0.3742	H205	1.1942	0.59921	0.1285
C31	0.63863	0.28171	0.1145	H206	0.97808	0.89771	0.22692
C33	0.50027	0.7136	0.12181	H207	0.41871	0.32722	0.15779
C35	0.92607	0.7659	0.22357	H208	1.27379	0.35538	0.35031
C37	0.6688	0.49522	0.35936	H209	0.50922	0.21279	0.05775
C38	0.37712	0.11138	0.15872	H210	1.42378	0.8407	0.38823
C39	0.98898	0.35722	0.40973	H211	0.53479	0.25175	−0.01194
C45	1.3411	0.74188	0.33706	H212	0.64797	0.2035	0.02196
C47	1.14941	0.49587	0.10944	H213	0.6752	0.28977	0.01897
C48	1.07491	0.06334	0.36333	H214	1.06399	0.53317	−0.0067
C49	0.66949	0.60059	0.34335	H215	1.21511	0.03465	0.27405
C51	0.98171	0.38143	0.37145	H216	1.27995	−0.00592	0.31885
C52	0.52403	0.66087	0.15054	H217	1.37627	0.05284	0.30123
C53	1.22345	0.28225	0.30389	H218	0.47604	0.95389	0.25635
C54	0.99431	0.44621	0.36071	H219	0.5306	0.82295	0.07777
C56	0.38594	0.29143	0.03062	H220	0.75359	0.25928	0.19492
C58	0.74048	0.39163	0.14301	H221	0.91164	0.31738	0.50829
C59	0.42606	0.20503	0.20415	H222	1.06501	0.29207	0.47775
C60	0.85193	0.08015	0.30474	H223	1.25609	0.90744	0.391
C61	1.18749	0.54733	0.13481	H224	1.11939	0.39041	0.12148
C63	0.71282	0.32477	0.14474	H225	1.59023	0.77963	0.43444
C64	0.96024	0.8773	0.25337	H226	1.46931	0.74292	0.45456
C66	0.45366	0.32761	0.1882	H227	1.49299	0.83113	0.45636
C67	1.2521	0.34364	0.32056	H228	0.94531	0.03734	0.39739
C68	0.52109	0.26319	0.04691	H229	1.29314	0.09764	0.35279
C69	1.21684	0.21942	0.32334	H230	0.34715	0.88305	0.04958
C71	1.39363	0.7949	0.40055	H231	0.32269	0.11994	0.09678
C72	1.33097	0.68124	0.3145	H232	0.37958	0.03979	0.11447
C73	0.59856	0.25205	0.01713	H233	0.49228	0.10664	0.1173
C74	1.07029	0.5763	0.01246	H234	0.62618	0.56414	−0.03701
C76	1.27646	0.04117	0.30307	H235	0.99575	0.67942	0.29024
C77	0.5719	0.9365	0.25635	H236	0.84957	0.53821	−0.03613

Table S16 continued

Atom	x	y	z	Atom	x	y	z
C78	0.52796	0.83306	0.10749	H237	0.45825	0.5687	0.11871
C79	0.93423	0.5994	0.01246	H238	1.17588	0.25639	0.24292
C80	0.7618	0.30684	0.18222	H239	1.03965	0.92906	0.39841
C81	0.8224	0.89349	0.25521	H240	1.01279	0.15886	0.4363
C82	0.83607	0.29358	0.48721	H241	1.05151	0.17228	0.48652
C83	1.0054	0.26264	0.45396	H242	1.16604	0.19438	0.45986
C84	0.24386	0.09146	0.16294	H243	0.29827	0.21259	0.05583
C85	1.20204	0.86686	0.39883	H244	0.92301	0.29333	0.33508
C86	1.14798	0.43991	0.13126	H245	0.79415	0.58759	0.4915
C87	0.9751	0.09136	0.29755	H246	0.74974	0.04933	0.34599
C88	1.49183	0.78623	0.43881	H247	1.15781	0.90197	0.28241
C89	0.39412	0.48888	0.47573	H248	1.05279	0.89282	0.31369
C90	1.08882	0.08287	0.32758	H249	1.04054	0.96475	0.28427
C91	0.95362	0.05173	0.36959	H250	1.09049	0.64145	−0.03259
C92	1.22591	0.09654	0.32411	H251	1.24061	0.60943	−0.00437
C93	0.70069	0.57643	0.41414	H252	1.15848	0.67504	0.01351
C94	0.31157	0.87866	0.07471	H253	0.85382	0.65411	0.1703
C96	0.39337	0.09378	0.11938	H254	0.59514	0.93425	0.10481
C97	0.39256	0.85533	0.10869	H255	0.72242	0.87426	0.1187
C98	0.8643	0.263	0.45574	H256	0.63189	0.90015	0.15166
C99	0.70338	0.58551	−0.0154	H257	0.90971	0.84132	0.41644
C100	0.96636	0.65939	0.26196	H258	0.692	0.31917	0.51621
C100	0.96636	0.65939	0.26196	H258	0.692	0.31917	0.51621
C101	0.83119	0.57148	−0.01489	H259	0.26924	0.53822	0.50541
C102	0.51987	0.48664	0.46913	H260	1.34251	0.60134	0.35676
C103	0.49697	0.59395	0.14519	H261	0.59538	0.71489	0.2046
C104	1.20226	0.29258	0.26529	H262	0.79268	0.95521	0.20536
C105	1.07857	0.87977	0.40295	H263	0.12546	0.91599	0.0462
C106	1.06175	0.19268	0.45924	H264	0.57416	0.63656	0.0108
C107	0.28161	0.25616	0.03821	H265	0.52808	0.27497	0.27548
C108	0.94784	0.34519	0.33822	H266	0.0274	0.34326	−0.01234
C109	1.25635	0.80323	0.40509	H267	0.6452	0.62129	0.52293
C110	1.05415	0.76544	0.41894	H268	0.43037	0.62075	0.53462
C111	0.90541	0.64499	0.03912	H269	0.9364	0.49032	0.50333
C112	1.18091	0.75115	0.41525	H270	0.79899	0.43642	0.49877
C113	0.75084	0.53897	0.48146	H271	0.83779	0.49596	0.53655
C114	0.46142	0.27472	0.21299	H272	0.07609	0.24546	0.02771
C115	0.63768	0.89258	0.28407	H273	0.51407	0.26671	0.46986
C116	0.84295	0.05899	0.34042	H274	1.29305	0.71567	0.25515
C117	0.61283	0.53559	0.48612	H275	0.0324	0.90202	0.10096
C118	1.05863	0.91095	0.28558	H276	0.57537	0.99004	0.20607

Table S16 continued

Atom	x	y	z	Atom	x	y	z
C119	1.14446	0.62908	−0.00345	H277	0.32587	0.0148	0.20332
C120	0.89274	0.64608	0.20025	H278	0.11957	−0.02594	0.21072
C121	0.62526	0.88872	0.12164	H279	−0.08485	0.02063	0.17359
C122	1.00517	0.82971	0.41308	H280	1.11765	1.22627	0.56741
C123	0.93268	0.6937	0.22809	H281	1.03276	1.11314	0.55637
C124	0.71109	0.29465	0.49198	H282	0.8158	1.08634	0.56372
C125	0.36397	0.53679	0.49973	H283	0.67837	1.17095	0.57916
C126	1.33228	0.61712	0.3279	H284	0.51701	1.38824	0.6873
C127	0.35871	0.34979	0.008	H285	0.40527	1.30794	0.6396
C128	0.56825	0.66925	0.18932	H286	0.19254	1.26449	0.64103
C129	0.75149	0.93656	0.22747	H287	0.08096	1.30475	0.68623
C130	0.77385	0.65769	0.03852	H288	0.47736	1.32969	0.81657
C131	0.1844	0.89686	0.07246	H289	0.57583	1.31296	0.76454
C132	0.34295	0.85252	0.14151	H290	0.80822	1.33225	0.77239
C133	0.6745	0.62712	0.01156	H291	0.94545	1.36824	0.83375
C134	0.50997	0.30088	0.24878	H292	0.12996	0.70576	0.6131
C135	0.76359	0.86987	0.28376	H293	0.31469	0.66985	0.66153
C136	0.00642	0.09482	0.14656	H294	0.53133	0.67017	0.6507
C137	0.76036	0.23612	0.4272	H295	0.56835	0.71361	0.59128
C138	0.12583	0.12175	0.14296	H296	1.35559	0.58347	0.71826
C139	0.12724	0.3302	−0.00039	H297	1.34194	0.62208	0.78092
C140	0.57718	0.58375	0.50941	H298	1.1305	0.63926	0.79469
C141	0.45491	0.58388	0.51629	H299	0.93215	0.61765	0.74736
C142	0.83584	0.48702	0.50644	H300	1.20245	0.6013	0.87164
C143	0.15436	0.27484	0.02255	H301	1.20206	0.69776	0.91251
C144	0.61056	0.26541	0.46543	H302	0.99771	0.74957	0.91624
C145	1.30575	0.67596	0.27551	H303	0.79256	0.7018	0.8839
C146	0.22762	0.36825	−0.00763	H304	0.44282	0.88065	0.98853
C147	0.21015	0.86687	0.13794	H305	0.49925	0.9763	1.02853
C148	0.13272	0.88953	0.10356	H306	0.72726	1.00687	1.05553
C149	0.63298	0.23782	0.43257	H307	0.90099	0.93904	1.04236
C150	0.62768	0.95716	0.22785	H308	0.89697	1.17295	0.93188
C151	0.23835	0.03842	0.18738	H309	0.9557	1.24666	0.88704
C152	0.12082	0.01432	0.19145	H310	1.18427	1.26681	0.88845
C153	0.00546	0.0411	0.17054	H311	1.35695	1.21167	0.93578
C154	1.02171	1.21555	0.57068	H312	0.79641	0.94473	0.53688
C155	0.97435	1.15139	0.5644	H313	0.74626	0.86085	0.57998
C156	0.85133	1.13643	0.56823	H314	0.52978	0.85534	0.59158
C157	0.77355	1.18563	0.57773	H315	0.3597	0.92819	0.55902
C158	0.4219	1.37062	0.68799				

Table S17 Parameter of Spiral stacking model for **S-2**.

Crystal system				monoclinic			
Space group				$P2_1$			
Unit Cell				a = 9.9287 Å, b = 20.9692 Å, c = 35.7146 Å, β = 91.3000°			
Atom	x	y	z	Atom	x	y	z
Cu1	0.49977	0.42224	0.27605	C159	0.91135	−0.45894	0.79372
Cu2	0.80553	0.60497	0.24042	C160	0.77769	−0.43942	0.79006
Cu3	1.17249	0.42041	0.22098	C161	0.72303	−0.39758	0.81602
Cu4	0.85841	0.46311	0.28146	C162	0.43488	−0.27389	0.63704
Cu5	1.13527	0.57895	0.21423	C163	0.33168	−0.23367	0.62468
Cu6	0.50613	0.58047	0.29584	C164	0.20399	−0.23939	0.63944
Cu7	0.79786	0.47064	0.1886	C165	0.17874	−0.28555	0.66651
Cu8	1.18115	0.51128	0.29902	C166	1.15998	−0.17235	0.52217
Cu9	0.47852	0.52972	0.20695	C167	1.07066	−0.12166	0.51731
O10	1.17436	0.20742	0.32464	C168	0.93794	−0.12777	0.52846
O11	0.37241	0.82866	0.22274	C169	0.89481	-0.18355	0.5456
O12	0.48427	0.40098	0.44026	C170	0.58122	0.09737	0.53929
O14	0.37841	0.31298	0.11173	C171	0.66315	0.14211	0.55754
O15	0.75767	0.19323	0.20781	C172	0.79981	0.14525	0.55011
O17	1.14731	0.77848	0.35087	C173	0.85579	0.104	0.5241
O21	0.67933	0.72827	0.09695	C174	0.30709	0.29725	0.63202
O23	0.81321	0.61614	0.41994	C175	0.39801	0.3161	0.66008
O41	1.07387	0.52523	0.05347	C176	0.52965	0.33134	0.6514
N13	1.15704	0.8259	0.29463	C177	0.57273	0.32656	0.61465
N16	0.4641	0.80411	0.16755	C178	1.33222	0.42884	0.74367
N18	1.2067	0.25929	0.3792	C179	1.29614	0.40797	0.77903
N19	1.1963	0.41837	0.30117	C180	1.16548	0.38742	0.78524
N20	1.13029	0.52463	0.1703	C181	1.06939	0.3878	0.75614
N22	0.47497	0.61981	0.21953	C182	0.71505	0.24651	0.89398
N24	0.91278	0.18463	0.25442	C183	0.77704	0.19465	0.9118
N25	1.14717	0.46249	0.17284	C184	0.91142	0.19827	0.92356
N26	0.8599	0.3878	0.2498	C185	0.9851	0.25347	0.91712
N27	0.85305	0.54118	0.31066	C186	0.49442	0.0701	1.01424
N28	0.77042	0.55375	0.16622	C187	0.56578	0.0237	1.03462
N29	0.77653	0.60666	0.18614	C188	0.69934	0.03381	1.0455
N30	0.48112	0.43873	0.19683	C189	0.76384	0.08988	1.03542
N31	1.14351	0.62937	0.25973	H190	0.5317	0.77346	0.1546
N32	0.43624	0.23151	0.14975	H191	0.9968	0.20639	0.26703
N35	0.60837	0.48838	0.45298	H192	0.47113	0.21704	0.1762
N37	0.48526	0.64045	0.25434	H193	0.67221	0.52263	0.44173
N38	0.51408	0.45408	0.32709	H194	0.85126	0.09118	0.2437
N39	0.68176	0.64242	0.05909	H195	0.68468	0.59279	0.057

Table S17 continued

Atom	x	y	z	Atom	x	y	z
N40	0.90289	0.70515	0.39598	H196	0.95328	0.72358	0.37316
N42	0.83401	0.59716	0.29451	H197	0.47545	0.9202	0.10014
N43	1.19631	0.38326	0.27056	H198	0.4958	0.83607	0.09187
N49	0.52439	0.51551	0.33474	H199	0.61264	0.87763	0.12257
N61	0.48646	0.39606	0.22391	H200	0.54618	0.56883	0.38594
N62	0.82836	0.39015	0.21381	H201	0.7308	0.53222	0.10837
N69	1.16448	0.60364	0.293	H202	0.42898	0.9026	0.16578
N73	1.1709	0.42829	0.05424	H203	0.63668	0.05954	0.25442
C33	1.22152	0.93917	0.28546	H204	1.07784	0.09586	0.27943
C34	1.19367	0.2578	0.34089	H205	0.95599	0.03874	0.29432
C36	0.86795	0.12267	0.26843	H206	0.98463	0.10925	0.32138
C44	1.20213	0.31707	0.31863	H207	0.47091	0.73367	0.27957
C45	0.50537	0.87418	0.11327	H208	0.74887	0.70585	0.17247
C46	1.1517	0.77282	0.317	H209	0.43795	0.66502	0.16638
C47	0.53553	0.46311	0.38838	H210	0.69899	0.72911	0.02981
C48	0.94973	0.93368	0.34572	H211	0.74967	0.73879	0.4929
C50	1.14108	0.89136	0.30862	H212	0.59185	0.13938	0.10594
C51	1.15336	0.70832	0.3004	H213	0.45494	0.08374	0.10906
C52	0.737	0.12962	0.28978	H214	0.53902	0.11461	0.15072
C53	0.53842	0.5232	0.37195	H215	0.50612	0.36944	0.36074
C54	0.74203	0.56753	0.13028	H216	0.42116	0.06522	0.286
C55	0.42654	0.78905	0.20318	H217	0.71345	0.6526	-0.0333
C56	0.72865	0.63318	0.12743	H218	0.80765	0.60819	0.00156
C57	0.41643	0.86073	0.14692	H219	0.8512	0.68894	-0.00759
C58	0.84929	0.28905	0.22907	H220	0.46533	0.29392	0.22546
C59	0.83793	0.21941	0.22911	H221	0.89464	0.31173	0.28866
C60	0.45157	0.72423	0.21771	H222	0.41591	0.20385	0.09327
C63	0.26855	0.85542	0.13427	H223	0.38689	0.141	0.33707
C64	0.62692	0.09211	0.27767	H224	0.19873	0.22192	0.07624
C65	0.76344	0.93023	0.28585	H225	-0.03452	0.18738	0.07249
C66	0.42017	0.29541	0.14242	H226	-0.00924	0.95196	0.13409
C67	0.9928	0.91042	0.3109	H227	0.49755	0.77718	0.03317
C68	0.25347	0.16364	0.12439	H228	0.66521	0.58439	0.50443
C70	0.21132	0.80222	0.11445	H229	0.79047	0.52271	0.50733
C71	0.72568	0.9531	0.3209	H230	0.67188	0.5318	0.54438
C72	0.97811	0.09025	0.29248	H231	0.81608	0.69284	0.31556
C74	0.4704	0.70435	0.25466	H232	0.51624	0.76697	0.5061
C75	0.58863	0.1796	0.33561	H233	0.61596	0.43971	0.5041
C76	0.45383	0.3443	0.17069	H234	0.8768	0.51018	0.36796
C77	1.12432	0.48187	0.07174	H235	0.78772	0.31883	0.17172
C78	0.75218	0.65662	0.16344	H236	0.062	0.52129	0.52717

Table S17 continued

Atom	x	y	z	Atom	x	y	z
C79	1.04966	0.21163	0.42468	H237	0.94896	0.8335	0.40069
C80	0.45389	0.66916	0.19634	H238	1.08012	0.7937	0.42733
C81	0.85046	0.61314	0.35563	H239	0.95169	0.83785	0.45081
C82	0.71789	0.17412	0.31957	H240	0.92315	0.71927	0.45397
C83	0.8538	0.64443	0.39262	H241	−0.12575	0.1132	0.11854
C84	0.66628	0.67915	0.02426	H242	−0.10869	0.86142	0.10034
C85	0.68596	0.75656	0.47032	H243	0.44813	0.43338	0.13729
C86	0.50149	0.12667	0.1222	H244	0.19981	0.43849	0.55792
C87	0.51782	0.42049	0.35906	H245	0.22629	0.95024	0.15543
C88	0.73748	0.76377	0.43417	H246	0.25798	0.78637	0.01855
C89	0.50317	0.09577	0.29512	H247	0.36496	0.80732	0.45625
C90	0.54121	0.44838	0.42868	H248	0.43701	0.42525	0.54302
C91	0.5181	0.68097	0.01154	H249	0.13539	0.70125	−0.01388
C92	0.89777	0.90889	0.28015	H250	0.94599	−0.17121	0.92803
C93	0.76454	0.65567	-0.00551	H251	1.09447	−0.2193	0.88344
C94	0.47055	0.33738	0.20924	H252	1.34017	−0.21034	0.89108
C95	1.14742	0.43717	0.13834	H253	1.44048	−0.14767	0.943
C96	0.07366	0.80489	0.10236	H254	0.5336	−0.26777	0.6257
C97	1.19909	0.3799	0.33132	H255	0.35025	−0.19844	0.60326
C98	0.91514	0.43807	-0.0187	H256	0.12364	−0.20831	0.6296
C99	0.87431	0.32692	0.26026	H257	0.0778	−0.28836	0.67616
C100	0.69505	0.67086	0.09368	H258	1.26229	−0.16711	0.51287
C101	0.40148	0.18283	0.12157	H259	1.10332	−0.07817	0.50407
C102	0.48395	0.13907	0.32395	H260	0.86727	−0.08932	0.52384
C103	1.13043	0.41127	0.01575	H261	0.78955	−0.18641	0.55137
C104	0.97745	0.40567	0.01157	H262	0.20524	0.29031	0.64043
C105	0.89457	0.37366	0.03787	H263	0.36545	0.31983	0.6887
C106	0.44218	0.50294	0.50304	H264	0.59761	0.34693	0.67336
C107	0.16438	0.18782	0.09667	H265	0.67517	0.33982	0.60827
C108	0.0304	0.16885	0.09451	H266	1.43472	0.44462	0.73948
C109	0.0519	0.91038	0.12882	H267	1.36925	0.40813	0.80179
C110	0.65089	0.78831	0.40515	H268	1.13828	0.37127	0.81284
C111	1.1187	0.54155	0.13415	H269	0.97047	0.37229	0.7632
C112	0.44766	0.73766	0.01916	H270	0.39341	0.05842	1.00522
C113	0.9546	0.16193	0.42077	H271	0.51789	−0.02054	1.04209
C114	0.68448	0.53474	0.51359	H272	0.75388	−0.00255	1.06112
C115	0.832	0.64261	0.321	H273	0.86953	0.09549	1.04267
C116	1.20025	0.32122	0.27986	H274	0.17841	0.81932	0.26651
C117	0.81838	0.95486	0.35053	H275	0.24	0.3019	0.39105
C118	0.55342	0.77271	0.47808	H276	0.19468	0.93661	0.25532
C119	0.06216	0.10468	0.15005	H277	0.33096	0.92931	0.28837

Table S17 continued

Atom	x	y	z	Atom	x	y	z
C120	0.58832	0.48807	0.4936	H278	0.20386	0.98844	0.29566
C121	1.18185	0.20414	0.40381	H279	0.01912	0.93672	0.36948
C122	0.19992	0.12368	0.15279	H280	0.18814	0.89383	0.33715
C123	1.13558	0.69291	0.26284	H281	−0.37632	0.9693	0.32545
C124	0.86253	0.54858	0.34801	H282	0.23083	0.39633	0.06966
C125	1.30168	0.19162	0.4307	H283	0.15835	0.38688	0.13244
C126	0.88167	0.27132	0.46029	H284	0.19395	0.39645	0.35992
C127	1.13126	0.48674	0.11282	H285	−0.02484	0.46219	−0.03935
C128	0.81877	0.33071	0.20003	H286	0.16668	0.44985	−0.00269
C129	0.36424	0.55286	0.48597	H287	0.1038	0.58973	0.12431
C130	0.79113	0.22061	0.45603	H288	−0.0224	0.11941	0.40494
C131	1.01452	0.26624	0.44635	H289	0.19585	0.28225	0.26001
C132	0.77543	0.44073	-0.02266	H290	−0.21173	0.97305	0.37741
C133	0.16756	0.51725	0.52045	H291	0.17423	0.16037	0.38627
C134	0.22491	0.55802	0.49415	H292	0.11855	0.72493	0.23948
C135	1.20078	0.35023	0.00291	H293	0.35399	0.23612	0.43911
C136	1.17098	0.65032	0.3189	H294	0.37654	0.16043	0.41708
C137	0.75202	0.37892	0.03408	H295	0.26787	0.16648	0.4561
C138	0.9715	0.80633	0.42665	H296	−0.30892	0.22335	0.46738
C139	0.82784	0.16626	0.4365	H297	−0.2699	0.46575	−0.04626
C140	0.88493	0.74593	0.42897	H298	0.17596	0.34113	−0.02708
C141	−0.02067	0.12706	0.12075	H299	0.16983	0.3082	0.01943
C142	0.69426	0.41226	0.00387	H300	0.31178	0.35586	0.00596
C143	−0.00407	0.85897	0.10956	H301	0.1869	0.64285	0.34865
C144	0.46179	0.40927	0.1639	H302	−0.24378	0.12777	0.43273
C145	0.24535	0.47042	0.53806	H303	−0.41414	0.41491	0.00056
C146	0.51523	0.80379	0.41354	H304	1.09626	0.54893	0.82509
C147	0.18597	0.9087	0.14109	H305	0.95389	0.50922	0.77338
C148	0.31132	0.74393	0.01031	H306	0.71603	0.54357	0.76688
C149	0.46832	0.79603	0.44988	H307	0.62192	0.61852	0.81055
C150	0.4461	0.6296	−0.00618	H308	0.47579	1.09905	0.54536
C151	0.38035	0.46294	0.52926	H309	0.62022	1.17455	0.5776
C152	0.24175	0.69501	−0.00764	H310	0.86174	1.18018	0.56441
C153	0.30773	0.63829	−0.01652	H311	0.96212	1.108	0.51824
C154	1.05224	−0.16309	0.9321	H312	0.60942	1.24184	0.88781
C155	1.13698	−0.19188	0.90647	H313	0.71903	1.15219	0.91768
C156	1.276	−0.18639	0.9106	H314	0.95794	1.15862	0.9383
C157	1.3318	−0.15097	0.94006	H315	1.0881	1.25608	0.92743
C158	0.99183	−0.43625	0.82315				

12. Literature survey

Table S18 Summary of reported CPL-active coinage metal complexes with orange-red to red emission (λ_{max} > 600 nm) in solid state.

ENTRY	State	λ_{em}	QY (%)	g_{lum} ($\times10^{-3}$)	FM[a] ($\times10^{-3}$)	Ref.
Au complex						
Cl-Au	Crystalline powder	720	70.2	3.4	2.39	17
Br-Au	Crystalline powder	720	72.5	2.7	1.96	
R-Au$_{13}$-NHC	Solid	750	66	2.65	1.75	18
S-Au$_{13}$-NHC	Solid	750	66	−2.65	1.75	
R-Au$_4$(HMMT)$_4$-red	Solid	727	24.5	−1.9	0.47	19
S-Au$_4$(HMMT)$_4$-red	Solid	727	24.5	1.3	0.32	
Ag complex						
[Ag$_{17}$(*R*-NYA)$_{12}$](NO$_3$)$_3$	Solid	745	8	1.2	0.10	20
[Ag$_{17}$(*S*-NYA)$_{12}$](NO$_3$)$_3$	Solid	745	8	−1.2	0.10	
R-DHLA	Powder	660	7.6	2	0.15	21
S-DHLA	Powder	660	7.6	−2	0.15	
[Ag$_7$(R-DMA)$_2$(dpppy)$_3$](BF$_4$)$_3$	Solid	675	8.6	2	0.17	22
[Ag$_7$(S-DMA)$_2$(dpppy)$_3$](BF$_4$)$_3$	Solid	675	9.1	−2	0.18	
{[(Ag$_{12}$(S^{i}Pr)$_6$(D-CSA)$_6$(An2Py)$_3$)]·(H$_2$O)$_2$}$_n$	Solid	612	50.3	12	6.04	23
{[(Ag$_{12}$(S^{i}Pr)$_6$(L-CSA)$_6$(An2Py)$_3$)]·(H$_2$O)$_2$}$_n$	Solid	612	50.3	−12	6.04	
[Ag$_{12}$(S^{i}Pr)$_6$(D-CSA)$_8$]·(H$_2$An2Py)(solvent)$_x$	Solid	630	7.6	1.3	0.10	
[Ag$_{12}$(S^{i}Pr)$_6$(L-CSA)$_8$]·(H$_2$An2Py)(solvent)$_x$	Solid	630	7.6	−1.3	0.10	
Cu complex						
(R-MBA)$_4$Cu$_4$I$_4$	Powder	630	52.8	10	5.28	24
(S-MBA)$_4$Cu$_4$I$_4$	Powder	630	59.7	−6	3.58	
[Cu$_{14}$(R-DPM)$_8$](PF$_6$)$_6$	Solid	726	8.2	3.0	0.25	25
[Cu$_{14}$(S-DPM)$_8$](PF$_6$)$_6$	Solid	726	8.2	−3.0	0.25	
R-2	Solid	610	5	+0.8	0.04	26
S-2	Solid	610	5	−0.8	0.04	
[D-valinol(18-crown-6)]$^+$[Cu$_5$(S^{t}Bu)$_6$]$^-$	Solid	660	47.4	9.77	4.63	27
[L-valinol(18-crown-6)]$^+$[Cu$_5$(S^{t}Bu)$_6$]$^-$	Solid	660	47.4	−9.77	4.63	
(Cu$_6$Br$_6$)-R	Crystalline	637	1.12	7.0	0.08	28
(Cu$_6$Br$_6$)-S	Crystalline	656	1.13	7.0	0.08	
Cu$_5$I$_7$(L$_4$)$_2$	Polycrystals	620	6	6	0.36	29
R-Cu$_2$I$_2$	Solid	675	<1	2.2	<0.02	30
S-Cu$_2$I$_2$	Solid	675	<1	−2.8	<0.03	
(S)-Cu$_4$I$_4$(Hmpy)$_4$·H$_2$O	Crystal	610	93.2	−6.7	6.24	31
(R)-Cu$_4$I$_4$(Hmpy)$_4$·H$_2$O	Crystal	610	94.8	+6.3	5.97	

(S)-$Cu_4I_4(3Hpy)_4$	Crystal	646	83.8	−3.2	2.68	
(R)-$Cu_4I_4(3Hpy)_4$	Crystal	646	78.7	+2.0	1.57	
(S)-$Cu_4I_4(3AD)_4$	Crystal	609	42.3	−3.4	1.44	
(R)-$Cu_4I_4(3AD)_4$	Crystal	609	42.4	+2.6	1.10	
{[(CuI)(Hptdp)]$_4$·H_2O}$_n$-M	Single crystals	501, 605	2.4	−8.0	0.19	32
{[(CuI)(Hptdp)]$_4$·H_2O}$_n$-P	Single crystals	501, 605	2.4	8.0	0.19	
{Cu(Cbz^R)[(***R***)-BINAP]} [R = 3,6-*t*Bu]	Ground	606	53	21	11.13	33
{Cu(Cbz^R)[(***S***)-BINAP]} [R = 3,6-*t*Bu]	Ground	606	53	21	11.13	
R-CS1	Solid-state	776	--	--	--	34
S-CS1	Solid-state	776	--	--	--	
R-CS2	Solid-state	858	--	--	--	
S-CS2	Solid-state	858	--	--	--	
Mix-metal complex						
$[Cu_{15}Ag_4(R\text{-PEA})_{12}](BF_4)_5$	Crystal	626	7.02	1.0	0.07	35
$[Cu_{15}Ag_4(S\text{-PEA})_{12}](BF_4)_5$	Crystal	626	7.02	−1.0	0.07	
R-py-Br	Solid	616	57.9	1.5	0.87	36
R-py-I	Solid	631	45.3	0.6	0.27	
R-py-Cl	Solid	628	5.4	0.7	0.04	
R-py-Br	Solid	600	62.8	1.5	0.94	
R-py-I	Solid	613	93	1.1	1.02	
SCIF-2-Left	Crystal	652	5.49	3	0.16	37
SCIF-2-Right	Crystal	650	5.49	−3	0.16	

[a]FM (figure of merit) = PLQY×$|g_{lum}|$

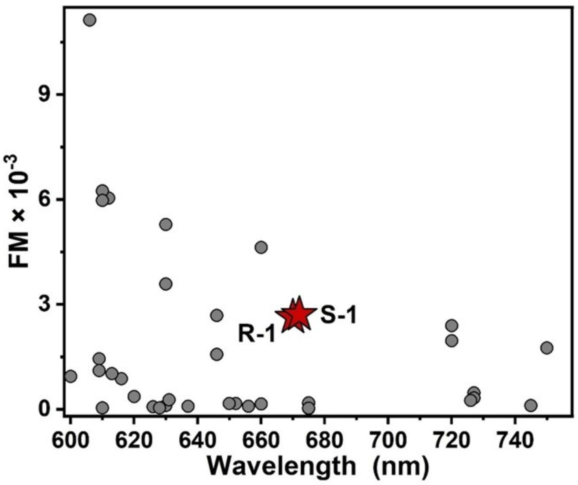

Fig. S72 Summary of the reported FM values of coinage metal complexes in solid state with emission wavelength exceeding 600 nm.

13. Application showcase

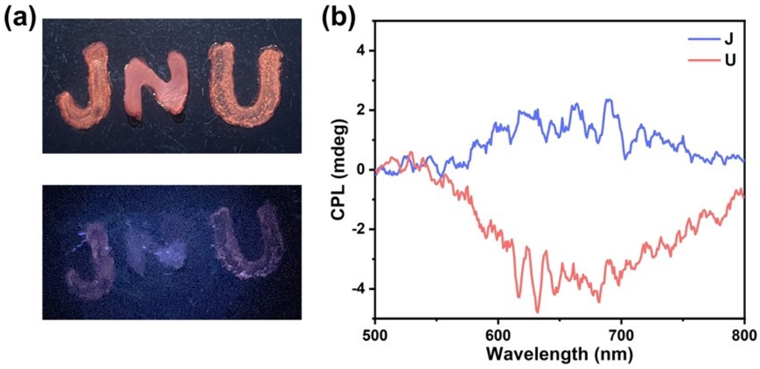

Fig. S73 (a) The photographs of "**JNU**" pattern under 310 nm (top) and 365 nm (bottom) UV lamp, and. (b) CPL spectrum of "**J**" and "**U**" under 310 nm.

Note: Inspired by the sophisticated and multiplexed photoluminescence characteristics exhibited by **R-2**/**S-2**, their potential as advanced anti-counterfeiting materials were further explored. The synthesized polymeric films of **R-2**@PMMA, **S-1**@PMMA, and **S-2**@PMMA were fabricated into "**J**-", "**N**-", and "**U**-shaped" configurations, respectively. As shown in Figure S72a, the "**JNU**" patterns displayed orange-red emission under 310 nm UV lamp irradiation with uniform chromatic fidelity. Notably, when illuminated by a 365 nm UV lamp, the **S-1**@PMMA film exhibited negligible emission, while the **R-2**@PMMA and **S-2**@PMMA films demonstrated violet emission, revealing encrypted letters "**J**" and "**U**". Moreover, as the "**J**" and "**U**" patterns originated from enantiomeric counterparts **R-2** and **S-2**, respectively, they manifested distinct CPL signals (Figure S72b).

14. References

1 Y. Yan, E. J. Carrington, R. Pétuya, G. F. S. Whitehead, A. Verma, R. K. Hylton, C.-C. Tang, N. G. Berry, G. R. Darling, M. S. Dyer, D. Antypov, A. P. Katsoulidis and M. J. Rosseinsky, *J. Am. Chem. Soc.*, 2020, **142**, 14903–14913.

2 G. M. Sheldrick, *Acta Cryst. C*, 2015, **71**, 3–8.

3 C.-Y. Li, H. Xu, P.-M. Cheng, M.-H. Du, L.-S. Long, L.-S. Zheng and X.-J. Kong, *J. Am. Chem. Soc.*, 2023, **145**, 22176–22183

4 M. J. Frisch, G. W. Schlegel, H. B. Trucks, G. E. Scuseria, M. A. Robb, J. R. Cheeseman, G. Scalmani, V. Barone, B. Mennucci, G. A. Petersson, H. Nakatsuji, M. Caricato, X. Li, H. P. Hratchian, A. F. Izmaylov, J. Bloino, G. Zheng, J. L. Sonnenberg, M. Hada, M. Ehara, K. Toyota, R. Fukuda, J. Hasegawa, M. Ishida, T. Nakajima, Y. Honda, O. Kitao, H. Nakai, T. Vreven, J. A. Jr. Montgomery, J. E. Peralta, F. Ogliaro, M. Bearpark, J. J. Heyd, E. Brothers, K. N. Kudin, V. N. Staroverov, T. Keith, R. Kobayashi, J. Normand, K. Raghavachari, A. Rendell, J. C. Burant, S. S. Iyengar, J. Tomasi, M. Cossi, N. Rega, J. M. Millam, M. Klene, J. E. Knox, J. B. Cross, V. Bakken, C. Adamo, J. Jaramillo, R. Gomperts, R. E. Stratmann, O. Yazyev, A. J. Austin, R. Cammi, C. Pomelli, J. W. Ochterski, R. L. Martin, K. Morokuma, V. G. Zakrzewski, G. A. Voth, P. Salvador, J. J. Dannenberg, S. Dapprich, A. D. Daniels, O. Farkas, J. B. Foresman, J. V. Ortiz, J. Cioslowski and D. J. Fox, Gaussian, Inc., Wallingford CT, 2013. Gaussian 09, Revision E.01., 2009.

5 T. Lu and F. Chen, *J. Comput. Chem.*, 2012, **33**, 580–592.

6 J. P. Perdew, K. Burke and M. Ernzerhof, *Phys. Rev. Lett.*, 1996, **77**, 3865−3868.

7 J. P. Perdew, K. Burke and M. Ernzerhof, [Phys. Rev. Lett., **77**, 3865 (1996)]. *Phys. Rev. Lett.*, 1997, **78**, 1396.

8 S. Grimme, J. Antony, S. Ehrlich and H. A. Krieg, *J. Chem. Phys.*, 2010, **132**, 154104.

9 S. Grimme, S. Ehrlich and L. Goerigk, *J. Comput. Chem.*, 2011, **32**, 1456−1465.

10 P. J. Hay and W. R. Wadt, *J. Chem. Phys.*, 1985, **82**, 299–310.

11 W. R. Wadt and P. Hay, J. *Chem. Phys.*, 1985, **82**, 284–298.

12 L. Radom, P. C. Hariharan, J. A. Pople and P. V. R. Schleyer, *J. Am. Chem. Soc.*, 1973, **95**, 6531–6544.

13 M. J. Frisch, J. A. Pople and J. S. Binkley, *J. Chem. Phys.*, 1984, **80**, 3265–3269.

14 T. Lu and Q. Chen, *J. Comput. Chem.*, 2022, **43**, 539–555.

15 W. Humphrey and A. Dalke, K. Schulten, *J. Mol. Graphics*, 1996, **14**, 33–38.

16 C. Zhang, S. Li, X.-Y. Dong and S.-Q. Zang, *Aggregate*, 2021, **2**, e48.

17 H. Yang, S.-K. Peng, J. Zheng, D. Luo, M. Xie, Y.-L. Huang, X. Cai, J. Wang, X.-P. Zhou and D. Li, *Angew. Chem. Int. Ed.*, 2023, **62**, e202310495.

18 P. Luo, X.-J. Zhai, S. Bai, Y.-B. Si, X.-Y. Dong, Y.-F. Han and S.-Q. Zang, *Angew. Chem. Int. Ed.*, 2023, **62**, e202219017.

19 S.-M. Zhai, H. Zhang, Y. Wang, L.-X. Zhang, W.-Y. Jiao, Y.-Q. Zhang, Y. Si, H.-Y. Li, S.-Q. Zang and Z. Han, *Angew. Chem. Int. Ed.*, 2025, e202502168.

20 M.-M. Zhang, X.-Y. Dong, Z.-Y. Wang, X.-M. Luo, J.-H. Huang, S.-Q. Zang and T. C. W. Mak, *J. Am. Chem. Soc.*, 2021, **143**, 6048–6053.

21 J. Kumar, T. Kawai and T. Nakashima, *Chem. Commun.*, 2017, **53**, 1269–1272.

22 W.-M. He, J. Zha, Z. Zhou, Y.-J. Cui, P. Luo, L. Ma, C. Tan and S.-Q. Zang, *Angew. Chem. Int. Ed.*, 2024, **63**, e202407887.

23 J.-Y. Wang, Y. Si, X.-M. Luo, Z.-Y. Wang, X.-Y. Dong, P. Luo, C. Zhang, C. Duan and S.-Q. Zang, *Adv. Sci.*, 2023, **10**, 2207660.

24 L. Yao, G. Niu, J. Li, L. Gao, X. Luo, B. Xia, Y. Liu, P. Du, D. Li, C. Chen, Y. Zheng, Z. Xiao and J. Tang, *J. Phys. Chem. Lett.*, 2020, **11**, 1255–1260.

25 M.-M. Zhang, X.-Y. Dong, Z.-Y. Wang, H.-Y. Li, S.-J. Li, X. Zhao and S.-Q. Zang, *Angew. Chem. Int. Ed.*, 2020, **59**, 10052 –10058.

26 Y. Jin, Q.-C. Peng, J.-W. Xie, K. Li and S.-Q. Zang, *Angew. Chem. Int. Ed.*, 2023, **62**, e202301000.

27 Y. Jin, S. Li, Z. Han, B.-J. Yan, H.-Y. Li, X.-Y. Dong and S.-Q. Zang, *Angew. Chem. Int. Ed.*, 2019, **58**, 12143–12148.

28 J.-J. Fang, Z. Liu, Y.-L. Shen, Z.-Y. Wang, Y.-P. Xie and X. Lu, *ACS Materials Lett.*, 2024, **6**, 1199–1206.

29 L.-Z. Feng, J.-J. Wang, T. Ma, Y.-C. Yin, K.-H. Song, Z.-D. Li, M.-M. Zhou, S. Jin, T. Zhuang, F.-J. Fan, M.-Z. Zhu and H.-B. Yao, *Nat. Commun.*, 2022, **13**, 3339.

30 Z.-Y. Wu, M.-X. Yu, Z.-Q. Zhang, J.-X. Jiang, T. Liu, F.-L. Jiang, L. Chen and M.-C. Hong, *Dalton Trans.*, 2024, **53**, 7315–7320.

31 X. Ji, Y. Liu, R. Li, Z. Zhang, X. Zhang, C. Chen, J. Chen, H. Lu, R. Chen and L.

Mao, *Adv. Opt. Mater.*, 2023, **11**, 2300541.

32 M.-X. Yu, C.-P. Liu, Y.-F. Zhao, S.-C. Li, Y.-L. Yu, J.-Q. Lv, L. Chen, F.-L. Jiang and M.-C. Hong, *Angew. Chem. Int. Ed.*, 2022, **61**, e202201590.

33 A. M. T. Muthig, O. Mrózek, T. Ferschke, M. Rödel, B. Ewald, J. Kuhnt, C. Lenczyk, J. Pflaum and A. Steffen, *J. Am. Chem. Soc.*, 2023, **145**, 4438–4449.

34 Z. Han, Y. Si, X.-Y. Dong, J.-H. Hu, C. Zhang, X.-H. Zhao, J.-W. Yuan, Y. Wang and S.-Q. Zang, *J. Am. Chem. Soc.*, 2023, **145**, 6166–6176.

35 M.-M. Zhang, K.-K. Gao, X.-Y. Dong, Y. Si, T. Jia, Z. Han, S.-Q. Zang and T. C. W. Mak, *J. Am. Chem. Soc.*, 2023, **145**, 22310–22316.

36 X.-H. Ma, J. Li, P. Luo, J.-H. Hu, Z. Han, X.-Y. Dong, G. Xie and S.-Q. Zang, *Nat. Commun.*, 2023, **14**, 4121.

37 S. Chen, W. Du, C. Qin, D. Liu, L. Tang, Y. Liu, S. Wang and M. Zhu, *Angew. Chem. Int. Ed.*, 2020, **59**, 7542–7547.
